# Identification of Immune-Related Gene Signatures in Lung Adenocarcinoma and Lung Squamous Cell Carcinoma

**DOI:** 10.3389/fimmu.2021.752643

**Published:** 2021-11-23

**Authors:** Na Li, Jiahong Wang, Xianquan Zhan

**Affiliations:** ^1^ Shandong Key Laboratory of Radiation Oncology, Shandong Cancer Hospital and Institute, Shandong First Medical University, Jinan, China; ^2^ Medical Science and Technology Innovation Center, Shandong First Medical University, Jinan, China; ^3^ Cancer Research Institute, School of Basic Medical Sciences, Southern Medical University, Guangzhou, China; ^4^ Gastroenterology Research Institute and Clinical Center, Shandong First Medical University, Jinan, China

**Keywords:** lung cancer, tumor microenvironment, immune-related gene signature, clinical characteristics, tumor mutation burden

## Abstract

Accumulating evidence indicates that immunotherapy helped to improve the survival and quality-of-life of patients with lung adenocarcinoma (LUAD) or lung squamous cell carcinoma (LUSC) besides chemotherapy and gene targeting treatment. This study aimed to develop immune-related gene signatures in LUAD and LUSC subtypes, respectively. LUAD and LUSC samples were divided into high- and low-abundance groups of immune cell infiltration (Immunity_H and Immunity_L) based on the abundance of immune cell infiltrations. The distribution of immune cells was significantly different between the high- and low-immunity subtypes in LUAD and LUSC samples. The differentially expressed genes (DEGs) between those two groups in LUAD and LUSC contain some key immune-related genes, such as PDL1, PD1, CTLA-4, and HLA families. The DEGs were enriched in multiple immune-related pathways. Furthermore, the seven-immune-related-gene-signature (CD1B, CHRNA6, CLEC12B, CLEC17A, CLNK, INHA, and SLC14A2) prognostic model-based high- and low-risk groups were significantly associated with LUAD overall survival and clinical characteristics. The eight-immune-related-gene-signature (C4BPB, FCAMR, GRAPL, MAP1LC3C, MGC2889, TRIM55, UGT1A1, and VIPR2) prognostic model-based high- and low-risk groups were significantly associated with LUSC overall survival and clinical characteristics. The prognostic models were tested as good ones by receiver operating characteristic, principal component analysis, univariate and multivariate analysis, and nomogram. The verifications of these two immune-related-gene-signature prognostic models showed consistency in the train and test cohorts of LUAD and LUSC. In addition, patients with LUAD in the low-risk group responded better to immunotherapy than those in the high-risk group. This study revealed two reliable immune-related-gene-signature models that were significantly associated with prognosis and tumor microenvironment cell infiltration in LUAD and LUSC, respectively. Evaluation of the integrated characterization of multiple immune-related genes and pathways could help to predict the response to immunotherapy and monitor immunotherapy strategies.

## Introduction

Lung cancer is the most common malignant tumor worldwide, and its incidence and mortality have been increasing year by year among men and women, which has caused a serious burden on patients and society. The highest incidence and mortality rates of lung cancer were distributed in North America, Europe, and East Asia (1). Lung cancer is divided into small cell lung cancer (15%) and non-small cell lung cancer (NSCLC, 75%) according to cancer cell type. Three main subtypes of NSCLC are lung adenocarcinoma (LUAD, 40%), lung squamous cell carcinoma (LUSC, 30%), and large cell carcinoma (15%) (2). Depending on the different types, the treatment and prognosis were also different between LUAD and LUSC. With the development of gene detection technology, the main driver genes of different kinds of lung cancers have been identified—for example, EGFR mutations, ALK fusion, ROS-1 fusion, PTEN mutations, FGFR1 amplification, c-Met amplification, and KRAS mutations (3). LUAD has a sensitive mutation-targeted treatment plan compared to LUSC, but a patient’s prognosis with advanced NSCLC is still very poor and the 5-year survival rate might be <15% (4). Whether it is LUAD or LUSC, in addition to chemotherapy and targeted drugs, immunotherapy was proved to be safe and effective (5). There are currently three checkpoint inhibitors that targeted PD-1/PD-L1 and are approved for lung cancer: nivolumab (Opdivo), pembrolizumab (Keytruda), and atezolizumab (Tecentriq) (6). Those checkpoint drugs brought hope and relief to patients with chemotherapy resistance, advanced tumors, or driver gene negative. The development of multiple immune biomarkers or immune-related gene signature would contribute to predict immunotherapeutic outcomes.

Increasing evidence showed that immune-related cells, molecules, cytokines, and pathways were closely associated with the biological property of carcinoma. The classification of tumor antigen, antitumor and application of immunotherapy, and mechanism of tumor cells escaping the immune system became a research hotspot in immunotherapy strategy (7). In terms of immune-related cells, the percentages of circulating PD-1^+^ CD137^+^ CD8^+^ T cell and CD137^+^ CD8^+^ T cell subsets among CD8^+^ T cells were positively correlated with thoracic tumor burden and the percentage of effector regulatory T cell (Treg) subset. These findings showed the interactions between immune and host in lung cancer patients in the level of peripheral blood and further suggested that the differential control of activation of tumor-specific effector T cells and Treg should be used as immunotherapeutic intervention strategies (8). Myeloid-derived suppressor cell (MDSC)-mediated tumor immunosuppressive environments regulated the immunosuppressive environments in a tumor microenvironment (TME), and findings revealed that MDSC development and differentiation could be induced by chemotherapy through IL-13/IL-33-mediated pathway in lung cancer (9). When immune-based strategies are adopted, the immune mechanisms impacted by chemotherapy should be considered to restore immunity-related activity and inhibit the immunosuppressive phenotype of lung cancer (9). In terms of immune molecules, it was reported that those against the PD-1/PD-L1 checkpoint could improve the survival rates with KRAS-mutant NSCLC patients because KRAS mutations were correlated with tumor immunogenicity and an inflammatory TME (10). The FDA has approved the combination of pembrolizumab, pemetrexed, and carboplatin for the first-line treatment of metastatic non-squamous NSCLC, regardless of PD-L1 expression (11). For patients with advanced or squamous cell carcinoma with low or no PD-L1 expression, a combination therapy of pembrolizumab and carboplatin/taxanes could be a better choice (12). In terms of immune cytokines, NSCLC patients (*n* = 18) and control lung tissue samples (*n* = 5) were used to determine the relationship between malignant progression and IL-11 expression, which indicated that IL-11 was an oncogene in NSCLC. IL-11 promoted cell proliferation, migration, invasion, epithelial–mesenchymal transition, and tumorigenesis and activated AKT and STAT3 (13). Tumor-associated antigens activated anti-tumor immune responses through the recognition of cancer cells by T cells when presented at a sufficient level (14). In terms of immune-related pathways, transcriptomics analysis showed that radiation treatment could upregulate the expressions of many genes in antigen processing and presentation pathways in all cell lines, which revealed the immunostimulatory role of cancer radiotherapy *via* antigen processing and presentation pathways (15). Recently, studies also demonstrate that tumor mutation burden (TMB) induced antigen exposure, which is a promising biomarker to select NSCLC patients for immunotherapy, specifically in the gene mutation of MET, RET, HER2, and KRAS (16). Immunotherapy might be an effective approach to improve the survival and quality-of-life of lung cancer patients. However, the comprehensive landscape of immunotherapy and immune-related gene signatures remains unclear.

**Figure 1:**
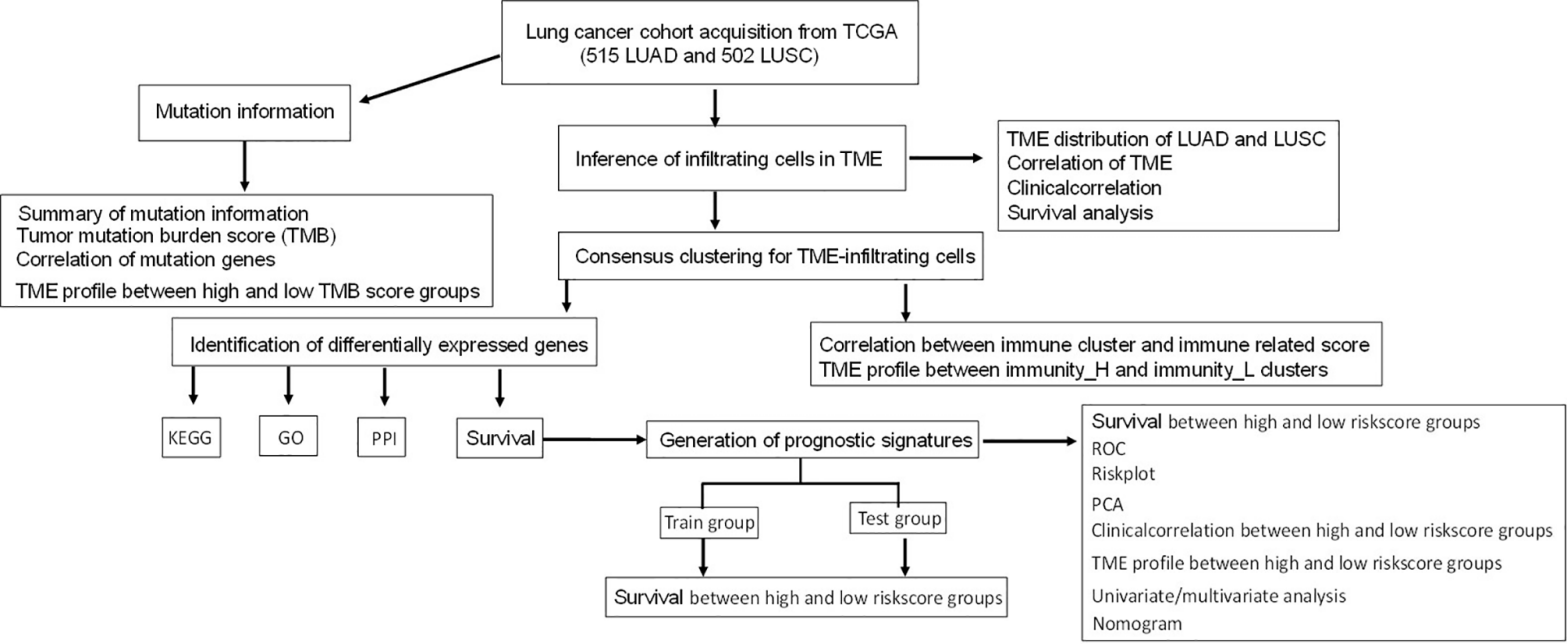
Flow chart for the identification of immune signatures in lung adenocarcinoma or lung squamous cell carcinoma subtypes of lung cancers.

Most studies analyzed different genes between tumor and normal tissues and did LASSO analysis to construct immune gene signatures (17–19). In this study, consensuClusterPlus R package was used to perform cluster analysis, which was cycle computed for 1,000 times to guarantee the reliability and stability of classification. It would be a more direct approach than tissue types (tumor and normal tissues) to show immunotherapy response. One previously published article also used consensuClusterPlus R package to divide LUAD samples in The Cancer Genome Atlas (TCGA)-LUAD into high-, medium-, and low-immune infiltration groups and obtained different expressed lncRNAs between high- and low-immune infiltration groups to construct an eight-immune-related-lncRNA (AL365181.2, AC012213.4, DRAIC, MRGPRG-AS1, AP002478.1, AC092168.2, FAM30A, and LINC02412) prognostic signature (20). However, our study systematically plotted the distribution of immune cells in lung cancer (including LUAD and LUSC) and their relationship with clinicopathological characteristics. Additionally, gene mutation information showed that the distribution of immune cells was significantly related to TMB. The LUAD and LUSC samples were divided into high- and low-immunity subtypes based on the abundance of a panel of immune cell infiltrations, respectively. Furthermore, LASSO regression was used to construct the seven-gene-signature (CD1B, CHRNA6, CLEC12B, CLEC17A, CLNK, INHA, and SLC14A2) prognostic model in LUAD, and the high- and low-risk groups, based on this signature model, were significantly correlated with LUAD overall survival and clinical characteristics. LASSO regression was also used to construct the eight-gene-signature (C4BPB, FCAMR, GRAPL, MAP1LC3C, MGC2889, TRIM55, UGT1A1, and VIPR2) prognostic model in LUSC, and the high- and low-risk groups based on this signature model were significantly correlated with LUSC overall survival and clinical characteristics. This study revealed two reliable gene signature models that were significantly associated with prognosis and TME cell infiltration in LUAD and LUSC, respectively, which can promote individualized treatment and offer potential new targets of immunotherapy. A simplified sequence flow diagram (**Figure 1**) was shown to identify immune signatures in LUAD and LUSC subtypes of lung cancers.

## Materials and Methods

### Lung Cancer RNA-seq Data and Clinical Data

Lung cancer RNA-seq data, clinical follow-up data (**Supplementary Tables S1** and **S2**), and mutation data (**Supplementary Tables S3** and **S4**) in LUAD and LUSC (https://xena.ucsc.edu/) were obtained from the public database TCGA (https://portal.gdc.cancer.gov/), respectively. Genomic Data Commons can help obtain high-quality standardized clinical and molecular data, easily conduct high-performance search, directly download and analyze clinical information and genomic characteristic data, and conduct high-level sequence analysis of tumor genomic data. RNA-seq data were required to be from patients with complete clinical information and survival status data. Thus, 515 LUAD and 502 LUSC tissue samples were selected for this study. Next, the Tumor Immune Dysfunction and Exclusion (TIDE) (http://tide.dfci.harvard.edu/) was used to predict the response to immunotherapy based on the simulation of tumor immune escape mechanism. In this study, the response of the TCGA-A cohort to immunotherapy based on TIDE algorithm helps divide lung cancer samples into non-responder and responder groups.

### Gene Expression Data-Based TME Immune Cell Infiltration Abundance

The single-sample Gene Set Enrichment Analysis (ssGSEA) was used to quantify the abundance of TME immune cell infiltration with GSEABase R package based on RNA-seq gene expression data (21). A total of 28 human TME immune cell subtypes were analyzed, including central memory (Tcm), activated T cells, gamma delta T (Tγδ) cells, effector memory (Tem) CD4^+^/CD8^+^ T cells, Th2 cells, T helper 1 (Th1) cells, Th17 cells, follicular helper T cells (Tfh), regulatory T cells (Treg), the activated, immature, and memory B cells, and innate immunity-related cell types such as monocytes, mast cells, macrophages, eosinophils, neutrophils, the activated plasmacytoid and immature dendritic cells (DCs), NK cells, natural killer T (NKT) cells, and MDSCs (**Supplementary Tables S5** and **S6**).

### High- and Low-Immunity Clusters of Lung Cancer Tissues

Lung cancer tissues with TME immune cell infiltration abundance were clustered with hierarchical agglomerative consensus clustering according to Ward’s linkage and Euclidean distance. The proportion of ambiguous clustering-based unsupervised clustering methods was a simple and powerful method to infer optimal K (K-means) that classified patients for further analysis. The ConsensuClusterPlus R package was used to perform cluster analysis, which was cycle-computed for 1,000 times to guarantee the reliability and stability of classification. A consensus clustering analysis was performed on cancer tissue samples according to the scores of ssGSEA to obtain the most stable group. Based on TME immune cell infiltration abundance, the LUAD and LUSC tissue samples were divided into high (H)- and low (L)-abundance groups of a panel of immune cell infiltrations (Immunity_H and Immunity_L), respectively (**Supplementary Tables S7** and **S8**).

### Estimation of Tumor Purity and Infiltrating Cells in LUAD and LUSC

Tumor purity and infiltrating stromal and immune cells in tumor tissues were predicted with ESTIMATE R package, which can estimate stromal and immune cells in malignant tumor tissues with gene expression data (**Supplementary Tables S9** and **S10**). The ESTIMATE algorithm was based on ssGSEA analysis, which generated an immune score that represented the status of infiltrating immune cells in tumor tissue, stromal score that captured the status of stromal cells in tumor tissue, and ESTIMATE score that inferred tumor purity. Those three scores were positively correlated with the ratio of immune, stromal, and sum of both, respectively; and the higher score reflects the larger ratio of the corresponding component in TME (22).

### The Proportions of Immune Cells in LUAD and LUSC Determined With CIBERSORT Method

The proportion of immune cells in the LUAD and LUSC tissue samples was determined with the CIBERSORT algorithm that was based on the LM22 gene signature, respectively. The LM22 gene signature-based CIBERSORT algorithm can highly discriminate sensitively and specifically 22 human immune cell phenotypes (23). Gene expression profile data prepared with standard annotation files were input into the CIBERSORT web portal (http://cibersort.stanford.edu/) to run the algorithm based on LM22 gene signature and 1,000 permutations (**Supplementary Tables S11** and **S12**). Corrplot R package was used for the correlation analysis between immune cells (**Supplementary Figure S1**). LUAD and LUSC tissue samples were divided into high- and low-abundance subgroups (high and low subgroups) per the median value of the proportions of each single-one immune cell, respectively. The Kaplan–Meier method was used for overall survival analysis and compared to the log-rank test, and *p <*0.05 was considered statistically significant. Furthermore, the association between clinical characteristics (pathologic M, N, T, and stage and cancer status) and the proportions of immune cells were analyzed in LUAD and LUSC patients (**Supplementary Figures S2** and **S3**). The distribution of immune cells was analyzed in LUAD and LUSC.

### Calculation of Tumor Mutation Burden

The distribution of TMB was calculated with Maftools R package, which generated the waterfall and interaction of mutation genes, summary of TMB, and TMB score (**Supplementary Tables S13** and **S14**). The LUAD and LUSC tissue samples were divided into high- and low-TMB score subgroups as per the median value of TMB scores, respectively. According to the high- and low-TMB score subgroups, the distribution of immune cells was analyzed in LUAD and LUSC patients (**Supplementary Figure S4**).

### Differentially Expressed Genes Between High- and Low-Immunity Subtypes in LUAD and LUSC

The R package limma package was used to determine the DEGs between the high- and low-abundance groups (Immunity-H and Immunity-L) of immune cell infiltration in LUAD and LUSC patients, with the statistical significance of adjusted P-value <0.05 (**Supplementary Tables S15** and **S16**). limma is mainly used to analyze gene expression data from chip, RNA-seq, and quantitative PCR data. Its main function is to evaluate the differential expression of a multi-factor experiment with a linear model. This algorithm used an empirical Bayesian approach for the estimation of gene expression alterations with moderated *t*-tests. The adjusted *P*-value was calculated with the Benjamini–Hochberg multiple testing correction.

### Functional Characteristic Analysis of Immune-Related DEGs

Gene annotation enrichment analysis of DEGs between immunity_H and immunity_L groups was performed with the clusterProfiler R package, including Gene Ontology (GO) terms (**Supplementary Tables S17** and **S18**) and Kyoto Encyclopedia of Genes and Genomes (KEGG) pathways (**Supplementary Tables S19** and **S20**), with false discovery rate (FDR) <0.05 and statistical significance *P <*0.05. The FDR value was calculated with the Benjamini–Hochberg multiple testing correction procedure, and the enrichment *P*-value was calculated based on 10,000 permutations. The protein–protein interaction (PPI) network was constructed with the STRING database analysis to evaluate interactive associations among all DEGs in LUAD and LUSC patients (**Supplementary Tables S21** and **S22**).

### Construction and Verification of Immune-Related Gene Signature Models With LASSO Regression in LUAD and LUSC

LUAD and LUSC tissue samples were divided into two groups per the median value of DEGs. The Kaplan–Meier method was used for overall survival analysis and compared to the log-rank test, with statistical significance at *p <*0.05 (**Supplementary Figures S5** and **S6**). Furthermore, the overall survival-related DEGs were used for the construction of a lasso regression model, which examined the relationship between immune-related gene signatures and lung cancer risk score. Receiver operating characteristic (ROC) curve and principal component analysis (PCA) were used to test the measurement of classification based on risk sore. The Kaplan–Meier method was used to evaluate the availability of each prognostic signature model. Furthermore, the LUAD and LUSC tissue samples were divided into high- and low-risk score subgroups per the median value of risk scores in LUAD and LUSC (**Supplementary Tables S23** and **S24**). The distribution of immune cells was analyzed in LUAD and LUSC patients between the high- and low-risk score groups. The clinic correlation between the high- and low-risk score groups was analyzed with the pheatmap R package. This risk score assessment nomogram was used to evaluate the prognosis in LUAD and LUSC patients (1-, 3-, and 5-year survival rates). In addition, univariate and multivariate Cox regression models were used to analyze the associations between clinical characteristics (gender, age at initial diagnosis, follow-up, anatomic subdivision, number of pack-year smoked, cancer status, pathologic M/N/T, pathologic stage, radiation therapy, and targeted molecular therapy) and overall survival in LUAD and LUSC patients.

### Verification of Immune-Related Gene Signature Models With Train and Test Cohorts in LUAD and LUSC

The “caret” R package (http://topepo.github.io/caret/index.html) was used to randomly divide the LUAD and LUSC samples into train and test cohorts. The risk scores for each group were calculated according to the gene signature models by LASSO regression (**Supplementary Tables S25**–**S28**). The Kaplan–Meier method was used for overall survival analysis between the high- and low-risk score groups in the train and test cohorts of LUAD and LUSC, respectively.

### Statistical Analysis

For between-group comparisons, for normally distributed variables, the *p*-value was calculated with unpaired Student *t*-tests, and for non-normally distributed variables, the *p*-value was calculated with Mann–Whitney *U*-tests (namely, the Wilcoxon rank–sum test), and statistical significance was set as *p <*0.05. FDR and Benjamini–Hochberg were used for multiple testing to correct the *p*-value in DEGs, GO, and KEGG analyses. The Kaplan–Meier method was used to generate survival curves, and the log–rank (Mantel–Cox) test was used to evaluate the statistical significance of the differences, with statistical significance of *p <*0.05. The hazard ratio was calculated for univariate or multivariate Cox proportional hazard regression models. The predicted response to immunotherapy was statistically analyzed with chi-square test (*X* ^2^) between the high- and low-risk score groups, and statistical significance was set as *p <*0.05.

## Results

### High- and Low-Immunity Subtypes in LUAD and LUSC Identified With ESTIMATE Algorithm

**Figure 2:**
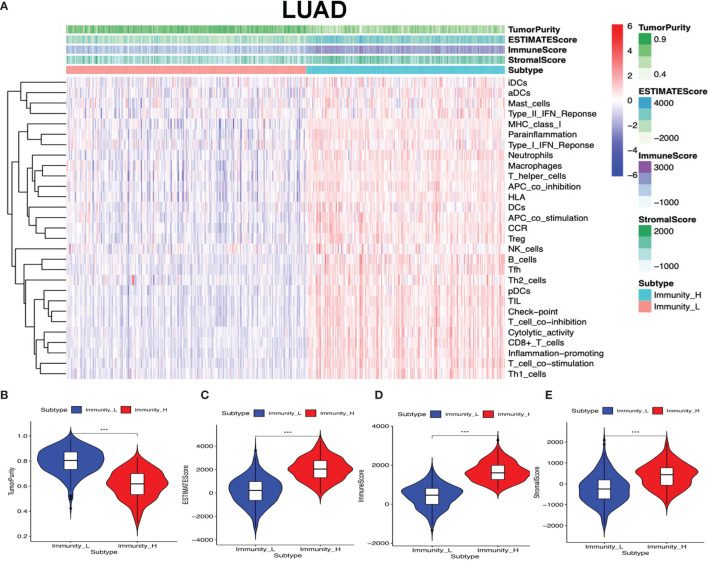
High- and low-immunity abundance groups based on a panel of immune cell infiltrations in lung adenocarcinoma (LUAD), identified with the ESTIMATE algorithm. **(A)** The heat map shows that the LUAD samples were divided into high- and low-immunity abundance groups based on a panel of immune cell infiltrations. **(B)** The difference of tumor purity between the high- and low-immunity abundance groups in LUAD. **(C)** The difference of ESTIMATE score between the high- and low-immunity abundance groups in LUAD. **(D)** The difference of immune score between the high- and low-immunity abundance groups in LUAD. **(E)** The difference of stroma score between the high- and low-immunity abundance groups in LUAD. ***p < 0.001.

**Figure 3:**
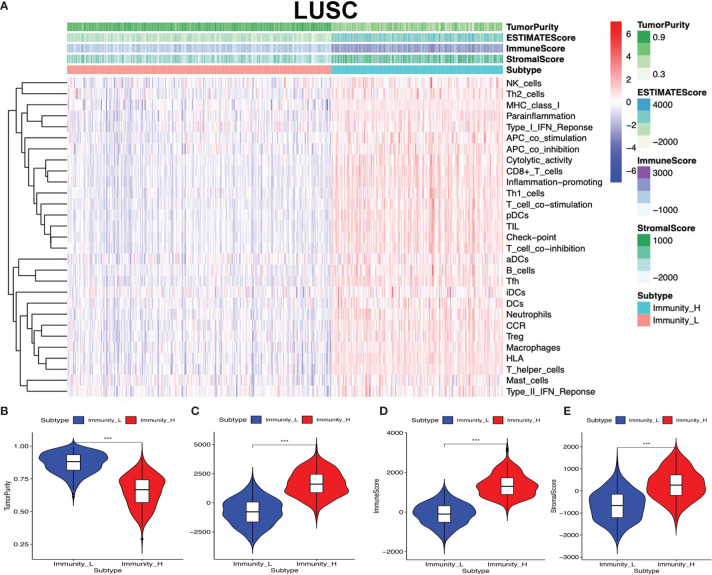
High- and low-immunity abundance groups based on a panel of immune cell infiltrations in lung squamous cell carcinoma (LUSC), identified with the ESTIMATE algorithm. **(A)** The heat map shows that the LUSC samples were divided into high- and low-immunity abundance groups based on a panel of immune cell infiltrations. **(B)** The difference of tumor purity between the high- and low-immunity abundance groups in LUSC. **(C)** The difference of ESTIMATE score between the high- and low-immunity abundance groups in LUSC. **(D)** The difference of immune score between the high- and low-immunity abundance groups in LUSC. **(E)** The difference of stroma score between the high- and low-immunity abundance groups in LUSC. ***p < 0.001.

The lung cancer RNA sequencing data were from 515 LUAD and 502 LUSC patients. The abundance of each TME cell infiltration was calculated by ssGSEA with gene expression data in LUAD and LUSC (**Supplementary Tables S5** and **S6**), including the immune cell information of central memory (Tcm), activated T cells, effector memory (Tem) CD4^+^ and CD8^+^ T cells, T helper 1 (Th1) cells, gamma delta T (Tγδ) cells, Th17 cells, Th2 cells, follicular helper T cells (Tfh), regulatory T cells (Treg), and activated, immature, and memory B cells, as well as innate immunity-related cell types (monocytes, mast cells, macrophages, eosinophils, neutrophils, NK cells, NKT cells, the activated, plasmacytoid, and immature DCs, and MDSCs). According to the data containing immune cell information, the LUAD and LUSC tissue samples were divided into Immunity-H and Immunity-L groups, respectively (Immunity-H: *n* = 233 and Immunity-L: *n* = 282 in LUAD; Immunity-H: *n* = 198 and Immunity-L: *n* = 304 in LUSC) (**Supplementary Tables S7** and **S8**). The ssGSEA-based ESTIMATE algorithm generated stromal score, immune score, and ESTIMATE score for LUAD and LUSC (**Supplementary Tables S9** and **S10**). The high- and low-immunity clusters were significantly positively correlated with stromal score, immune score, and ESTIMATE score and were negatively correlated with tumor purity in LUAD (**Figure 2**) and LUSC (**Figure 3**) (*p* < 0.001).

### The Distribution of Immune Cells Between High- and Low-Immunity Subtypes and Their Correlation With Clinical Features and Survival

**Figure 4:**
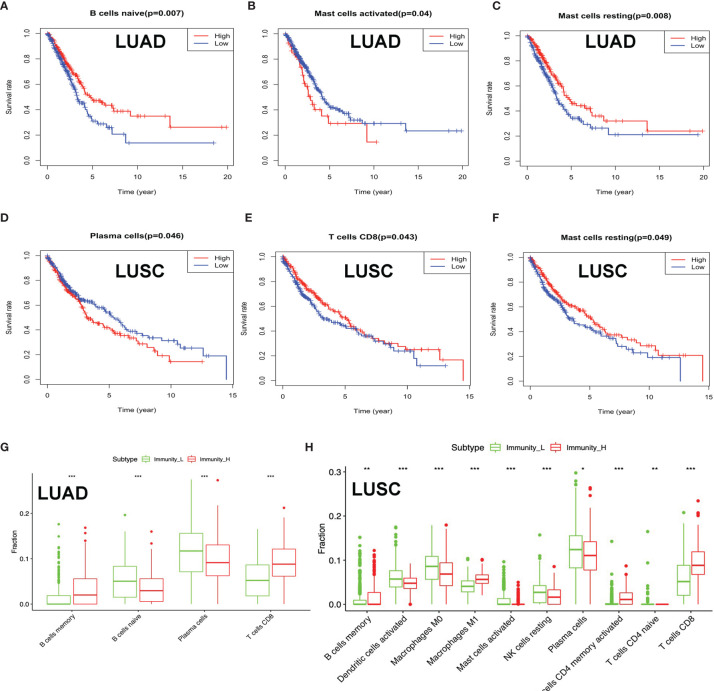
The distribution of immune cells between high- and low-immunity subtypes and their correlation with clinical features and survival. **(A–C)** Kaplan–Meier survival analysis for naïve B cells, activated mast cells, and mast cells resting in lung adenocarcinoma (LUAD). “High” means high-abundance subgroup of single-one immune cell, which is determined by the median values of the abundance of single-one immune cell infiltration. “Low” means low-abundance subgroup of single-one immune cell, which is determined by the median values of the abundance of single-one immune cell infiltration. **(D–F)** Kaplan–Meier survival analysis for plasma cells, T cell CD8, and mast cells resting in lung squamous cell carcinoma (LUSC). “High” means high abundance subgroup of single-one immune cell, which is determined by the median values of the abundance of single-one immune cell infiltration. “Low” means low abundance subgroup of single-one immune cell, which is determined by the median values of the abundance of single-one immune cell infiltration. **(G)** Box plot showing the ratio differences of four immune cells between high- and low-immunity subtypes in LUAD, and Wilcoxon rank sum was used for the significance test. **(H)** Box plot showing the ratio differences of 10 immune cells between high- and low-immunity subtypes in LUSC, and Wilcoxon rank sum was used for the significance test. *P*-value was verified by log-rank test. *p < 0.05, **p < 0.01, and ***p < 0.001.

Clinical data were obtained from TCGA database, including gender (male and female), age (from 33 to 90 years), primary disease (LUAD and LUSC), anatomic subdivision (L-lower, L- middle, L-upper, R-lower, R-middle, and R-upper), follow-up outcome (partial remission/response, complete remission/response, progressive disease, and stable disease), number of pack-year smoked (packs from 0.15 to 240), pathologic stage (stages I, II, III, and IV), pathologic N (tumor lymph node metastasis, including N0, N1, N2, and NX), pathologic M (tumor metastasis, including M0, M1, and MX), pathologic T (tumor size, including T1, T2, T3, T4, and TX), person neoplasm cancer status (tumor or tumor-free), radiation therapy (no or yes), targeted molecular therapy (no or yes), and status (alive or dead) (**Supplementary Tables S1** and **S2**). The tumor immune cell infiltration of the 515 LUAD (**Supplementary Figure 1A** and **Supplementary Table S11**) and 502 LUSC (**Supplementary Figure S1B** and **Supplementary Table S12**) samples was summarized. The between-immune-cell correlation analysis was carried out with Corrplot R to show (i) the interaction between tumor immune cells in LUAD—for example, T cells CD4 memory resting and CD8 T cell, macrophages M1 and dendritic cells activated, T cell follicular helper and T cell CD4 memory resting, macrophages M0 and dendritic cells resting, B cells naïve and B cells memory, macrophages M2 and plasma cell, and NK resting cell and NK activated cell (**Supplementary Figure S1C**) and (ii) the interaction between tumor immune cells in LUSC—for example, mast cells resting and mast cell activated, NK cell activated and NK cell resting, T cells CD4 memory resting and T cell CD8, B cells naïve and B cells memory, T cells follicular helper and T cells CD4 memory resting, macrophages M0 and dendritic cells resting, and macrophages M1 and dendritic cells activated (**Supplementary Figure S1D**). B cells naïve, mast cell activated, and mast cells resting were significantly related to LUAD survival (**Figures 4A–C**). Plasma cell, T cell CD8, and mast cells resting were significantly related to LUSC survival (**Figures 4D–F**). In terms of clinical characteristics in LUAD, dendritic cells activated, T cell follicular helper, neutrophils, and mast cells resting were significantly distributed differentially in different pathologic T; neutrophils, macrophages M0, T cells CD4 memory resting, and activated dendritic cells were significantly distributed differentially in different pathologic N; macrophages M0, B cells memory, NK cells activated, T cells CD4 memory resting, and eosinophils were significantly distributed differentially in different pathologic M; neutrophils, macrophages M0, NK cells activated, and mast cells resting were significantly distributed differentially in different pathologic stage, and dendritic cells activated and dendritic cells resting were significantly distributed differentially in different cancer status (**Supplementary Figure S2**). In terms of clinical characteristics in LUSC, T cells CD4 memory resting, mast cells activated, neutrophils, macrophages M0, and T cells follicular helper were significantly distributed differentially in different pathologic T; T cells CD4 memory resting and macrophages M1 were significantly distributed differentially in different pathologic N; monocytes, T cells regulatory (Tregs), T cells CD4 memory resting, and neutrophils were significantly distributed differentially in different pathologic M; and monocytes and T cells regulatory (Tregs) were significantly distributed differentially in different pathologic stage (**Supplementary Figure S3**). The distribution of immune cells was significantly changed between Immunity-H and Immunity-L groups in LUAD, including B cells memory, plasma cells, B cells naïve, and T cells CD8 (**Figure 4G**). The distribution of immune cells was significantly changed between Immunity-H and Immunity-L groups in LUSC, including B cells memory, macrophages M1, macrophages M0, dendritic cells activated, NK cells resting, mast cells activated, plasma cells, T cells CD4 naïve, T cells CD4 memory activated, and T cells CD8 (**Figure 4H**).

### Tumor Mutation Information and the Distribution of Immune Cells in Different TMB Subtypes of Lung Cancer

To investigate the relationship of immune status and mutation status in lung cancer, the distribution of tumor mutation was plotted in LUAD and LUSC (**Supplementary Tables S3** and **S4**). The mutations of the top 30 genes were plotted in LUAD, including TP53, MUC16, TNN, RYR2, CSMD3, LRP1B, ZFHX4, USH2A, KRAS, XIRP2, SPTA1, FLG, COL11A1, NAV3, ZNF536, FAT3, ANK2, CSMD1, KEAP1, MUC17, PCDH15, APOB, ADAMTTS12, ADGRG4, PCLO, TNR, DNAH9, RP1L1, NPAP1, and PAPPA2 (**Supplementary Figure S4A**). The significant co-occurrence of gene mutations was plotted in LUAD, including TP53 and TNN, TP53 and RYR2, MUC16 and TNN, RYR2 and TNN, CSMD3 and TNN, LRP1B and MUC16, APOB and XIRP2, FLG and USH2A, FLG and XIRP2, KRAS and TP53, and KRAS and MUC16 (**Supplementary Figure S4B**). Moreover, the mutations of the top 30 genes were plotted in LUSC, including TP53, TTN, CSMD3, MUC16, RYR2, LRP1B, USH2A, SYNE1, ZFHX4, KMT2D, FAM135B, XIRP2, CDH10, SPTA1, NAV3, PCDH15, PAPPA2, RYR3, DNAH5, PKHD1, DNAH8, PKHD1L1, HCN1, COL11A1, DNAH9, ERICH3, DAMTS12, FLG, MUC17, and SI (**Supplementary Figure S4C**). The significant co-occurrence of gene mutations was plotted in LUSC, including SI and MUC16, SI and ZFHX4, PAPPA2 and TNN, PAPPA2 and RYR2, RYR3 and MUC16, NAV3 and CSMD3, and LRP1B and NAV3 (**Supplementary Figure S4D**). TP53 mutation was ranked top one in both LUAD and LUSC, and TP53 mutation would increase the resistance of some drugs, including nutlin-3a (-), oxaliplatin, ERK_2440, ERK_6604, dactinomycin, epirubicin, and SCH772984 (**Supplementary Figure S4E**). The distribution of TP53 mutation in protein domains was plotted in LUAD (**Supplementary Figure S4F**) and LUSC (**Supplementary Figure S4G**), respectively; its main mutations were located in P53 domain (**Supplementary Figures S4F, G**). Furthermore, the distribution of immune cells was significantly changed between the high- and low-TMB-score groups in LUAD (**Supplementary Table S13**), including B cells naïve, dendritic cells activated, dendritic cells resting, macrophages M1, macrophages M0, mast cells resting, plasma cells, NK cells activated, T cells CD4 memory resting, T cells CD4 memory activated, T cells follicular helper, and T cells CD8 (**Supplementary Figure S4H**). The distribution of immune cells was significantly changed between the high- and low-TMB-score groups in LUSC (**Supplementary Table S14**), including macrophages M1, dendritic cells resting, NK cells activated, T cells CD8, T cells CD4 memory resting, and T cells follicular helper (**Supplementary Figure S4I**).

### DEGs Between High- and Low-Immunity Subtypes, Immune-Related Genes, and Immune-Related Pathways in Lung Cancer

**Figure 5:**
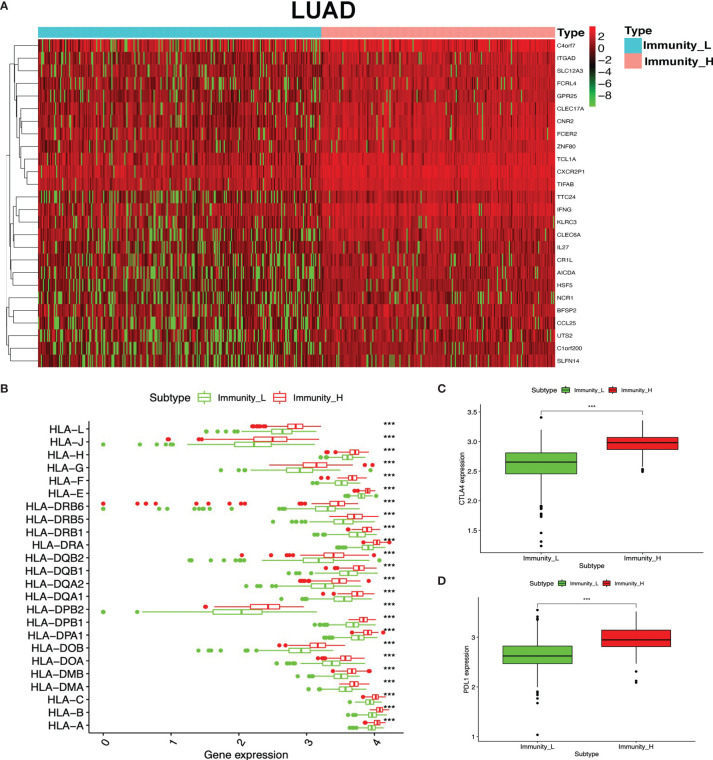
Differentially expressed genes (DEGs) between immunity-H and immunity-L subtypes in lung adenocarcinoma (LUAD). **(A)** The heat map shows DEGs between high- and low-immunity subtypes in LUAD. **(B)** Box plot showing the different DEGs of the HLA family between high- and low-immunity subtypes in LUAD. **(C)** Box plot showing the differential expression of CTLA-4 (CD152) between high- and low-immunity subtypes in LUAD. **(D)** Box plot showing the differential expression of PD-L1 (CD274) between high- and low-immunity subtypes in LUAD. ***p <0.001.

**Figure 6:**
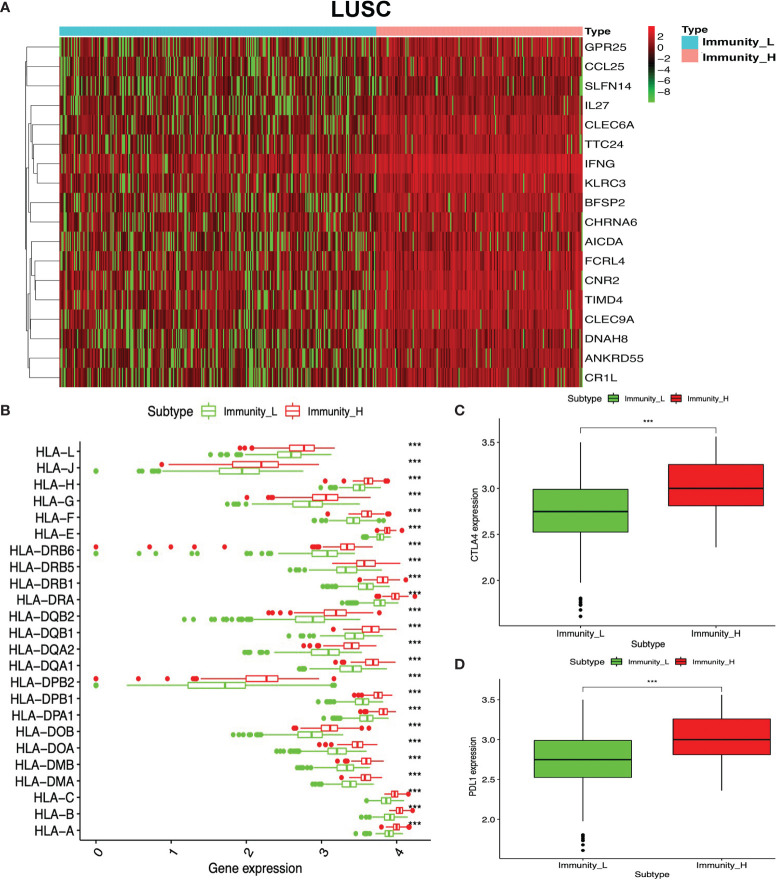
Differentially expressed genes (DEGs) between immunity-H and immunity-L subtypes in lung squamous cell carcinoma (LUSC). **(A)** The heat map shows DEGs between high- and low-immunity subtypes in LUSC. **(B)** Box plot showing the different DEGs of HLA family between high- and low-immunity subtypes in LUSC. **(C)** Box plot showing the differential expression of CTLA-4 (CD152) between high- and low-immunity subtypes in LUSC. **(D)** Box plot showing the differential expression of PD-L1 (CD274) between high- and low-immunity subtypes in LUSC. ***p <0.001.

To identify the underlying biological characteristics in high- and low-immunity subtypes of lung cancers, 20,530 genes were analyzed with limma package, and 112 DEGs were identified between the high- and low-immunity subtypes in LUAD (**Figure 5A** and **Supplementary Table S15**), while 231 DEGs were identified between the high- and low-immunity subtypes in LUSC (**Figure 6A** and **Supplementary Table S16**). Those DEGs contained many hot immune-related genes—for example, HLA family in LUAD (**Figure 5B**) and LUSC (**Figure 6B**) and immune checkpoints CTLA4 in LUAD (**Figure 5C**) and LUSC (**Figure 6C**) and PDL1 in LUAD (**Figure 5D**) and LUSC (**Figure 6D**).

**Figure 7:**
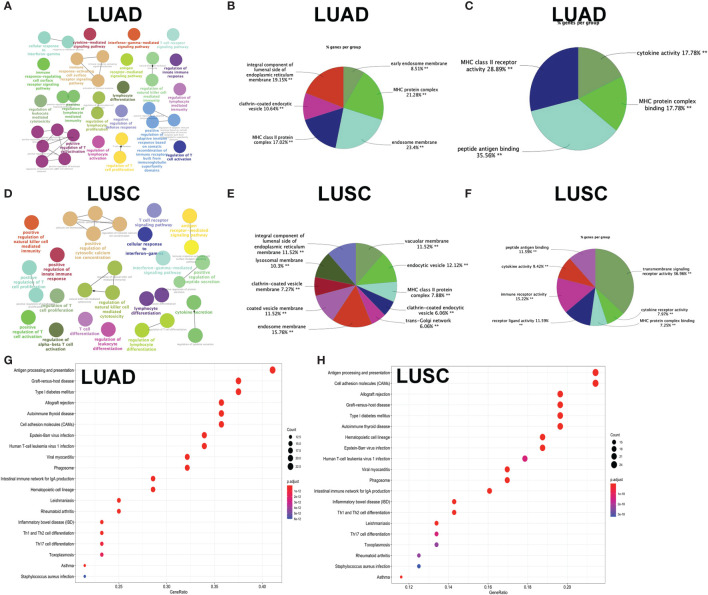
Functional characteristics and pathway enrichment analysis of immune-related differentially expressed genes (DEGs) in lung adenocarcinoma (LUAD) and lung squamous cell carcinoma (LUSC). **(A)** The biological process analysis of DEGs between high- and low-immunity subtypes in LUAD. **(B)** The cellular component analysis of DEGs between high- and low-immunity subtypes in LUAD. **(C)** The molecular function analysis of DEGs between high- and low-immunity subtypes in LUAD. **(D)** The biological process analysis of DEGs between high- and low-immunity subtypes in LUSC. **(E)** The cellular component analysis of DEGs between high- and low-immunity subtypes in LUSC. **(F)** The molecular function analysis of DEGs between high- and low-immunity in LUSC. **(G)** Kyoto Encyclopedia of Genes and Genomes (KEGG) pathway enrichment analysis of DEGs between high- and low-immunity subtypes in LUAD. **(H)** KEGG pathway enrichment analysis of DEGs between high- and low-immunity subtypes in LUSC. Only gene sets with nominal *p <*0.05 and false discovery rate (FDR) *q <*0.05 were considered as statistically significant. Benjamini–Hochberg multiple testing procedure was used to adjust the *p*-value for FDR control. The greater node size showed the less *P*-value and more significant enrichment. The same color indicated the same function group. Among the groups, we chose a representative of the most significant term and the lag highlighted. The color depth represented enriched adjusted *p*-value.

GO enrichment analyses were used to identify the functional characteristics of immune-related DEGs in LUAD and LUSC. A total of 70 statistically significant GO terms were obtained in LUAD (**Supplementary Table S17**), including 34 GO biological processes (**Figure 7A**), 30 cellular components (**Figure 7B**), and six molecular functions (**Figure 7C**). A total of 62 statistically significant GO terms were obtained in LUSC (**Supplementary Table S18**), including 29 biological processes (**Figure 7D**), 24 cellular components (**Figure 7E**), and nine molecular functions (**Figure 7F**). These GO enrichment results demonstrated that the immune-related DEGs were closely associated with immune processes—for example, regulation of leukocyte-mediated cytotoxicity, immune response-regulating cell surface receptor signaling pathway, regulation of T cell activation, cytokine-mediated signaling pathway, lymphocyte differentiation, antigen receptor-mediated signaling pathway, natural killer cell-mediated immunity, T cell proliferation, regulation of adaptive immune response based on somatic, positive regulation of leukocyte cell–cell adhesion, major histocompatibility complex (MHC) protein complex, MHC class II protein complex, cytokine activity, immune receptor activity, peptide antigen binding, and MHC class II receptor activity.

KEGG enrichment analyses were used to identify immune-related pathways based on DEGs between the high- and low-immunity subtypes in LUAD and LUSC. A total of 34 significant KEGG pathways was obtained in LUAD (**Figure 7G** and **Supplementary Table S19**) and 35 significant KEGG pathways in LUSC (**Figure 7H** and **Supplementary Table S20**). The KEGG pathway enrichment results also demonstrated that immune-related DEGs were closely associated with immune processes—for example, Th1 and Th2 cell differentiation, antigen processing and presentation, Th17 cell differentiation, natural killer cell-mediated cytotoxicity, cytokine–cytokine receptor interaction, primary immunodeficiency, T cell receptor signaling pathway, viral protein interaction with cytokine and cytokine receptor, and intestinal immune network for IgA production.

**Figure 8:**
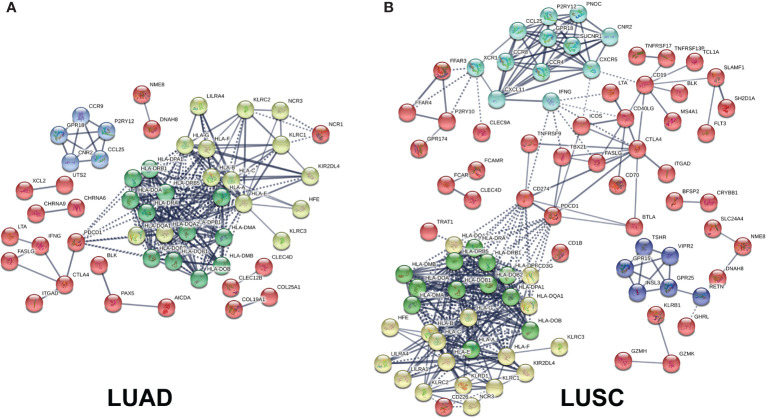
Protein–protein interaction (PPI) networks based on immune-related differentially expressed genes (DEGs) in lung adenocarcinoma (LUAD) and lung squamous cell carcinoma (LUSC). **(A)** PPI network based on immune-related DEGs in LUAD. **(B)** PPI network based on immune-related DEGs in LUSC.

Those immune-related DEGs were used to construct a PPI network for LUAD (**Figure 8A** and **Supplementary Table S21**) and LUSC (**Figure 8B** and **Supplementary Table S22**), respectively. The entire PPI network was clustered into different hub modules. Four hub modules were identified for LUAD, including HLA family (HLA-J, HLA-L, HLA-H, HLA-F, HLA-G, HLA-DRB6, HLA-E, HLA-DRB1, HLA-DRB5, HLA-DQB2, HLA-DRA, HLA-DQB1, HLA-DQA1, HLA-DQA2, HLA-DPB1, HLA-DPB2, HLA-DOB, HLA-DPA1, HLA-DOA, HLA-DMA, HLA-DMB, HLA-B, HLA-C, and HLA-A) and G-protein interaction proteins (CCR9, CCL25, P2RY12, CNR2, and GPR18). Five hub modules were identified for LUSC, including HLA family (HLA-L, HLA-H, HLA-J, HLA-F, HLA-G, HLA-E, HLA-DRB5, HLA-DRB6, HLA-DRA, HLA-DRB1, HLA-DQB1, HLA-DQB2, HLA-DQA2, HLA-DPB2, HLA-DQA1, HLA-DPB1, HLA-DPA1, HLA-DOB, HLA-DMB, HLA-DOA, HLA-C, HLA-DMA, HLA-B, and HLA) and G-protein coupled receptors (CCR8, CCL25, CXCL11, CXCR5, XCR1, IFNG, CCR4, GPR18, P2RY12, PNOC, SUCNR1, and CNR2).

### Optimization of the Immune-Based Prognostic Models for LUAD and LUSC

**Figure 9:**
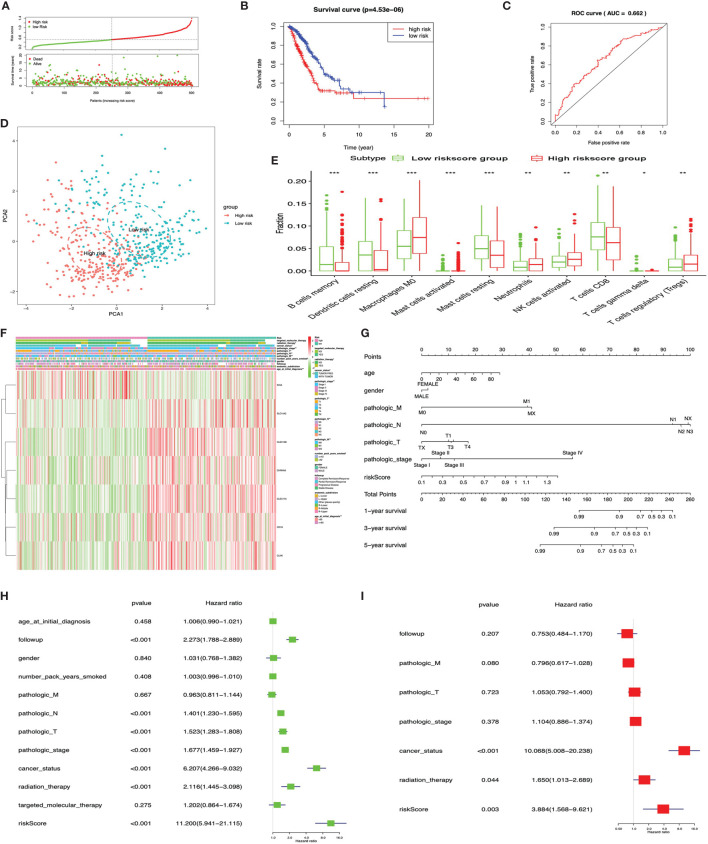
LASSO regression identified the prognostic model in lung adenocarcinoma (LUAD). **(A)** Risk plot between the high- and low-risk-score groups. **(B)** Overall survival analysis between the high- and low-risk-score groups. **(C)** Receiver operating characteristic curve based on risk score in LUAD. **(D)** Principal component analysis for the risk scores revealed two completely disjoint populations, suggesting that there existed extensive differences in the landscape of risk scores between the high- and low-risk-score samples. Blue means low-risk-score samples; red means high-risk-score samples. **(E)** Box plot showing the ratio differences of 10 immune cells between the high- and low-risk-score groups in LUAD, and Wilcoxon rank–sum was used for the significance test. **(F)** Heat map of the clinical correlation between the high- and low-risk-score groups in LUAD. **(G)** The risk score assessment nomogram to evaluate prognosis in LUAD (1-, 3-, and 5-year survival rates). **(H)** The univariate Cox regression analysis of risk factors in LUAD. **(I)** The multivariate Cox regression analysis of risk factors in LUAD. *p < 0.05, **p < 0.01, and ***p < 0.001.

The KM plot analysis found that 51 out of 112 immune-related DEGs were significantly related to LUAD overall survival (*p* < 0.05) (**Supplementary Figure S5**). Furthermore, the seven-immune-related-gene-signature model (CD1B, CHRNA6, CLEC12B, CLEC17A, CLNK, INHA, and SLC14A2) was identified with lasso regression to improve the predicted accuracy for overall survival in LUAD when log (lambda) was between -3 and -4 (**Supplementary Figures S7A, B**). Based on this seven-immune-related-gene-signature model, the LUAD tissue samples were divided into high- and low-risk-score groups according to the mean value of risk scores (**Supplementary Table S23**). Additionally, overall survival had a statistical significance between the high- and low-risk-score groups (**Figures 9A, B** **)**. The ROC curve showed area under the curve (AUC) = 0.662 (**Figure 9C**), and all LUAD samples can be well divided into high-risk and low-risk groups according to risk score based on verification of PCA (**Figure 9D**). The distribution of immune cells was significantly changed between high- and low-risk-score groups in LUAD, including B cells memory, dendritic cells resting, mast cells activated, mast cells resting, macrophages M0, neutrophils, NK cells activated, T cells CD8, T cell gamma delta, and Tregs (**Figure 9E**). The heat map showed that the risk group had a significant association with clinical features, including age at initial diagnosis, the number of pack-year smoked, pathologic N, pathologic M, pathologic T, cancer status, pathologic stage, and radiation therapy (**Figure 9F**). Furthermore, the nomogram was made to provide a more simple and convenient method for estimating the patient survival rate according to basic clinical characteristics and risk score (**Figure 9G**). The seven-immune-related-gene-signature was consistent with single-factor Cox regression analysis of gene. The univariate Cox regression analysis found that follow-up, pathologic N, pathologic T, pathologic stage, cancer status, radiation therapy, and risk score were significantly correlated with overall survival (**Figure 9H**). The multivariate Cox regression analysis found that cancer status, radiation therapy, and risk score possibly acted as an independent risk factor in LUAD (**Figure 9I**).

**Figure 10:**
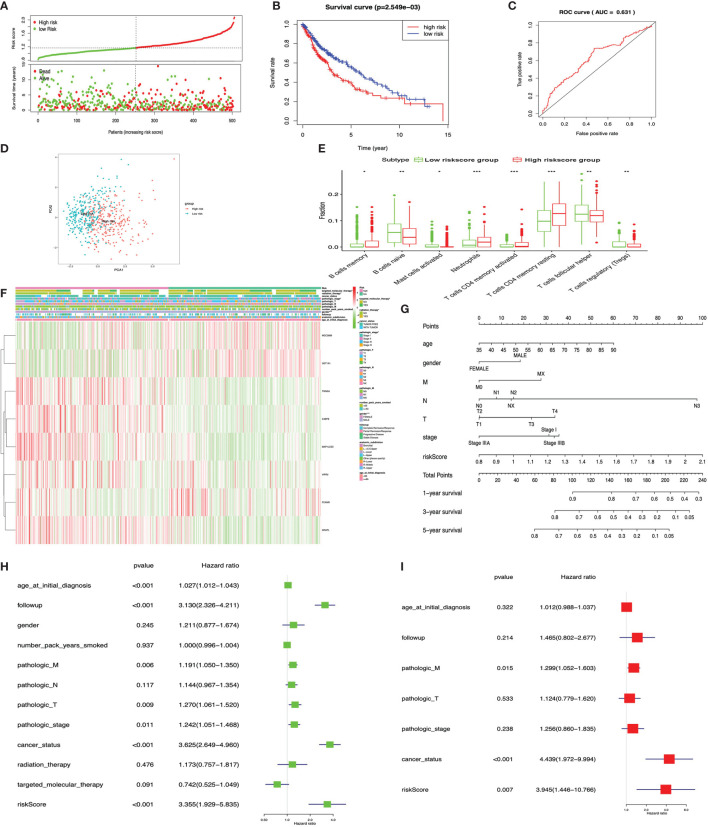
LASSO regression identified the prognostic model in lung squamous cell carcinoma (LUSC). **(A)** Risk plot between the high- and low-risk-score groups. **(B)** Overall survival analysis between the high- and low-risk-score groups. **(C)** Receiver operating characteristic curve based on risk score in LUSC. **(D)** Principal component analysis for the risk scores revealed two populations. Blue means low-risk-score samples; red means high-risk-score samples. **(E)** Box plot showing the ratio differences of eight immune cells between the high- and low-risk-score groups in LUSC, and Wilcoxon rank–sum was used for the significance test. **(F)** The heat map of clinical correlation between the high- and low-risk-score groups. **(G)** The risk score assessment nomogram to evaluate prognosis in LUSC (1-, 3-, and 5-year survival rates). **(H)** The univariate Cox regression analysis of risk factors in LUSC. **(I)** The multivariate Cox regression analysis of risk factors in LUSC. *p < 0.05, **p < 0.01, and ***p < 0.001.

The KM plot analysis found that 12 out of 231 immune-related DEGs were significantly related to LUSC overall survival (*p* < 0.05), including C4BPB, CD300E, FCAMR, GRAPL, LCNL1, MAP1LC3C, MGC2889, NLRP12, STAP1, TRIM55, UGT1A1, and VIPR2 (**Supplementary Figure S6**). Furthermore, the eight-immune-related-gene-signature model (C4BPB, FCAMR, GRAPL, MAP1LC3C, MGC2889, TRIM55, UGT1A1, and VIPR2) was identified with lasso regression to improve the predictive accuracy for overall survival in LUSC when log (lambda) was between -3 and -4 (**Supplementary Figures 7C, D** **)**. Based on the eight-immune-related-gene-signature model, the LUSC tissue samples were divided into high- and low-risk-score groups according to the mean value of risk scores (**Supplementary Table S24**). Additionally, overall survival had a statistical significance between the high- and low-risk-score groups (**Figures 10A, B** **)**. The ROC curve showed AUC = 0.631 (**Figure 10C**), and all LUSC tissue samples can be well divided into high-risk and low-risk groups according to risk scores based on verification of PCA (**Figure 10D**). The distribution of immune cells was significantly changed between the high- and low-risk-score groups in LUSC, including B cells memory, B cell naïve, mast cells activated, T cell CD4 memory activated, neutrophils, T cell follicular helper, T cell CD4 memory resting, and Tregs (**Figure 10E**). The heat map showed that the risk group had a significant association with clinical features, including gender, targeted therapy, radiation therapy, and pathologic stage (**Figure 10F**). Furthermore, the nomogram was made to provide a more simple and convenient method for estimating the patient survival rate according to basic clinical characteristics and risk score (**Figure 10G**). The eight-immune-related-gene-signature was consistent with single-factor Cox regression analysis of gene. The univariate Cox regression analysis found that follow-up, age at initial diagnosis, pathologic T, pathologic M, pathologic stage, cancer status, and risk score were significantly correlated with overall survival (**Figure 10H**). The multivariate Cox regression analysis found that cancer status, pathologic M, and risk score possibly acted as an independent risk factor in LUSC (**Figure 10I**).

### The Verification of Immune-Related Gene Signature Models With Train and Test Cohorts Provided Consistency

**Figure 11:**
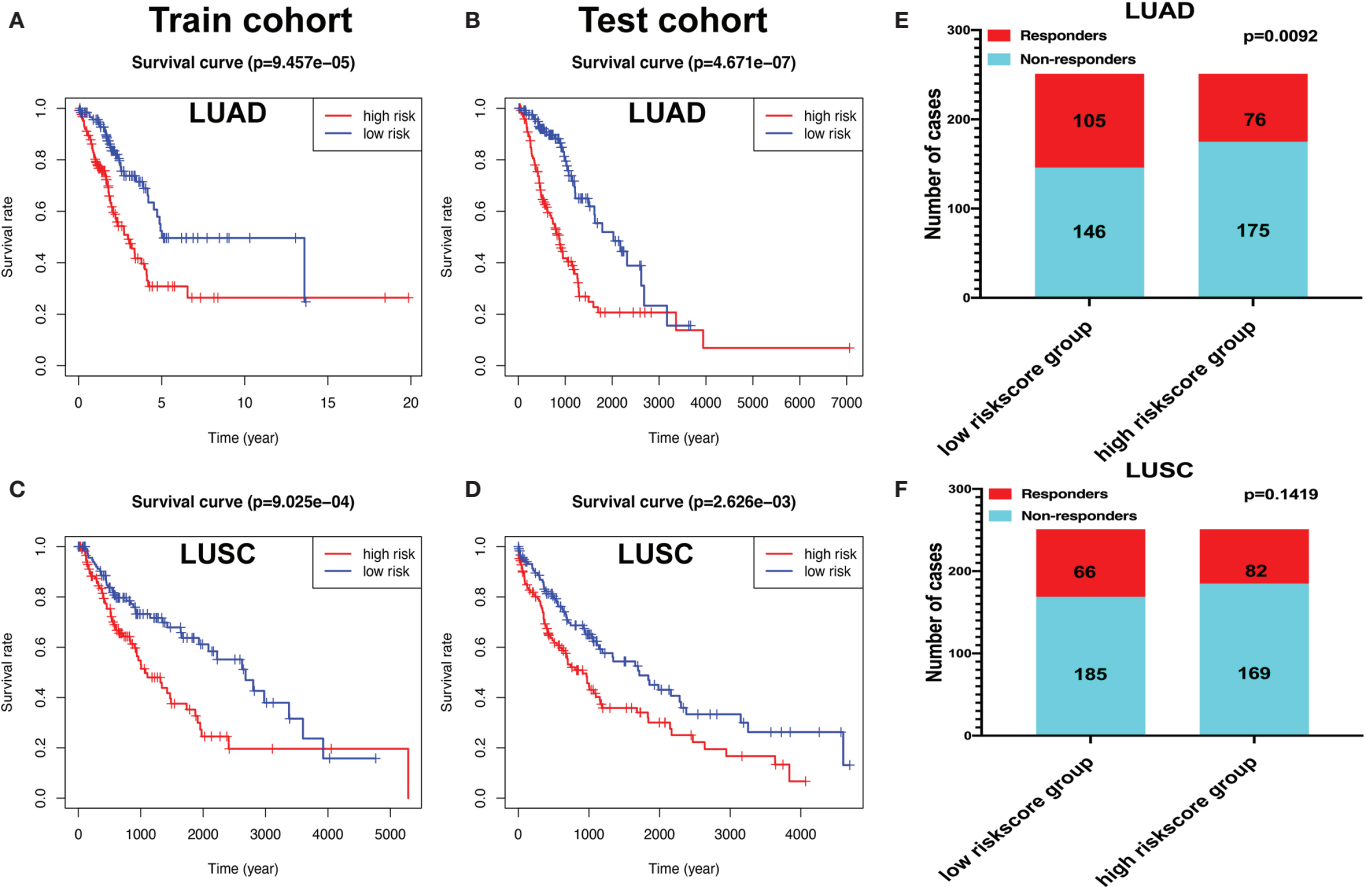
The Kaplan–Meier (KM) curve of train and test cohorts in lung adenocarcinoma (LUAD) and lung squamous cell carcinoma (LUSC). **(A)** The KM curve of train cohort in LUAD between the high- and low-risk-score groups. **(B)** The KM curve of test cohort in LUAD between the high- and low-risk-score groups. **(C)** The KM curve of train cohort in LUSC between the high- and low-risk-score groups. **(D)** The KM curve of test cohort in LUSC between the high- and low-risk-score groups. **(E)** Immune response difference between the high- and low-risk-score groups based on Tumor Immune Dysfunction and Exclusion scores in LUAD. **(F)** Immune response difference between the high- and low-risk-score groups based on TIDE scores in LUSC.

The “caret” R package was used to randomly divide the LUAD samples into train (*n* = 252) and test cohorts (*n* = 250). The risk scores for each group were calculated according to the seven-immune-related-gene-signature model (CD1B, CHRNA6, CLEC12B, CLEC17A, CLNK, INHA, and SLC14A2) (**Supplementary Tables S25** and **S26**). The Kaplan–Meier method was used for overall survival analysis between the high- and low-risk-score groups both in the train and test cohorts in LUAD, respectively. The KM curves showed that the risk scores in the train and test cohorts were significantly associated with LUAD overall survival (**Figures 11A, B** **)**. The risk scores for each group were calculated according to the eight-immune-related-gene-signature (C4BPB, FCAMR, GRAPL, MAP1LC3C, MGC2889, TRIM55, UGT1A1, and VIPR2) model (**Supplementary Tables S27** and **S28**). The Kaplan–Meier method was used for overall survival analysis between the high- and low-risk-score groups both in the train and test cohorts in LUSC, respectively. The KM curves showed that the risk score in the train and test cohorts were significantly associated with LUSC overall survival (**Figures 11C, D** **)**.

### Immunotherapy Response in LUAD and LUSC Patients Based on a TIDE Algorithm

The likelihood of an immunotherapy response in LUAD (**Supplementary Table S29**) and LUSC (**Supplementary Table S30**) patients based on a TIDE algorithm was predicted. **Figure 11E** shows that the low-risk group had a lower TIDE prediction score in LUAD (*P* = 0.0092). The response rate to immunotherapy in LUSC has no statistical significance between the high- and low-risk-score groups (**Figure 11F**). These results provided further evidence that patients in the low-risk group have better prognosis and may have more potential for immunotherapy.

## Discussion

Lung cancer is a common malignant epithelial tumor, which ranks at the top worldwide in terms of mortality for men and women (24). LUAD is the most common subtype of lung cancer among women and non-smokers; by contrast, smoking increased the risk of LUSC in men (25). Generally speaking, LUAD grows slowly than LUSC at the same pathologic stage, but metastasis is always screened at the early stage in LUAD (26). Pan-cancer studies suggested that the gene mutation and molecular mechanisms could be highly heterogeneous between different subtypes of lung cancer, even in LUAD itself (27). Consequently, the therapies between LUAD and LUSC are often different (25). The first-line chemotherapy for LUAD was pemetrexed, in combination with two platinum drugs. The mutation-targeted treatment plan for LUAD was relatively complete and sensitive in patients. EGFR and ALK are the most important inhibitors in LUAD—for example, afatinib (Gilotrif), erlotinib (Tarceva), osimertinib (Tagrisso), dacomitinib (Vizimpro), and gefitinib (Iressa) for EGFR and ceritinib (Zykadia), crizotinib (Xalkori), lorlatinib (Lorbrena), brigatinib (Alunbrig), and alectinib (Alecensa) for ALK (28). However, the therapies for LUAD are often ineffective for LUSC. Previous studies found that the immune system was involved in controlling tumorigenesis and the progression of lung cancer (29). Immunotherapy was shown to be effective in both LUAD and LUSC, and three PD-1/PD-L1 inhibitors (Opdivo, Keytruda, and Tecentriq) approved for lung cancer have obtained exciting results (30). Two phase III clinical trials (CheckMate057 and CheckMate017) in NSCLC patients showed that nivolumab continued to obtain a long-term overall survival (13.4 *vs.* 2.6%) rate and progression-free survival (8 *vs.* 0%) benefit compared to chemotherapy with docetaxel (31). The results of the clinical trial KEYNOTE-010 confirmed that pembrolizumab monotherapy was a safe and effective agent for NSCLC patients, with a median overall survival of 12.7 months for pembrolizumab *versus* 8.5 months for docetaxel (32). Although various methods have been studied to predict the clinical responses and outcomes of lung cancers with immunotherapy based on immune-related gene profiling (33), the reliable and effective immune cells, immune-related genes, pathways, and immune-related gene signature during the progression process of LUAD and LUSC were still lacking. Here we systematically studied the different genes between high- and low-immunity clusters in LUAD and LUSC, respectively, and constructed immune-related gene signatures for LUAD and LUSC.

In terms of immune cells, the present study found that the distribution of immune cells was significantly changed between the high- and low-immunity subtypes in LUAD, including B cells memory, B cells naïve, T cells CD8, and plasma cells, and in LUSC, including dendritic cells activated, B cells memory, macrophages M1, macrophages M0, NK cells resting, mast cells activated, T cells CD4 memory activated, plasma cells, T cells CD8, and T cells CD4 naïve. The different distribution of immune cells was closely associated with immunotherapy strategies for patients with lung cancers—for example, one study showed that stage I LUAD lesions already contained significantly altered T cell and NK cell compartments; in addition, the altered tumor-infiltrating myeloid cell subsets likely compromised the anti-tumor T cell immunity (34). Tumor-infiltrating B lymphocytes existed in all stages of cancer and played an important role to shape tumor development. Recent studies have demonstrated that, in more than 35% of lung cancers, proliferating B cells can be observed, and their proportion differs between stage and histological subtypes. B cells participate in humoral and cellular immunity and might exert protumor functions or antitumor activity (35). The multifaceted effects of cancer-associated T cells also have been broadly studied in lung cancer. CD4^+^ Th1 cells, activated CD8^+^ T cells, and γδ-T cells were proved to be related to favorable prognosis of lung cancer, whereas Th2, Th17, and Foxp3^+^ Treg cells were proved to be related to an unfavorable prognosis of lung cancer (36). The correlations between the different immune cell types in the tumor microenvironment showed that immune cells may influence each other and have some cross-talking—for example, there is growing evidence for alternative CD8(+) T cell fates influencing CD4(+) T-cell-mediated responses (37). Macrophages were classified as inflammatory (M1) and alternatively activated (M2) macrophage types. One study showed that conditioned media from acute lymphoblastic leukemia cells promote the generation of dendritic cells with immunosuppressive features and skew M1-like macrophage polarization towards a less pro-inflammatory phenotype (38). In some degree, the correlations of immune cells in our study provided the cross-talking of TME. The present study further found that the distribution of immune cells was significantly changed between the high- and low-risk-score groups in LUAD, including dendritic cells resting, B cells memory, mast cells activated, macrophages M0, neutrophils, mast cells resting, T cells CD8, NK cells activated, T cell gamma delta, and Tregs. The distribution of immune cells was significantly changed between the high- and low-risk score groups in LUSC, including B cell naïve, B cells memory, neutrophils, mast cells activated, T cell CD4 memory resting, T cell CD4 memory activated, T cell follicular helper, and Tregs. The high- and low-risk-score groups were based on DEGs between high- and low-immunity subtypes, which might lead tumor immunotherapy to personalized and precision medicine.

In terms of the tumor mutation burden, the response to immunotherapy of a variety of cancers can be predicted by defining the threshold of tumor mutation burden. TMB, as a new biomarker, has attracted extensive attention recently and is an index to measure the number of tumor mutations (39). The present study also found that the distribution of immune cells was significantly changed between the high- and low-TMB-score groups in LUAD, including dendritic cells activated, B cells naïve, macrophages M0, dendritic cells resting, mast cells resting, macrophages M1, T cells CD4 memory activated, NK cells activated, plasma cells, T cells CD8, T cells CD4 memory resting, and T cells follicular helper, and in LUSC, including macrophages M1, dendritic cells resting, T cells CD4 memory resting, NK cells activated, T cells follicular helper, and T cells CD8. TMB, as a potential biomarker in oncology, could be applied in the clinic. Cancer-specific TMB thresholds for the effective prediction of treatment response in multiple cancers should be well established, which potentially could help lead immuno-oncology to precision medicine (36). It was hypothesized that tumors with higher TMB have more neoantigens that can be well recognized by the immune system in response to checkpoint inhibition (40). In a separate study, the result of whole-exome sequencing showed that NSCLC patients who received pembrolizumab had improved durable clinical benefit and overall response rates with high somatic nonsynonymous mutation burden (41). TMB was examined in the Checkmate 026 clinical study of metastatic NSCLC patients who received nivolumab or platinum doublet chemotherapy as first-line therapy. Patients with a high TMB obtained a higher response rate when compared with nivolumab chemotherapy (47 *vs.* 28%) and improved progression-free survival (9.7 *vs.* 5.8 months). In addition, patients with high TMB and high PD-L1 obtained the best outcomes, and those who had low TMB and low PD-L1 did the worst (42). The further study showed that TMB and specific immune cells were associated with responses, and T follicular helper cells and B cells mediated the responses to checkpoint inhibitors in high mutation burden in mouse tumor models (43). The systematic study on immune cells in LUAD and LUSC in terms of immunity group, risk score group, and TMB group well reflected the tumor immune microenvironment of LUAD and LUSD.

In terms of immune-related pathways, the present study found that immune-related DEGs were enriched in multiple immune-related pathways, including Th1 and Th2 cell differentiation, antigen processing and presentation, cytokine–cytokine receptor interaction, Th17 cell differentiation, viral protein interaction with cytokine and cytokine receptor, natural killer cell mediated cytotoxicity, T cell receptor signaling pathway, primary immunodeficiency, and intestinal immune network for IgA production. Some of the identified pathways have been reported to be closely related with lung cancer in a previous study (44)—for example, the defective HLA class I antigen processing and presentation machinery components played a role in the acquired resistance to PD-1 or PD-L1 antagonistic antibodies in immune checkpoint inhibitor-resistant lung cancer samples (45). Th1 and Th2 cell differentiation pathways also play a significant role in inflammation and cancer. Some researchers were enthusiastic about the regulator of Th1 and Th2, which might be a therapeutic target for enhancing anti-tumor immunity. Th1 cells could increase the expression of anti-tumor immunity genes to produce more IFNγ and TNFα in the lung (46). The cell balance between immune cell subsets in lung cancer controlled immune homeostasis and tumor growth, so immune cell differentiation pathways were followed with interest. Th17 cells, directly or *via* other cytokines, modulate antitumor immune responses (47). NSCLC samples were processed to detect Th17 cells and Treg cells by flow cytometry, and the concentrations of IL-1β, IL-6, IL-10, IL-17, IL-23, and TGF-β1 were measured by enzyme-linked immunosorbent assay analysis. The Th17/Treg ratio and the related cytokines (IL-6, IL-1β, and IL-23) were significantly higher in NSCLC patients compared with healthy controls. The Th17 cell differentiation pathway was involved in the immunopathology of NSCLC. A distinct cytokine environment in the differentiation of the Th17 may be beneficial in the treatment of NSCLC (48). Cytokines are reliable serum markers especially desirable for malignancies like NSCLC. The interactions of cytokine/cytokine receptor levels and interactions of cytokines, such as IL-6 and IL-8, TNF alpha, soluble TNF (sTNF) RI, IL-2 receptor-alpha, IL-10, granulocyte colony-stimulating factor, soluble vascular endothelial growth factor, and fibroblast growth factor, became significant predictors in patients with NSCLC (49). The systematic study on immune-related pathways between the high- and low-immunity subtypes in LUAD and LUSC provided some potential gene functions and regulatory mechanisms in immune system.

In terms of immune-related gene signature for LUAD and LUSC, the present study found that the seven-immune-related-gene-signature (CD1B, CHRNA6, CLEC12B, CLEC17A, CLNK, INHA, and SLC14A2) prognostic model-based high- and low-risk groups were significantly associated with LUAD overall survival and clinical characteristics. The eight-immune-related-gene-signature (C4BPB, FCAMR, GRAPL, MAP1LC3C, MGC2889, TRIM55, UGT1A1, and VIPR2) prognostic model-based high- and low-risk groups were significantly related to LUSC overall survival and clinical characteristics. Those identified key immune-related genes have been reported in lung cancer or immune system—for example, hUGT1A1 may attenuate immune response. The number of hepatic CD4(+) and CD8(+) cells would be increased with a hepatic venous injection of pcDNA3hUGT1A1 that expressed human bilirubin glucuronosyl transferase 1A1 in rat (50), and UGT1A1 can encode UDP-glucuronosyltransferase that is an enzyme in glucuronidation pathway. A comprehensive analysis of UGT1A polymorphisms could predict tumor response and treatment outcome in NSSLC treated with irinotecan and cisplatin, specifically UGT1A1*6 (51). CLNK, a member of the SLP76 family of adaptors, plays a role to regulate immunoreceptor signaling, including FC-epsilon R1-mediated mast cell degranulation and PLC-gamma-mediated B cell antigen receptor signaling. CLNK was dispensable for the normal differentiation and functions of mast cells, NK cells, and T cells (52). FCAMR is a receptor of Fc fragment of IgA and IgM, with a unique expression profile of FCAMR. FCAMR expressed specially on follicular dendritic cells and marginal zone B cells, which suggests that FCAMR is involved in humoral immune responses. Additionally, it demonstrated that FCAMR downregulated the retention of the IgM immune complex with T-independent antigen on marginal zone B cells and follicular dendritic cells because of endocytosis of the IgM immune complex to suppress germinal center formation, affinity maturation, and memory B cell generation for response to T-independent antigen challenge (53). CD1B was structurally associated with MHC proteins and formed heterodimers with beta-2-microglobulin. The CD1 family can mediate the presentation of primarily lipid and glycolipid antigens of self or microbial origin to T cells (54). Recent advances have thrust CD1 family into the limelight about lipid presentation, T cell populations, and the role of CD1 molecules in the engagement of human γδ T cells (55). VIPR2, a neuroendocrine mediator in immune tissues, affects many T cell functions. The generation of IFN-γ is significantly enhanced by antigen-stimulated T cells in physiological concentrations of VIP, and it was reported that the enhancement of IFN-γ secretion was increased up to a maximum of 14-fold for the VIPR2-selective agonist (56). INHA encoded a member of TGF-beta superfamily of proteins to regulate multiple cellular processes, including cell proliferation, apoptosis, hormone secretion, and immune response (57). The construction of immune-related-gene-signature prognostic models in LUAD and LUSC would promote individualized treatment and provide potential novel targets for immunotherapy.

## Conclusion

Lung cancer is a highly heterogeneous cancer with multiple subtypes such as LUAD and LUSC, which have been used to understand the pathogenesis difference, select tissues for molecular diagnosis, and decide therapeutic strategies. It is absolutely necessary to develop personalized therapy and precise histological characteristics for lung cancer immunotherapy. This study generates the seven-immune-related-gene-signature (CD1B, CHRNA6, CLEC12B, CLEC17A, CLNK, INHA, and SLC14A2) model in LUAD and the eight-immune-related-gene-signature (C4BPB, FCAMR, GRAPL, MAP1LC3C, MGC2889, TRIM55, UGT1A1, and VIPR2) model in LUSC, which can not only predict survival outcome but also reflect the immune status of lung cancers. This gene signature might be clinically used for the improvement of individualized therapy based on the risk score and possible response to immunotherapy.

## Data Availability Statement

Publicly available datasets were analyzed in this study. This data can be found here: (https://portal.gdc.cancer.gov/).

## Ethics Statement

Ethical review and approval was not required for the study on human participants in accordance with the local legislation and institutional requirements. Written informed consent for participation was not required for this study in accordance with the national legislation and the institutional requirements.

## Author Contributions

NL designed the project, analyzed the data, prepared the figures and tables, and wrote the manuscript. JW participated in bioinformatics analysis. XZ conceived the concept, supervised the results, designed, wrote, and critically revised the manuscript, and was responsible for its financial supports and the corresponding works. All authors contributed to the article and approved the submitted version.

## Funding

This work was supported by the Shandong First Medical University Talent Introduction Funds (to XZ), the Hunan Provincial Hundred Talent Plan (to XZ), the Shandong Provincial Natural Science Foundation (ZR202103020356 to XZ), the National Natural Science Foundation of China (82172866), and the Academic Promotion Program of Shandong First Medical University (2019ZL002).

## Conflict of Interest

The authors declare that the research was conducted in the absence of any commercial or financial relationships that could be construed as a potential conflict of interest.

## Publisher’s Note

All claims expressed in this article are solely those of the authors and do not necessarily represent those of their affiliated organizations, or those of the publisher, the editors and the reviewers. Any product that may be evaluated in this article, or claim that may be made by its manufacturer, is not guaranteed or endorsed by the publisher.
