# Supplementary material for: Identification of Immune-Related Gene Signatures in Lung Adenocarcinoma and Lung Squamous Cell Carcinoma
Source: Front Immunol. 2021 Nov 23;12:752643. doi: 10.3389/fimmu.2021.752643 (PMC8649721; doi:10.3389/fimmu.2021.752643)
Supplement: Supplementary file 3 [file DataSheet_3.zip › Supplementary figure 2_v2.pdf]

Supplementary figure 2

The correlation of immune cells with clinicopathological characteristics in LUAD.

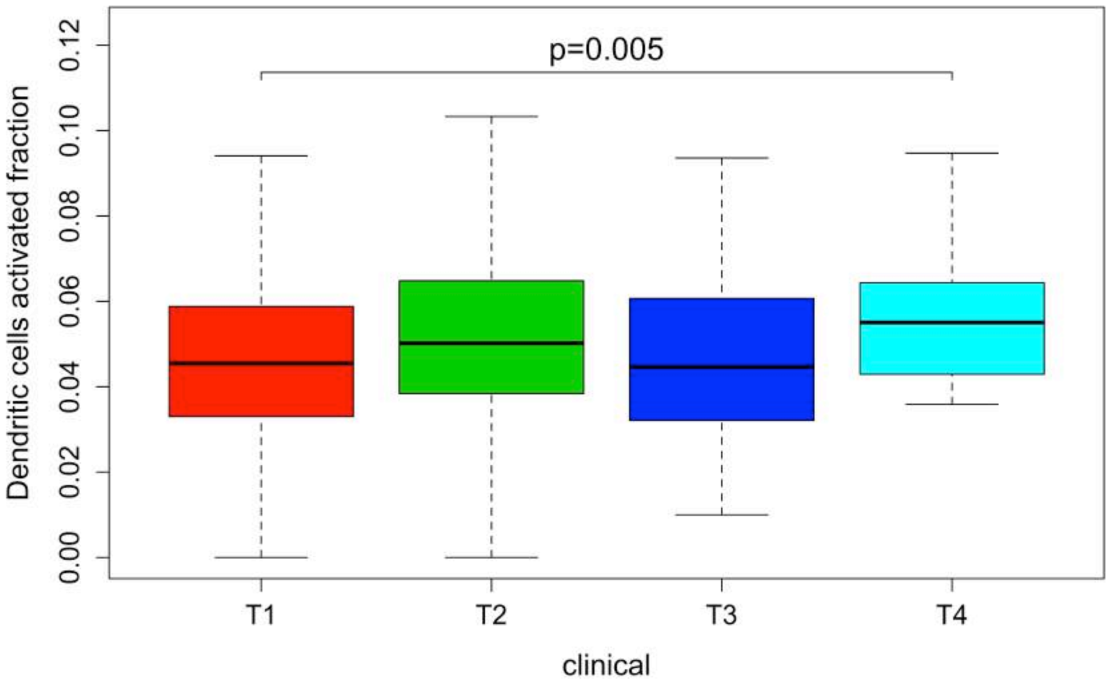

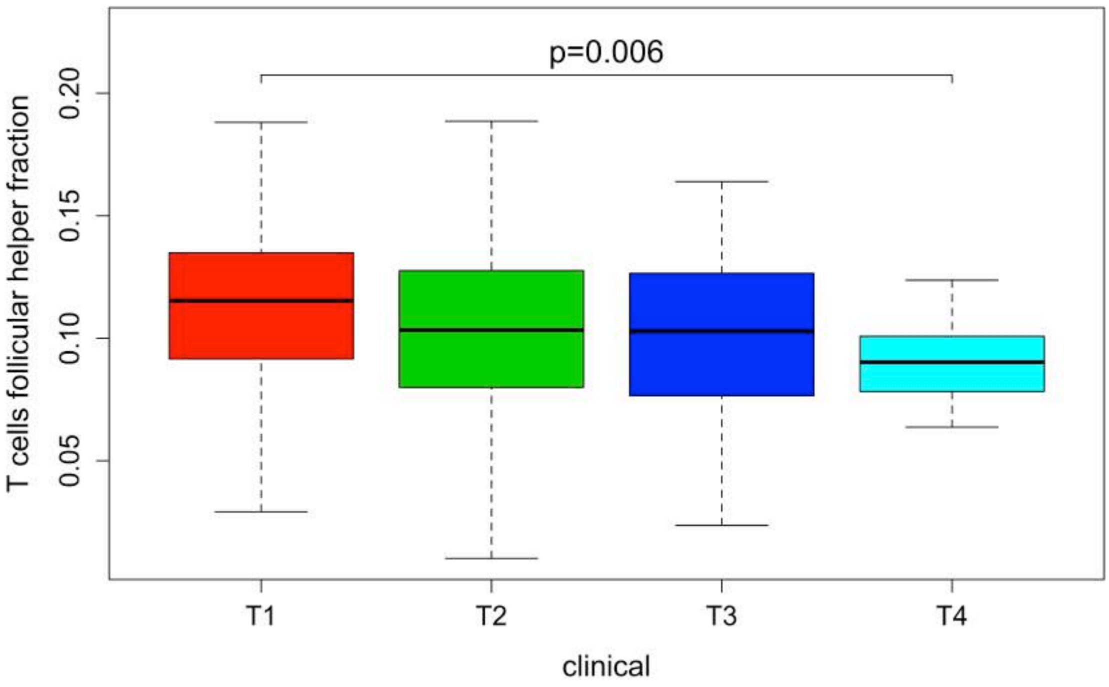
p=0.006
T cells follicular helper fraction
0.05
0.10
0.15
0.20
T1
T2
T3
T4
clinical

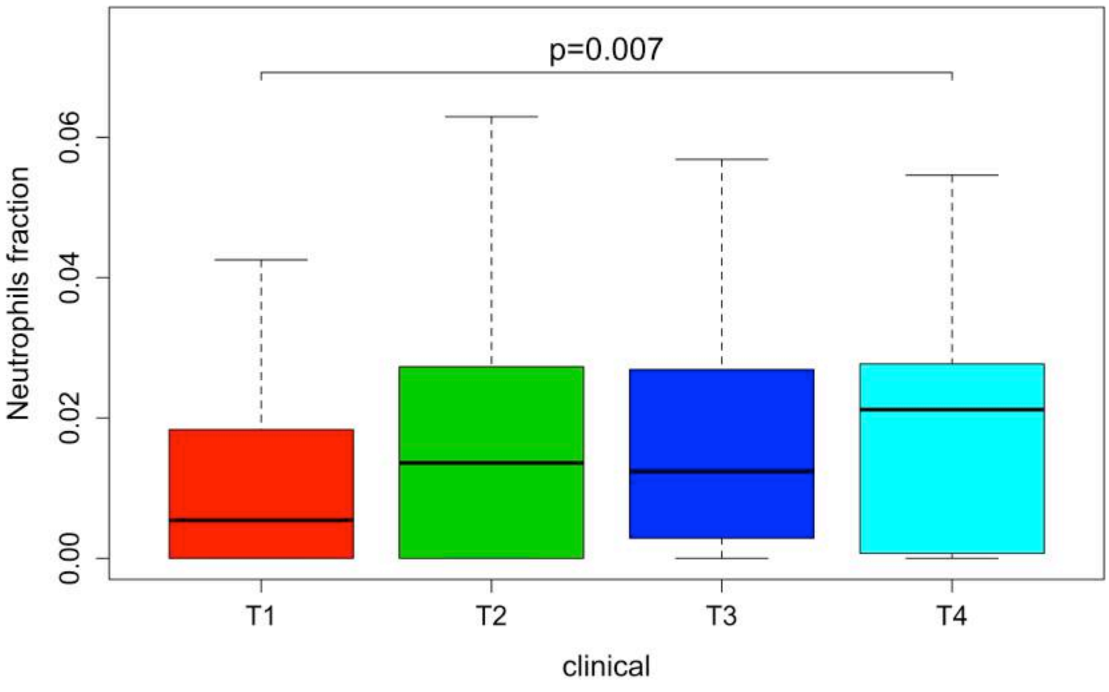
p=0.007
Neutrophils fraction
0.00
0.02
0.04
0.06
T1
T2
T3
T4
clinical

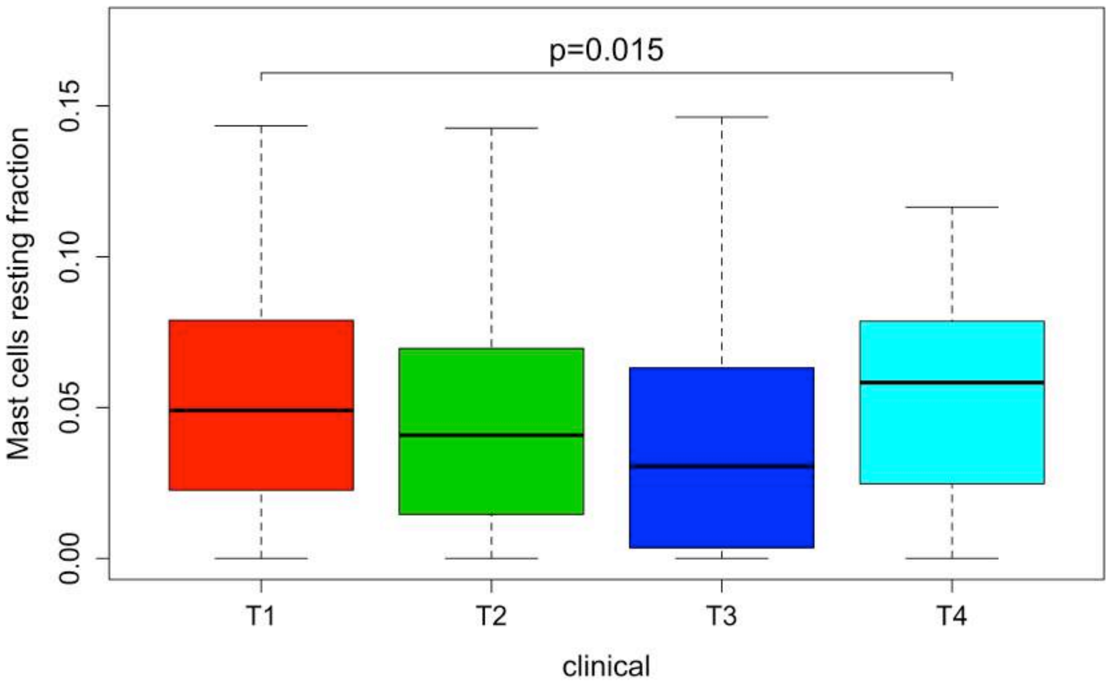

p=0.015
Mast cells resting fraction
0.00
0.05
0.10
0.15
T1
T2
T3
T4
clinical

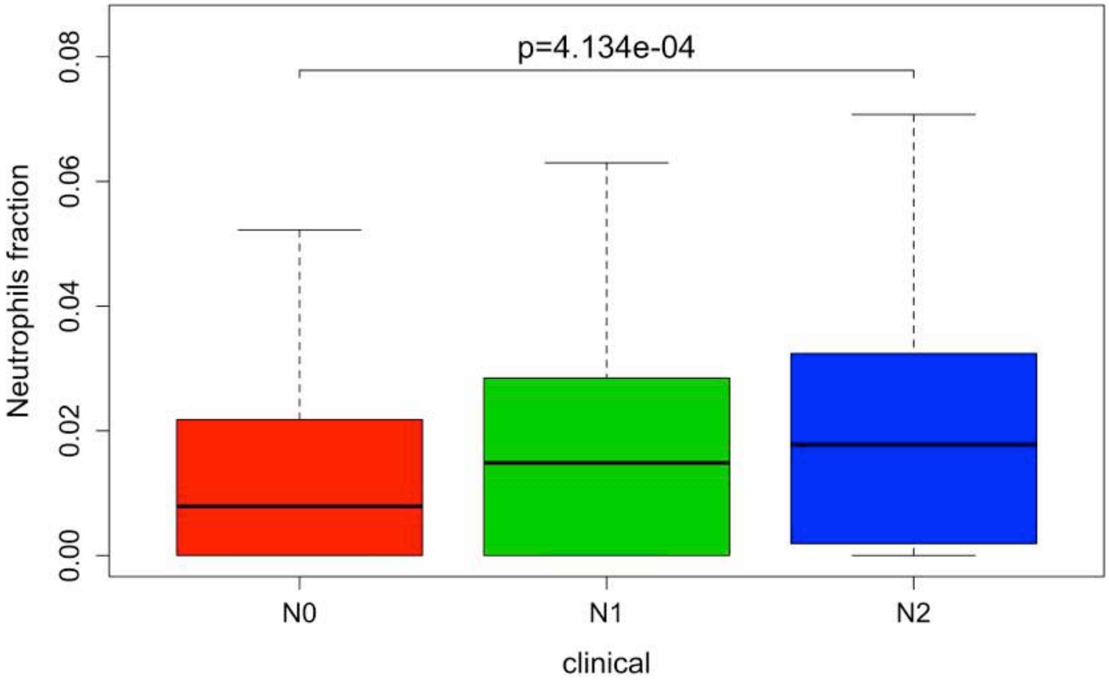

p=4.134e-04
Neutrophils fraction
0.00
0.02
0.04
0.06
0.08
N0
N1
N2
clinical

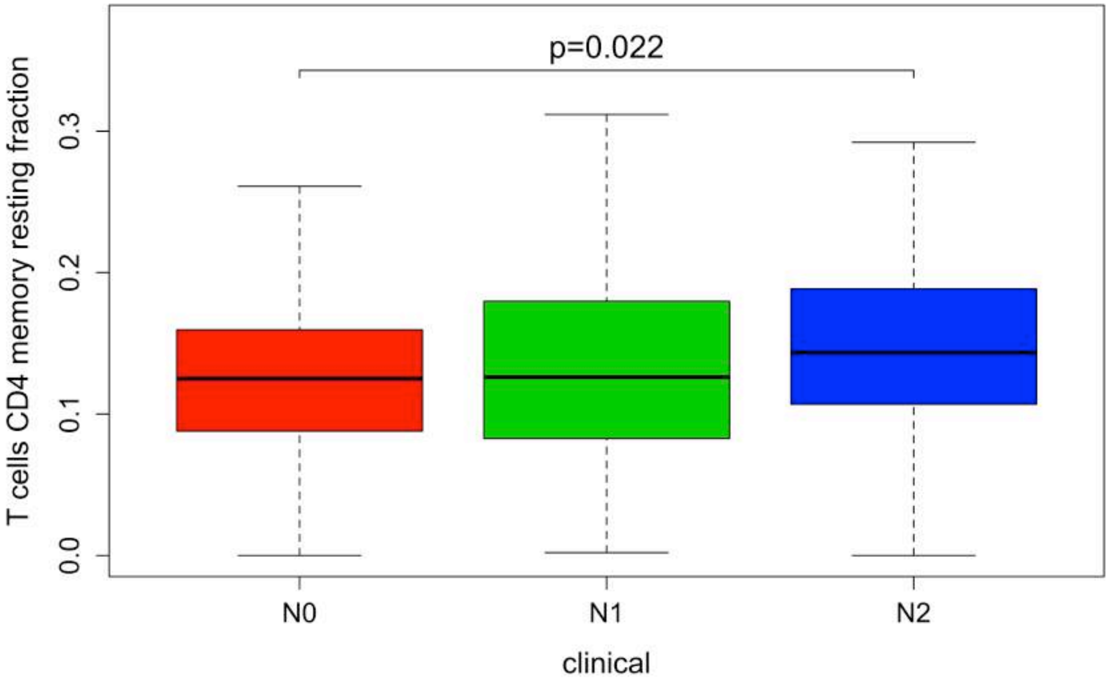

p=0.022
T cells CD4 memory resting fraction
0.0
0.1
0.2
0.3
N0
N1
N2
clinical

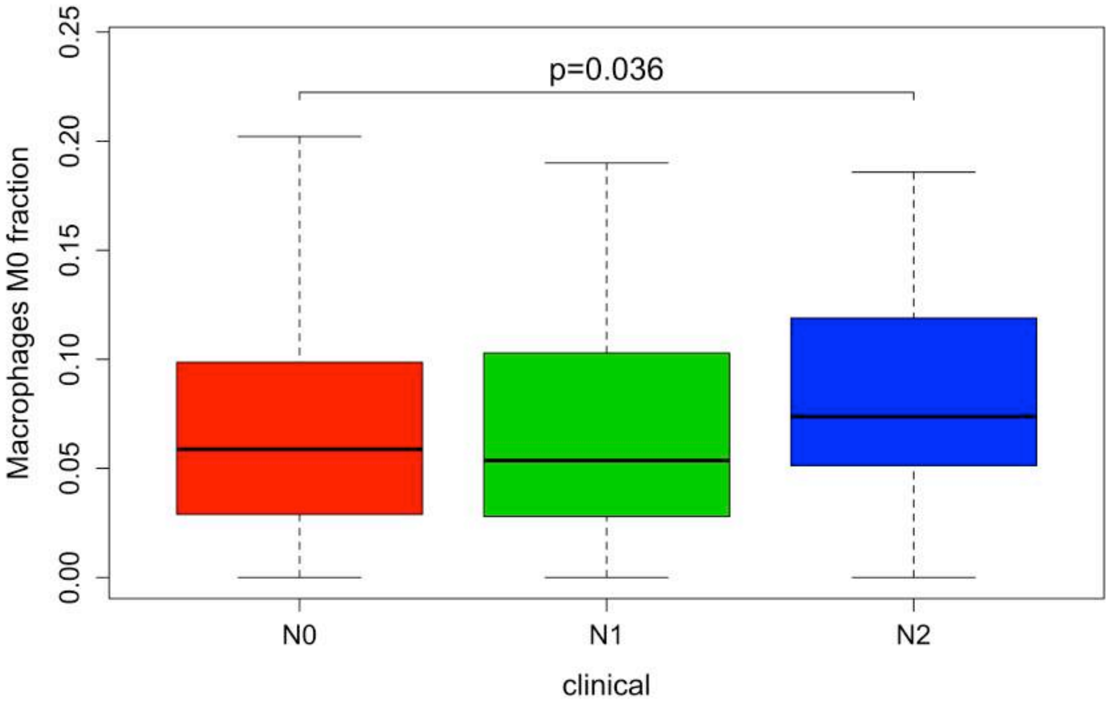

p=0.036
Macrophages M0 fraction
0.00
0.05
0.10
0.15
0.20
0.25
N0
N1
N2
clinical

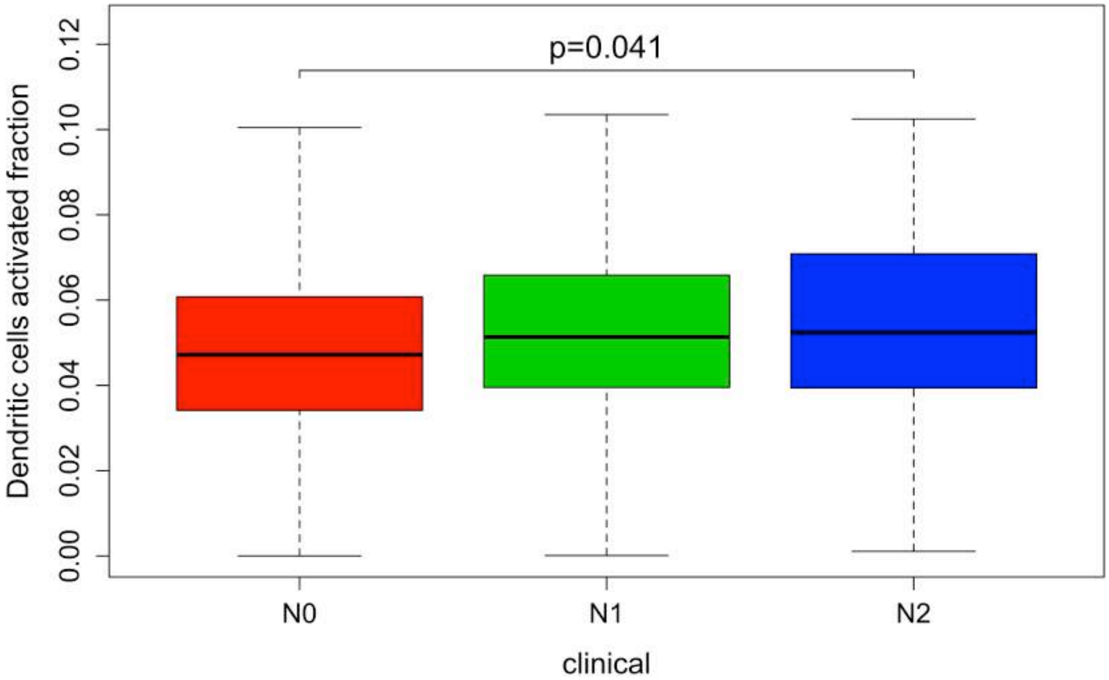

p=0.041
Dendritic cells activated fraction
0.00
0.02
0.04
0.06
0.08
0.10
0.12
N0
N1
N2
clinical

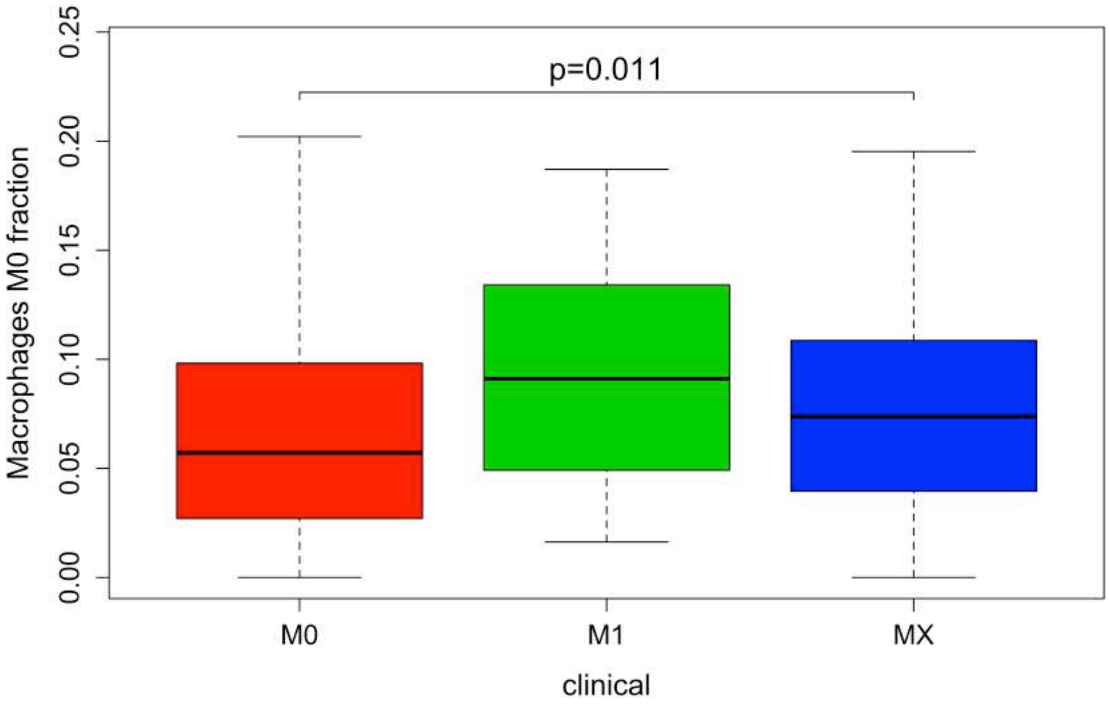
p=0.011
Macrophages M0 fraction
0.00
0.05
0.10
0.15
0.20
0.25
M0
M1
MX
clinical

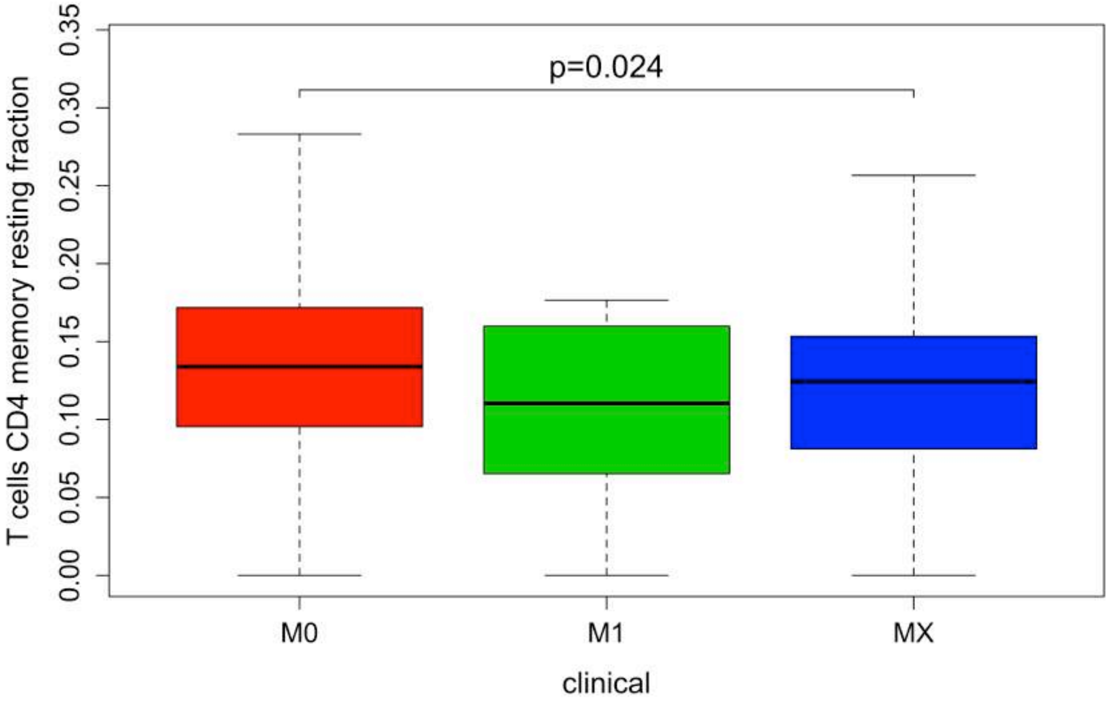
p=0.024
T cells CD4 memory resting fraction
0.00
0.05
0.10
0.15
0.20
0.25
0.30
0.35
M0
M1
MX
clinical

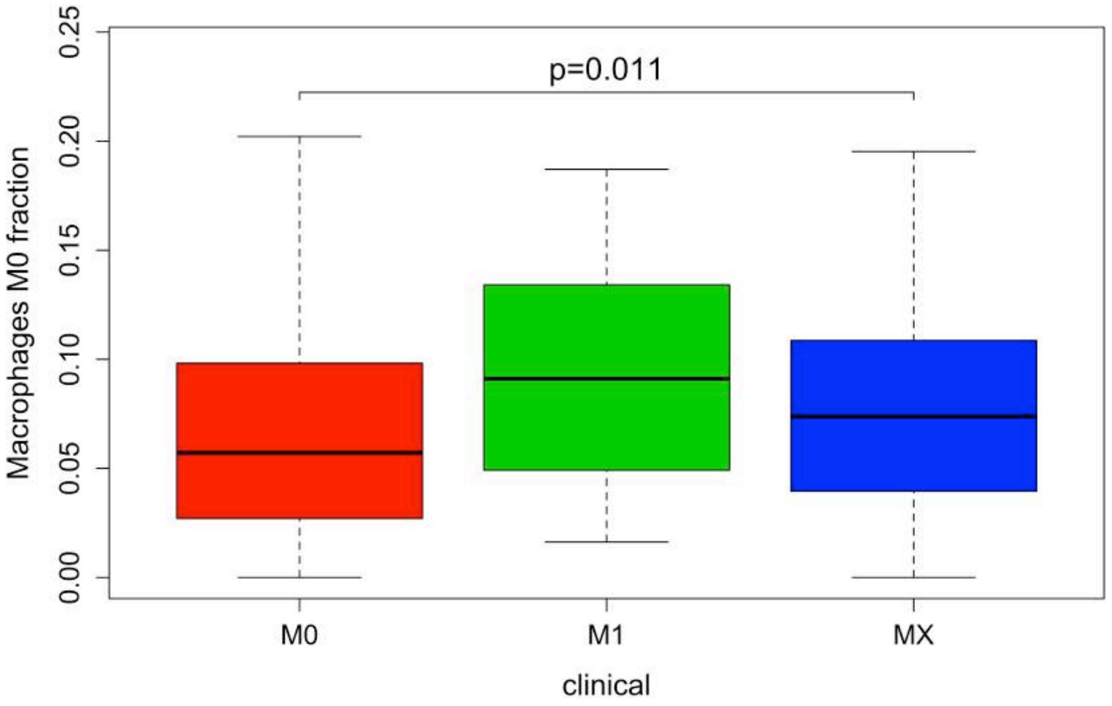

p=0.011
Macrophages M0 fraction
0.00
0.05
0.10
0.15
0.20
0.25
M0
M1
MX
clinical

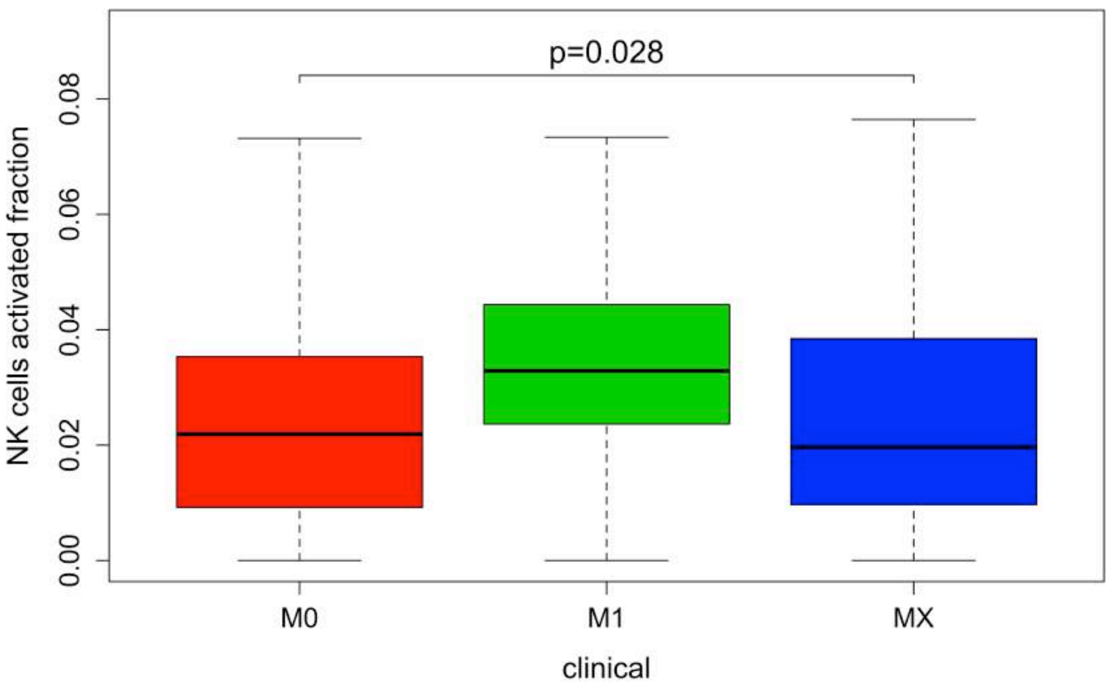

p=0.028
NK cells activated fraction
0.00
0.02
0.04
0.06
0.08
M0
M1
MX
clinical

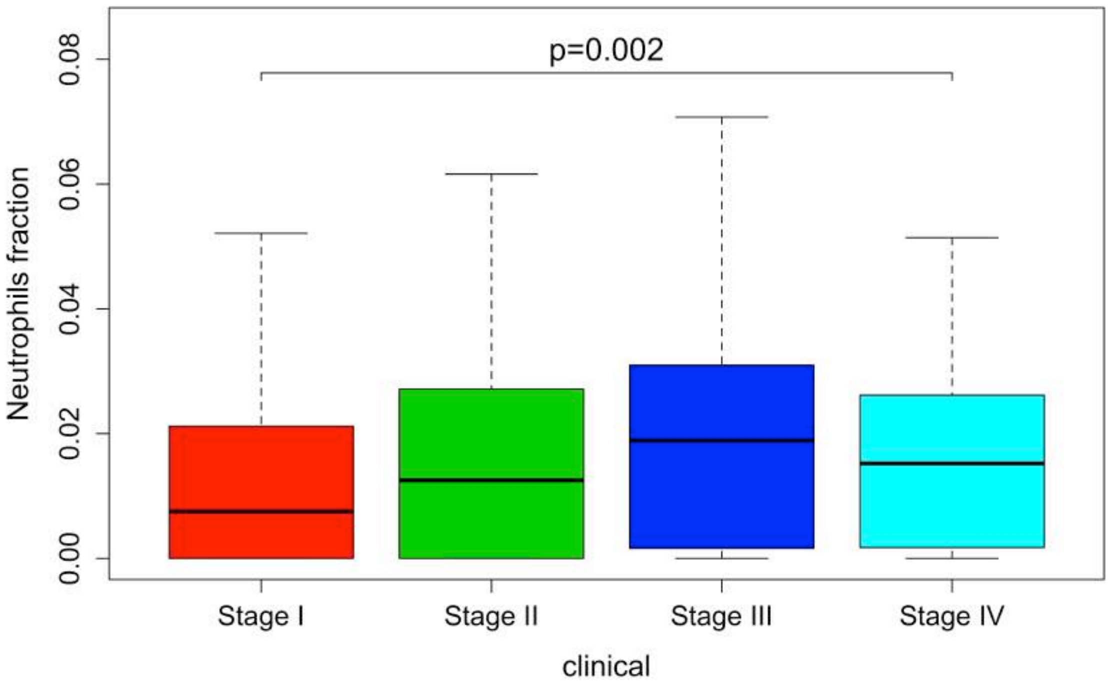
p=0.002
Neutrophils fraction
0.00
0.02
0.04
0.06
0.08
Stage I
Stage II
Stage III
Stage IV
clinical

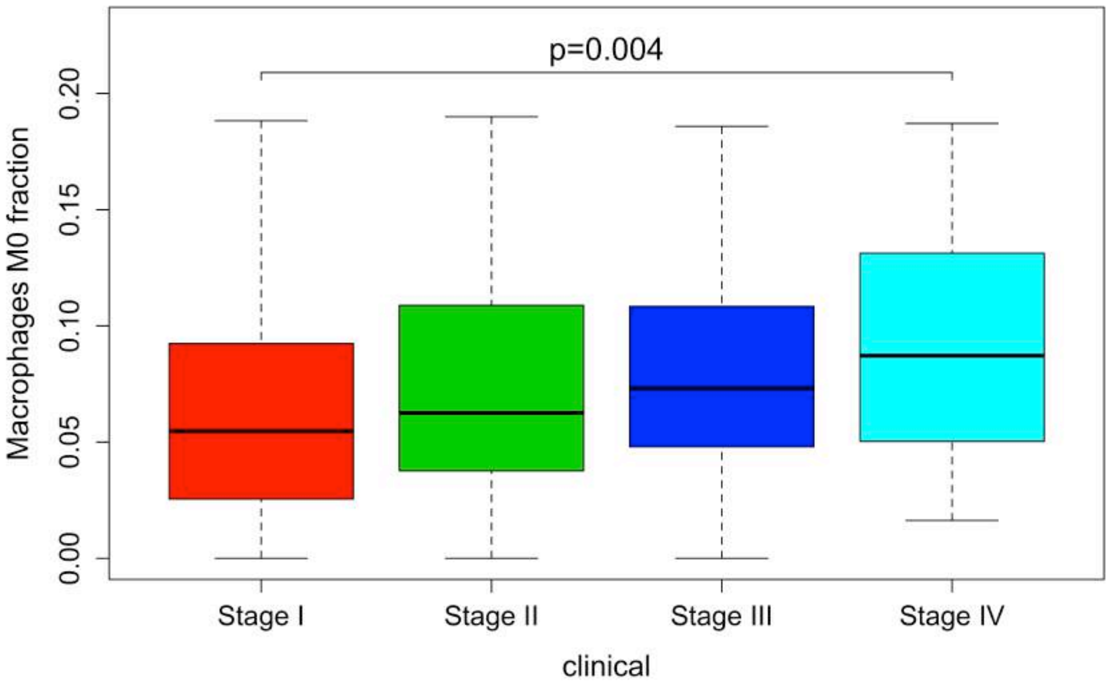
p=0.004
Macrophages M0 fraction
0.00
0.05
0.10
0.15
0.20
Stage I
Stage II
Stage III
Stage IV
clinical

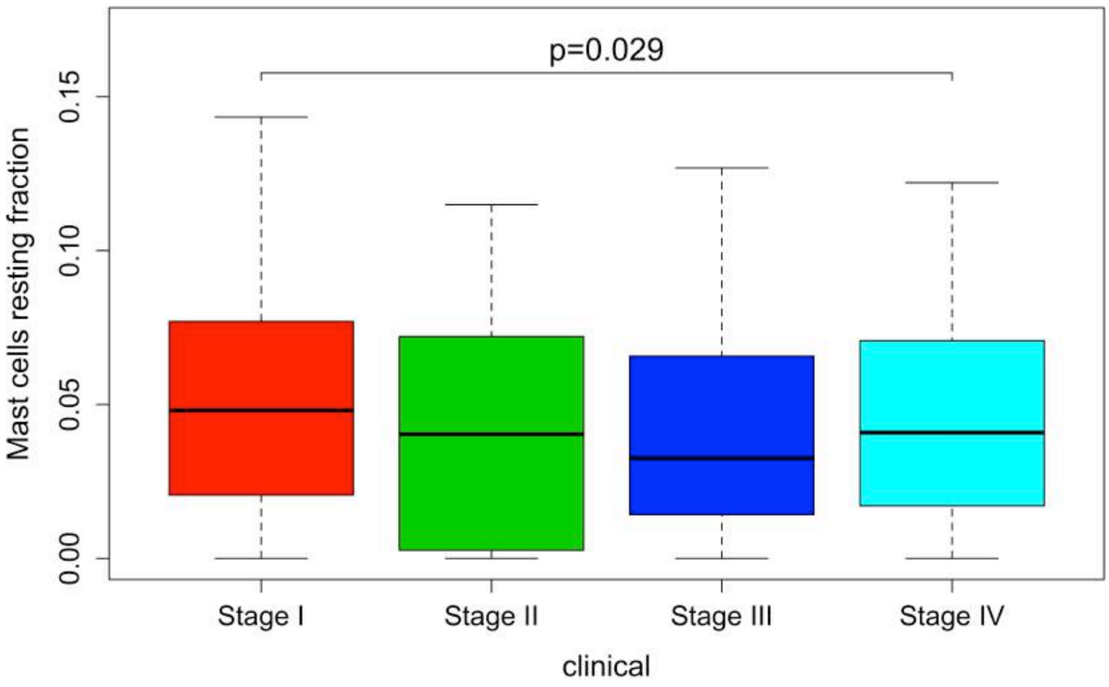

p=0.029
Mast cells resting fraction
0.00
0.05
0.10
0.15
Stage I
Stage II
Stage III
Stage IV
clinical

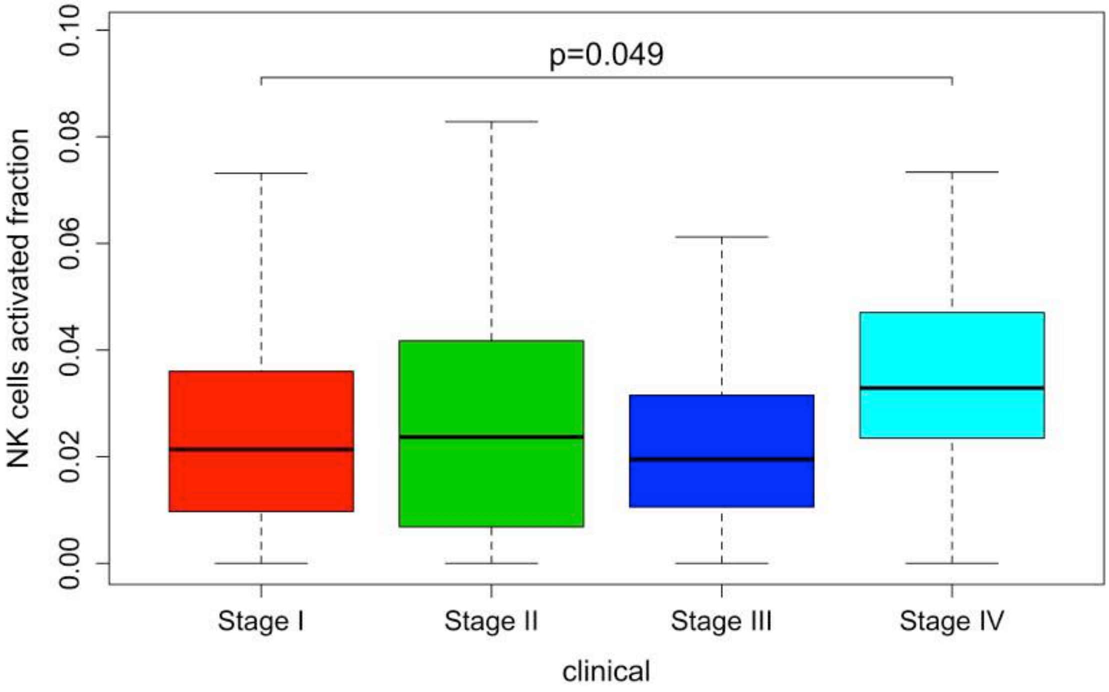

p=0.049
NK cells activated fraction
0.00
0.02
0.04
0.06
0.08
0.10
Stage I
Stage II
Stage III
Stage IV
clinical

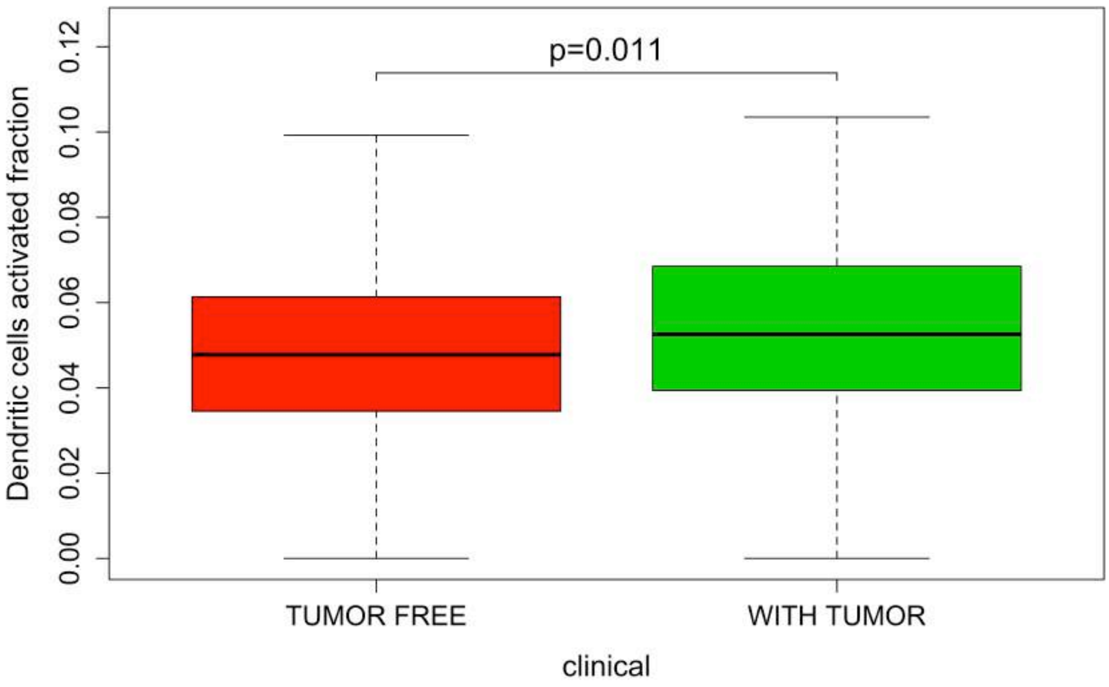
p=0.011
Dendritic cells activated fraction
0.00
0.02
0.04
0.06
0.08
0.10
0.12
TUMOR FREE
WITH TUMOR
clinical

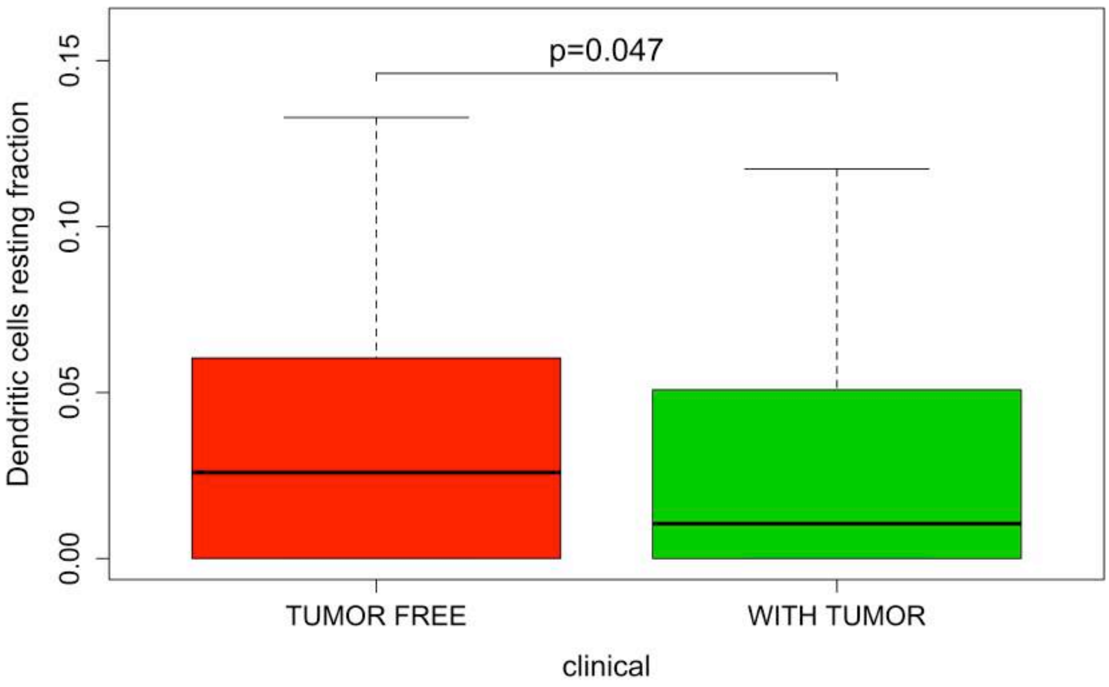
p=0.047
Dendritic cells resting fraction
0.00
0.05
0.10
0.15
TUMOR FREE
WITH TUMOR
clinical
