# Supplementary material for: Identification of Immune-Related Gene Signatures in Lung Adenocarcinoma and Lung Squamous Cell Carcinoma
Source: Front Immunol. 2021 Nov 23;12:752643. doi: 10.3389/fimmu.2021.752643 (PMC8649721; doi:10.3389/fimmu.2021.752643)
Supplement: Supplementary file 3 [file DataSheet_3.zip › Supplementary figure 3_v2.pdf]

Supplementary figure 3

The correlation of immune cells with clinicopathological characteristics in LUSC.

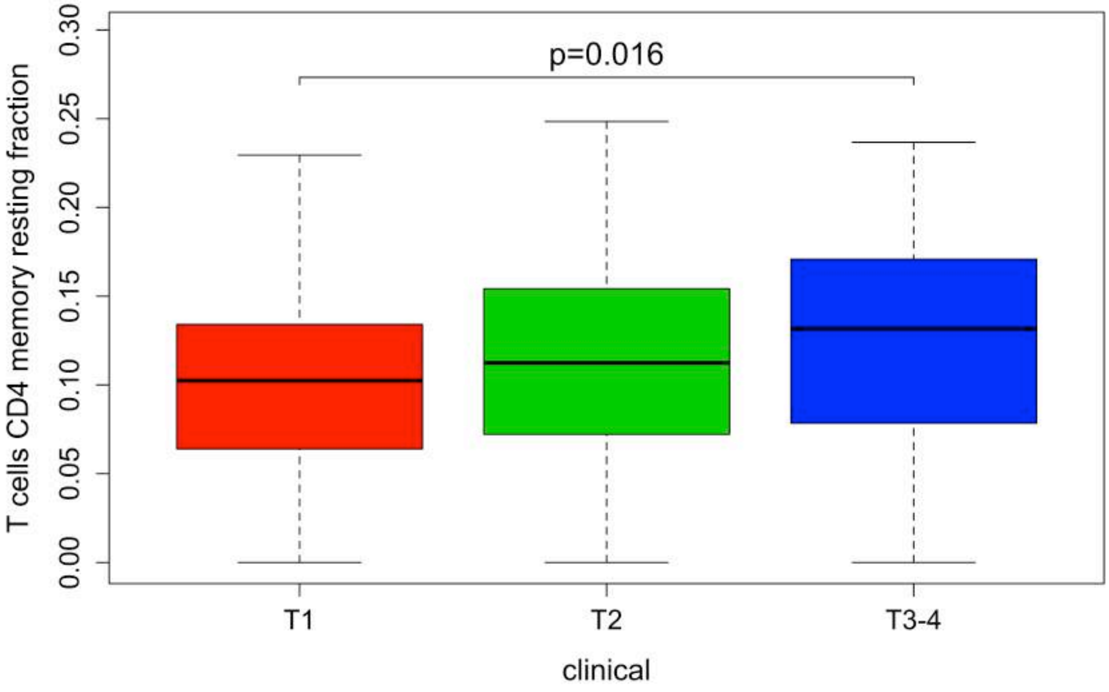

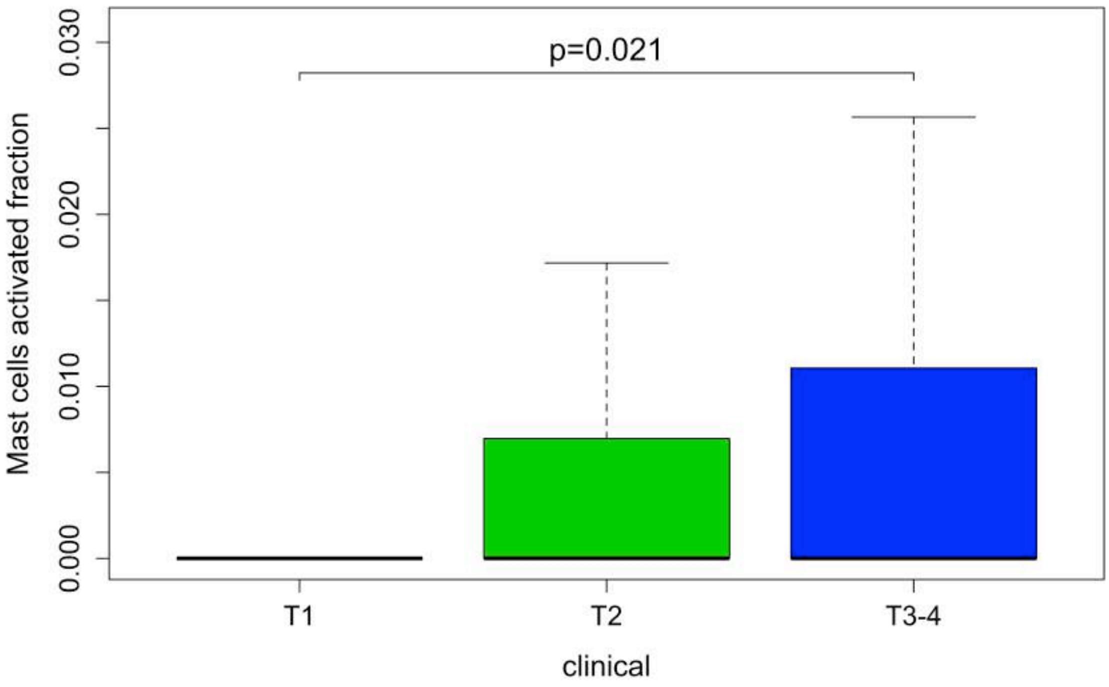
p=0.021
Mast cells activated fraction
0.000
0.010
0.020
0.030
T1
T2
T3-4
clinical

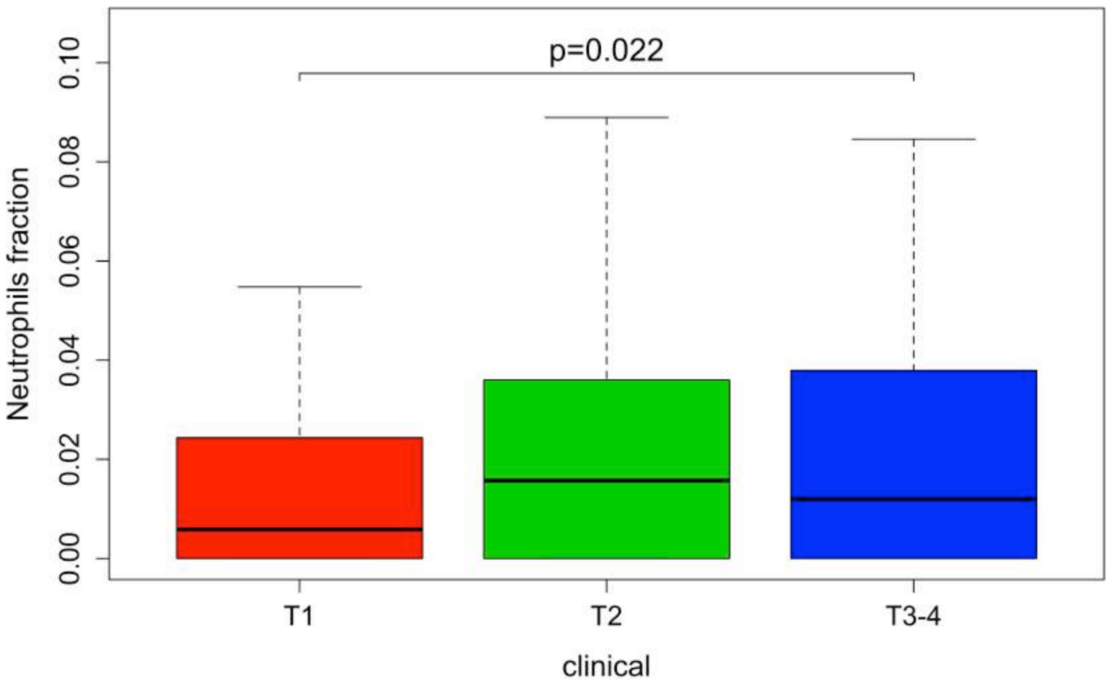
p=0.022
Neutrophils fraction
0.00
0.02
0.04
0.06
0.08
0.10
T1
T2
T3-4
clinical

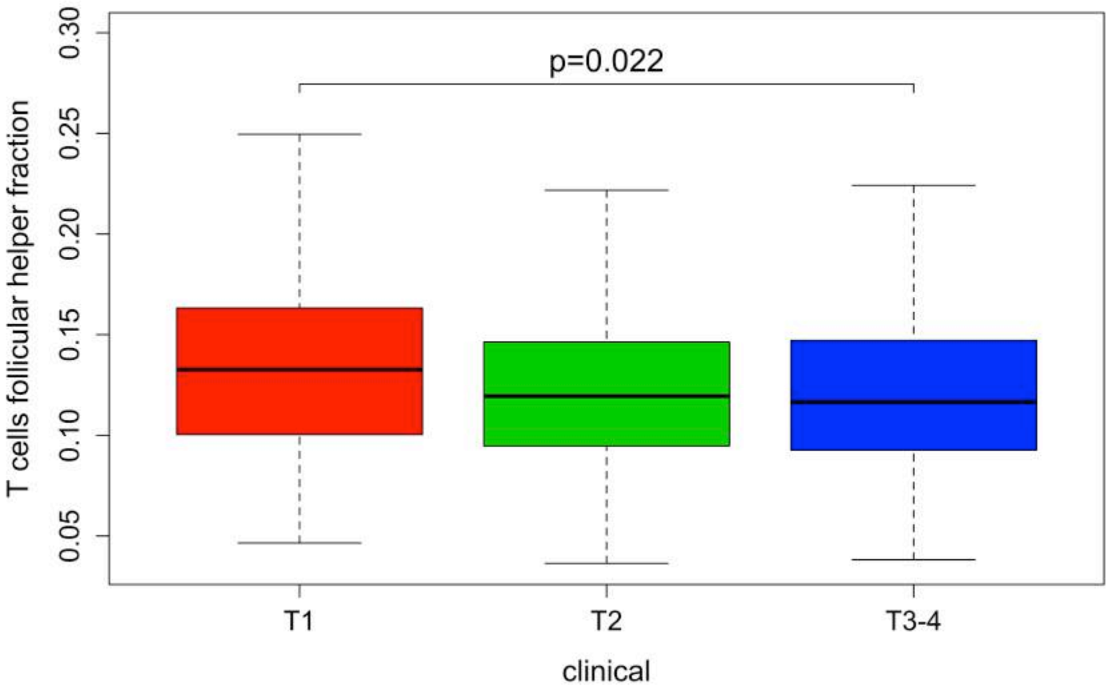
p=0.022
T cells follicular helper fraction
0.05
0.10
0.15
0.20
0.25
0.30
T1
T2
T3-4
clinical

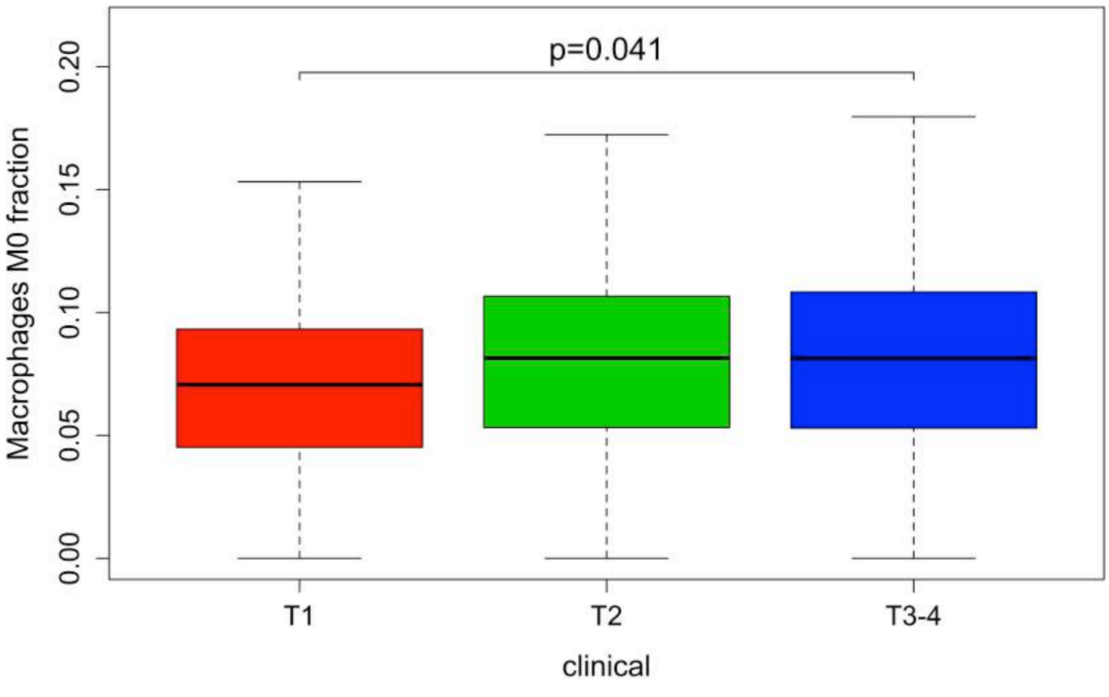
p=0.041
Macrophages M0 fraction
0.00
0.05
0.10
0.15
0.20
T1
T2
T3-4
clinical

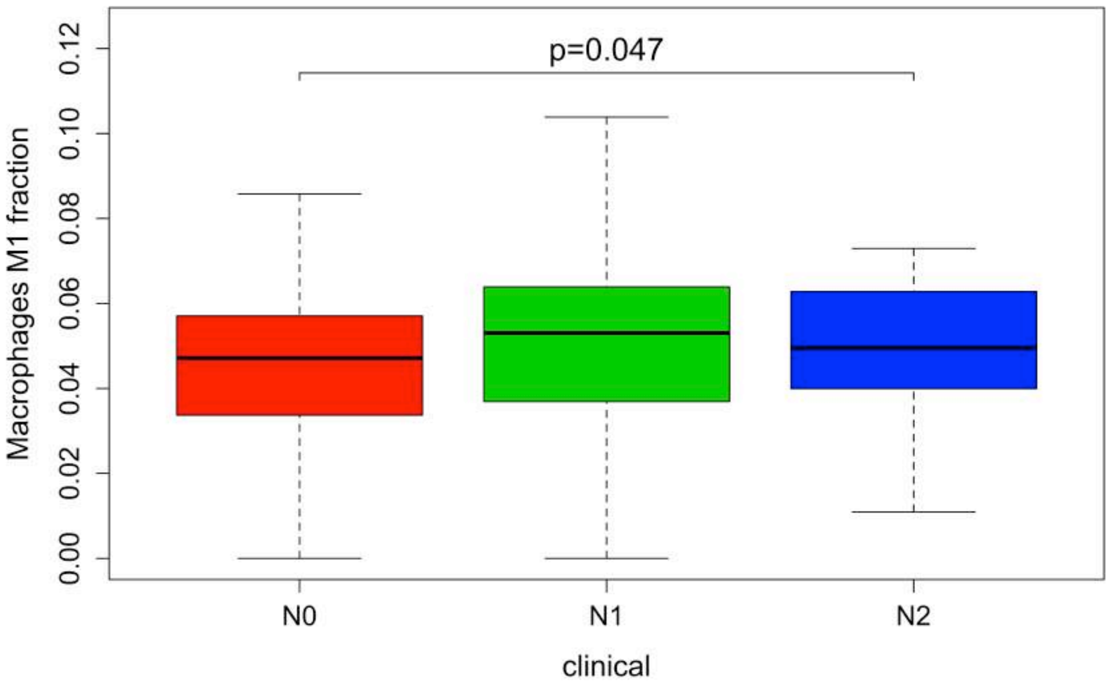
p=0.047
Macrophages M1 fraction
0.00
0.02
0.04
0.06
0.08
0.10
0.12
N0
N1
N2
clinical

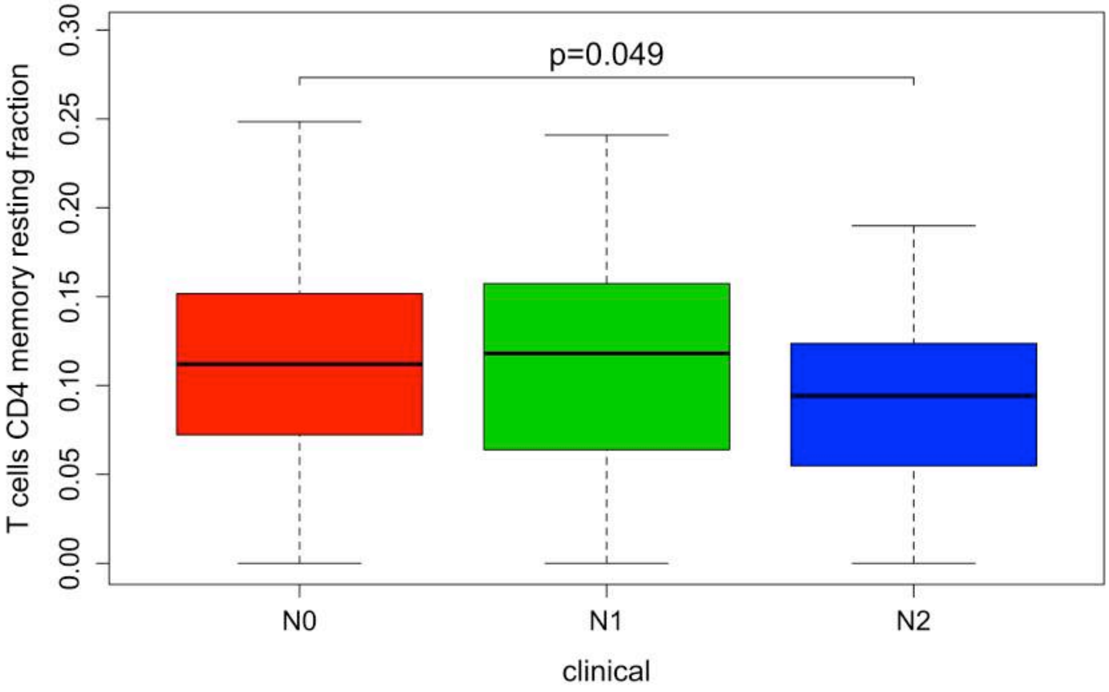
p=0.049
T cells CD4 memory resting fraction
0.00
0.05
0.10
0.15
0.20
0.25
0.30
N0
N1
N2
clinical

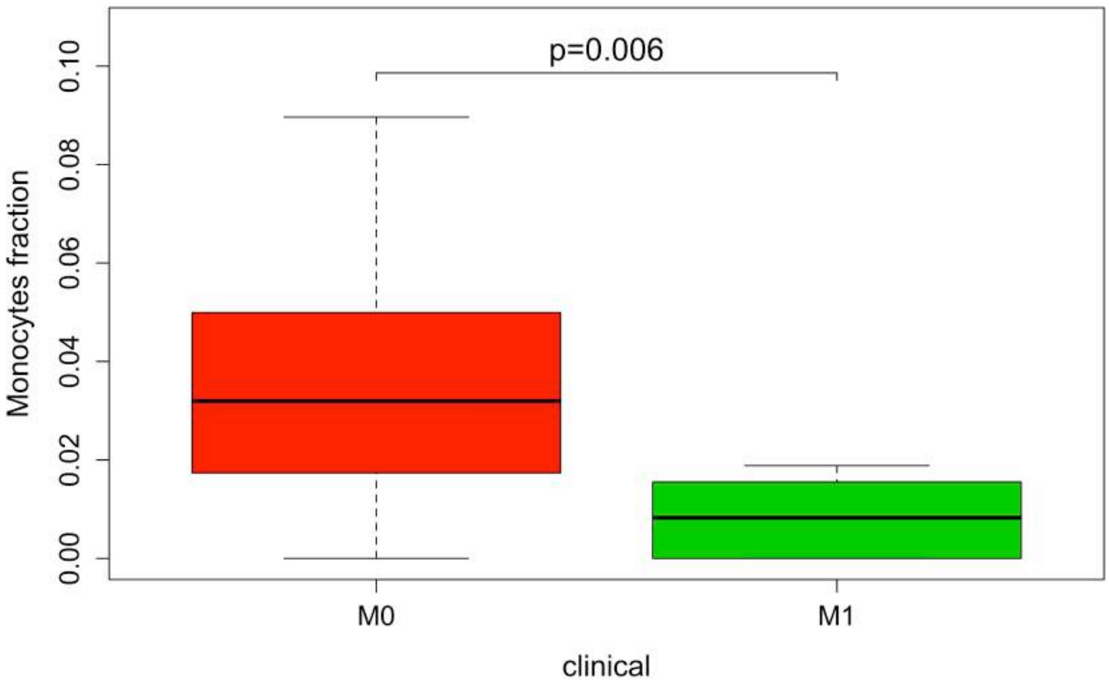
p=0.006
Monocytes fraction
0.00
0.02
0.04
0.06
0.08
0.10
M0
M1
clinical

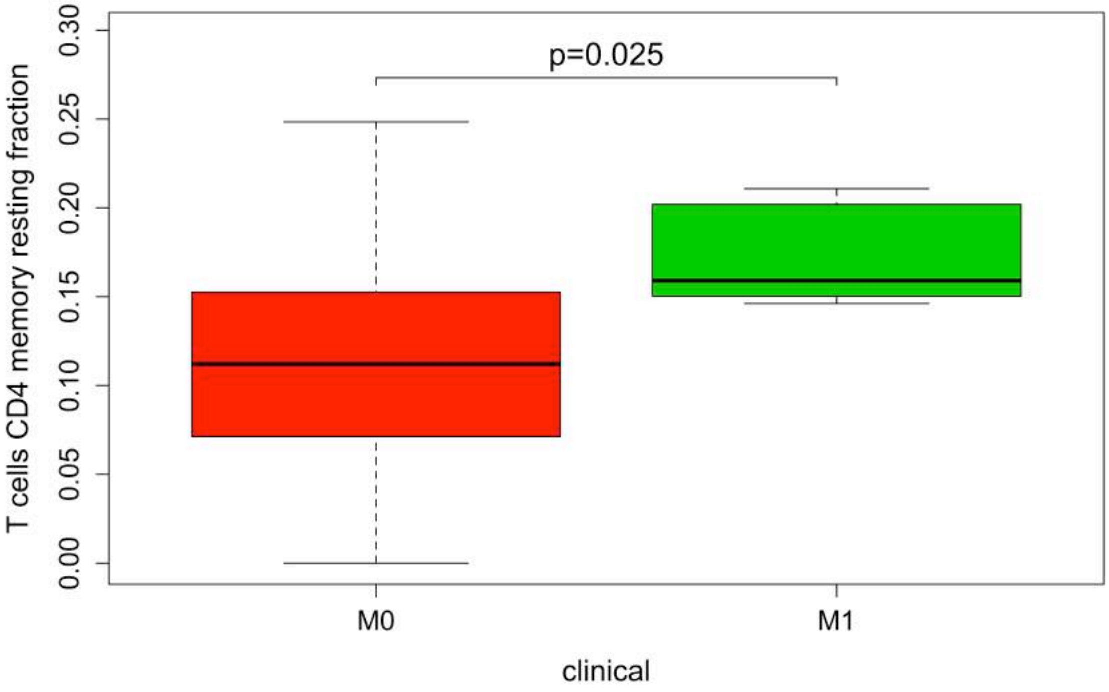
p=0.025
T cells CD4 memory resting fraction
0.00
0.05
0.10
0.15
0.20
0.25
0.30
M0
M1
clinical

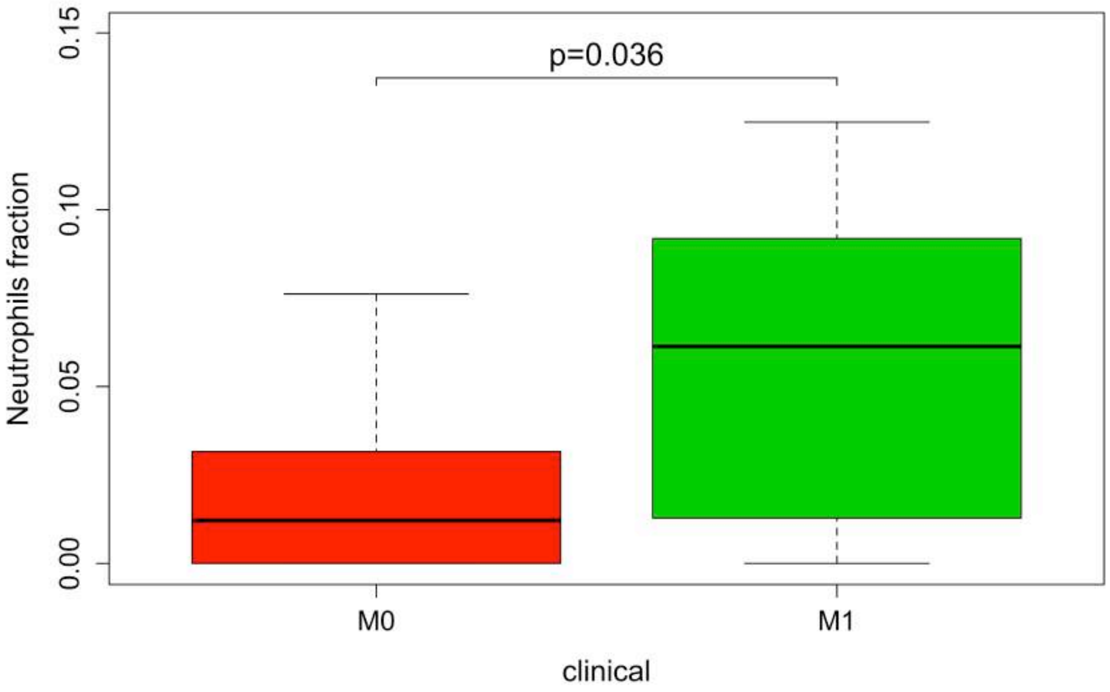

p=0.036
Neutrophils fraction
0.00
0.05
0.10
0.15
M0
M1
clinical

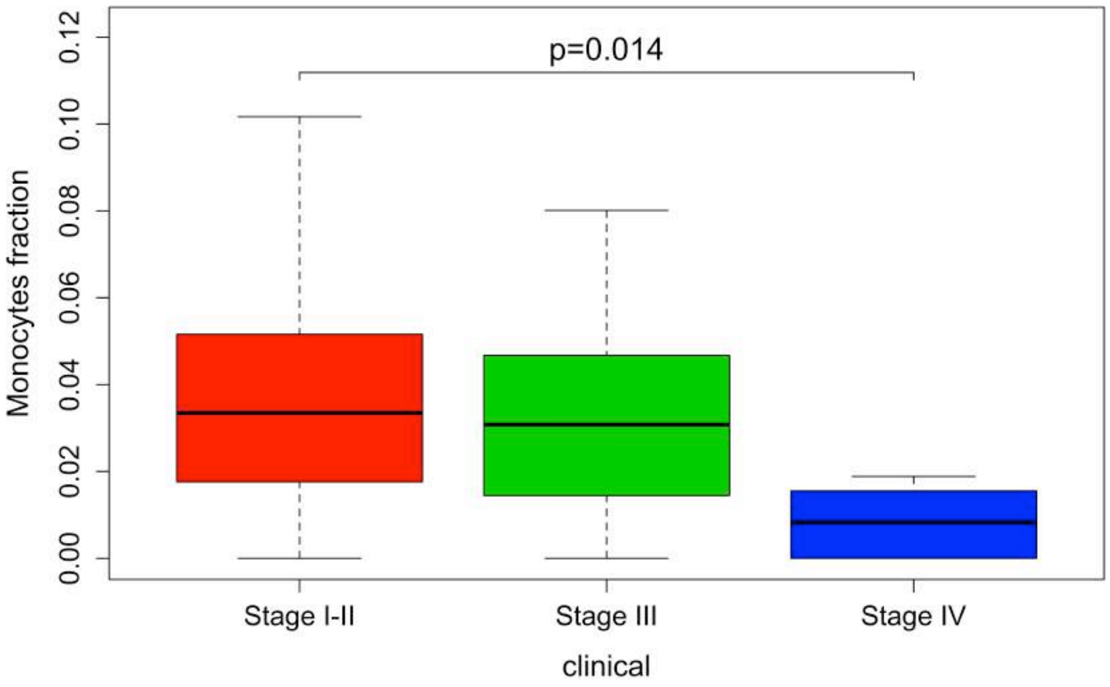

p=0.014
Monocytes fraction
0.00
0.02
0.04
0.06
0.08
0.10
0.12
Stage I-II
Stage III
Stage IV
clinical
