# Supplementary material for: Identification of Immune-Related Gene Signatures in Lung Adenocarcinoma and Lung Squamous Cell Carcinoma
Source: Front Immunol. 2021 Nov 23;12:752643. doi: 10.3389/fimmu.2021.752643 (PMC8649721; doi:10.3389/fimmu.2021.752643)
Supplement: Supplementary file 3 [file DataSheet_3.zip › Supplementary figure 4_v1.pdf]

Supplementary figure 4

Associations between mutation status and immune status in LUAD and LUSC. A. The waterfall plot showed the top 30 mutated genes in LUAD and their mutation information. **B.** The significant co-occurrence of gene mutations in LUAD. **C.** The waterfall plot showed the top 30 mutated genes in LUSC and their mutation information. **D.** The significant co-occurrence of gene mutations in LUSC. **E.** IC50 effect of drugs based on TP53 mutation. **F.** The distribution of TP53 mutations and protein domains in LUAD. **G.** The distribution of TP53 mutations and protein domains in LUSC. **H.** Boxplot showed the ratio difference of 12 immune cells between high and low TMB score subtypes in LUAD, and Wilcoxon rank sum was used for the significance test. **I.** Boxplot showed the ratio difference of 6 immune cells between high and low TMB score subtypes in LUSC, and Wilcoxon rank sum was used for the significance test. TMB: Tumor mutation burden. High and low TMB score subtypes were determined by the comparison with the median of TMB scores in LUAD and LUSC. P value was verified by log-rank test. *$p < 0.05$, **$p < 0.01$, and ***$p < 0.001$.

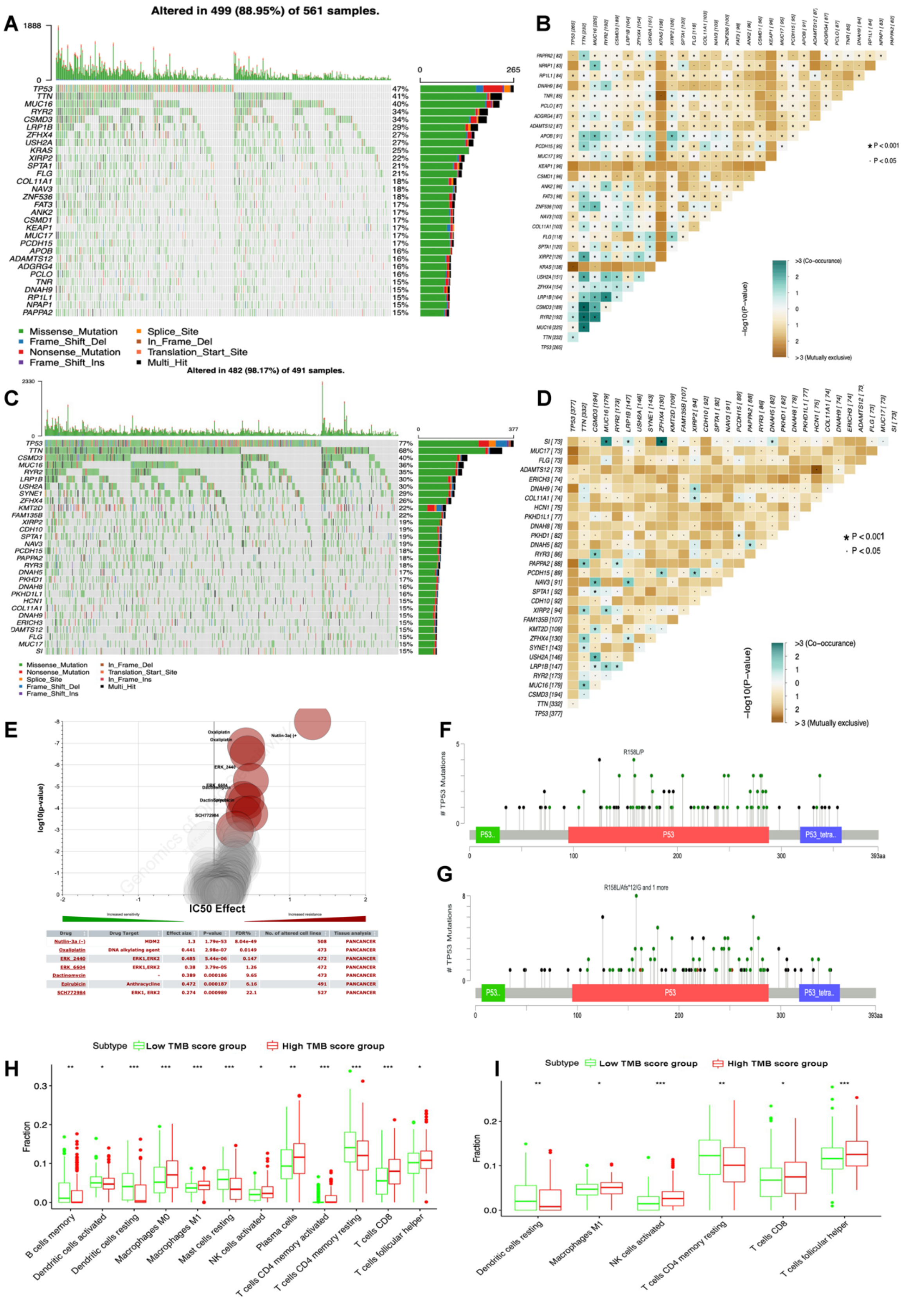

Drug	Drug Target	Effect size	P-value	FDR%	No. of altered cell lines	Tissue analysis
Nutlin-3a (-)	MDM2	1.3	1.79e-53	8.04e-49	508	PANCANCER
Oxaliplatin	DNA alkylating agent	0.441	2.98e-07	0.0149	473	PANCANCER
ERK_2440	ERK1,ERK2	0.485	5.44e-06	0.147	472	PANCANCER
ERK_6604	ERK1,ERK2	0.38	3.79e-05	1.26	472	PANCANCER
Dactinomycin	-	0.389	0.000186	9.65	473	PANCANCER
Epirubicin	Anthracycline	0.472	0.000187	6.16	491	PANCANCER
SCH772984	ERK1, ERK2	0.274	0.000989	22.1	527	PANCANCER
