# Supplementary material for: Identification of Immune-Related Gene Signatures in Lung Adenocarcinoma and Lung Squamous Cell Carcinoma
Source: Front Immunol. 2021 Nov 23;12:752643. doi: 10.3389/fimmu.2021.752643 (PMC8649721; doi:10.3389/fimmu.2021.752643)
Supplement: Supplementary file 3 [file DataSheet_3.zip › Supplementary figure 5_v1.pdf]

Supplementary figure 5

Kaplan–Meier survival analysis for differentially expressed genes in LUAD.

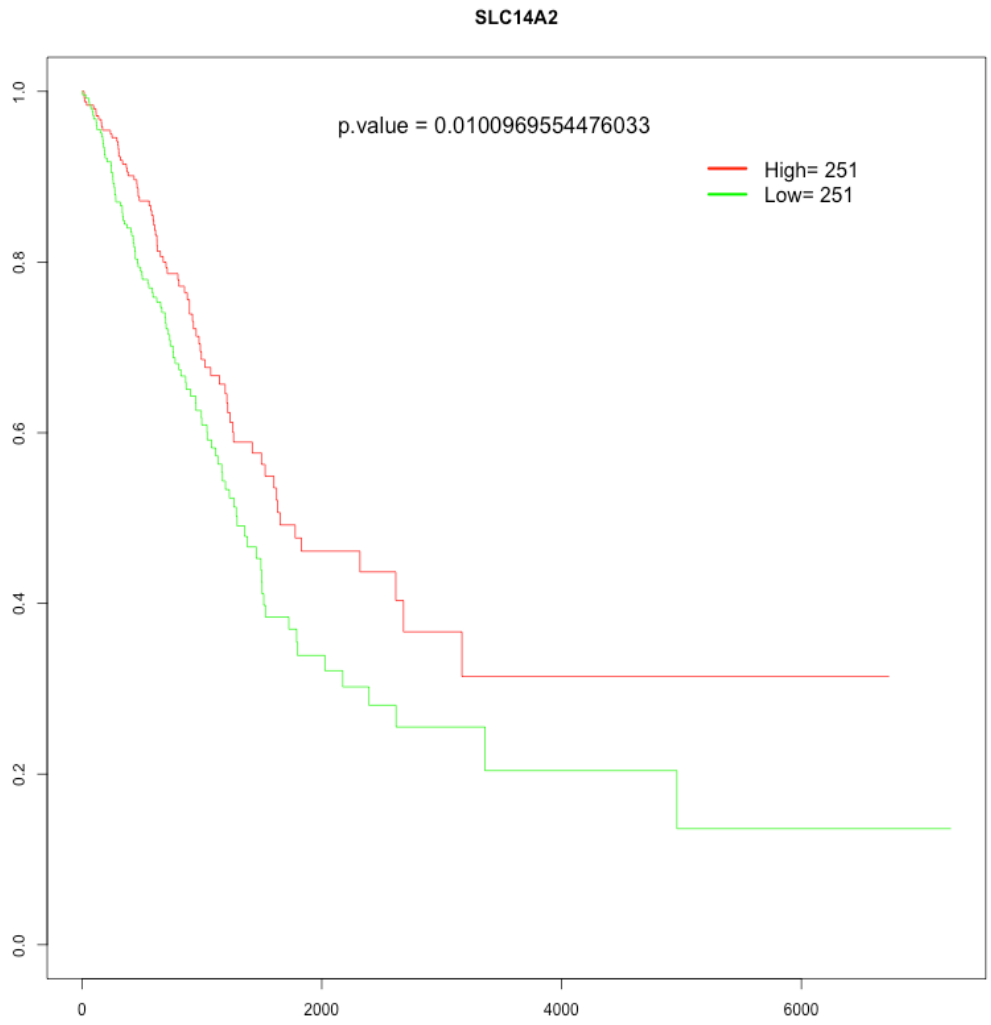

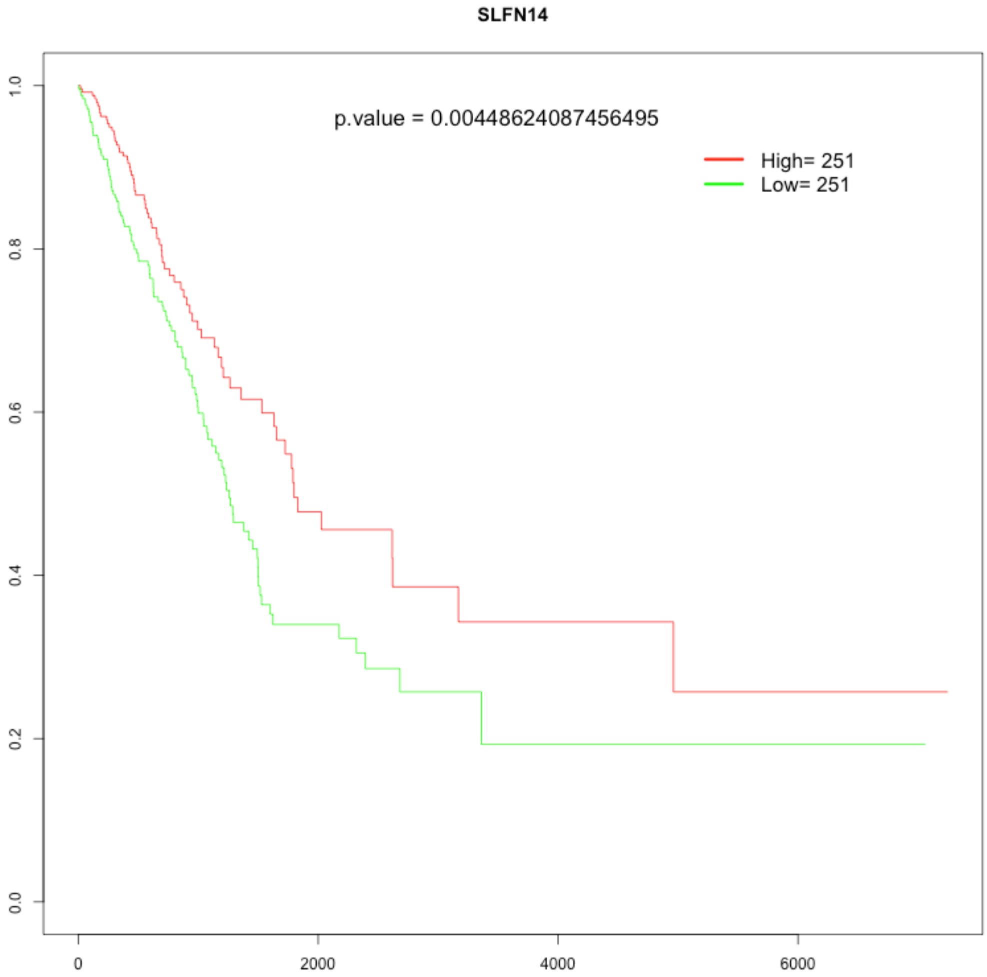

SLFN14
p.value = 0.00448624087456495
High= 251
Low= 251
0.0
0.2
0.4
0.6
0.8
1.0
0
2000
4000
6000

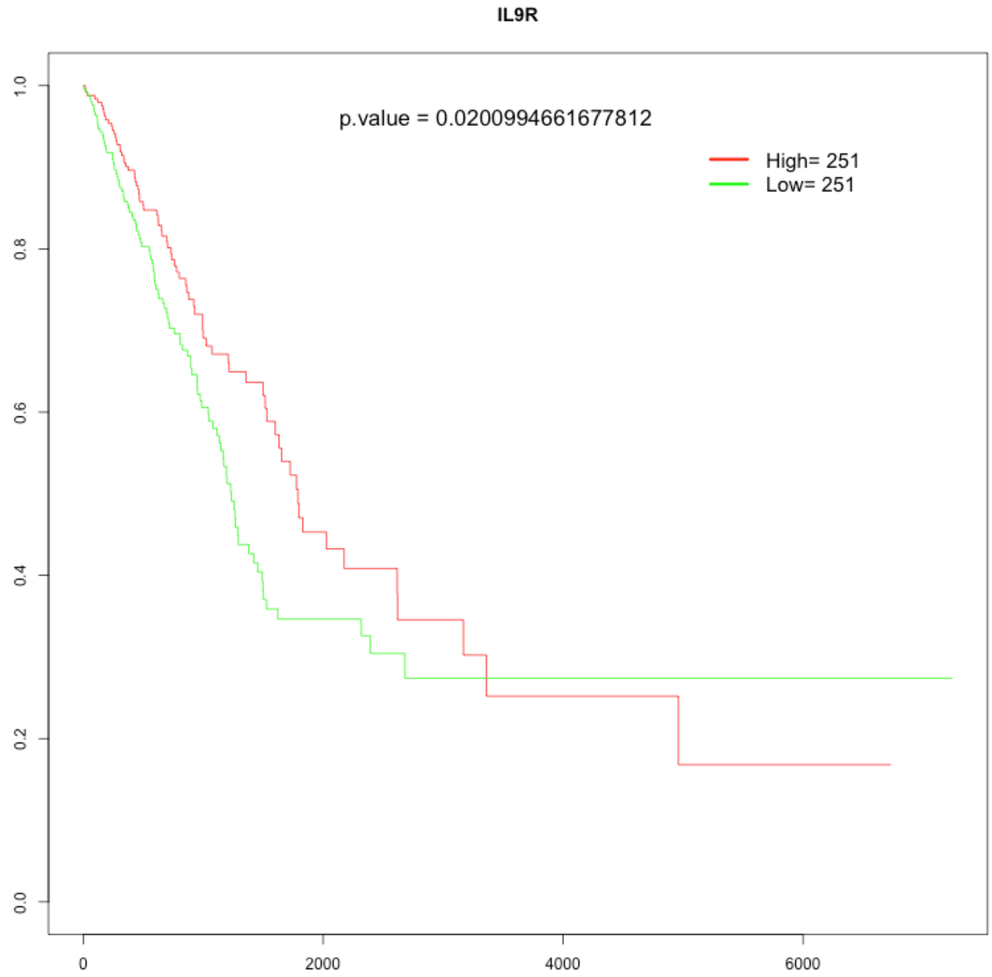
IL9R
p.value = 0.0200994661677812
High= 251
Low= 251
1.0
0.8
0.6
0.4
0.2
0.0
0
2000
4000
6000

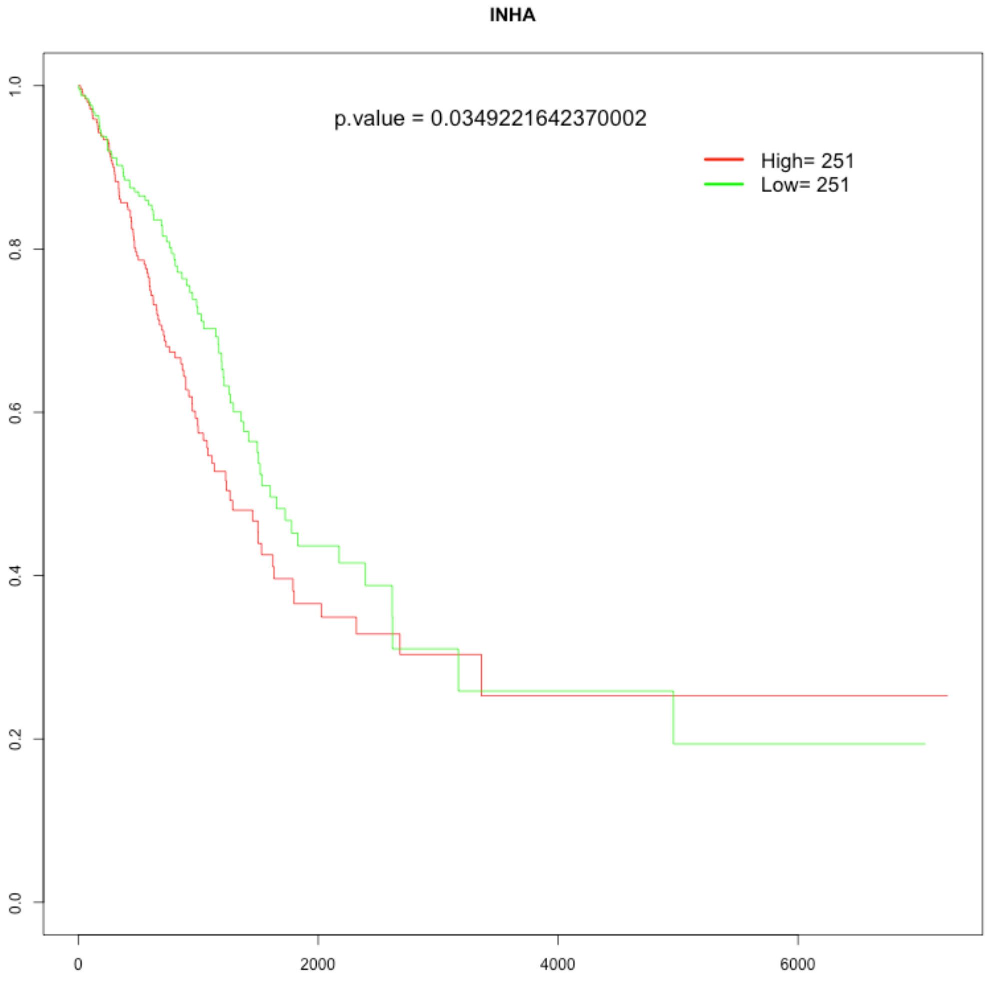
INHA
p.value = 0.0349221642370002
High= 251
Low= 251
1.0
0.8
0.6
0.4
0.2
0.0
0
2000
4000
6000

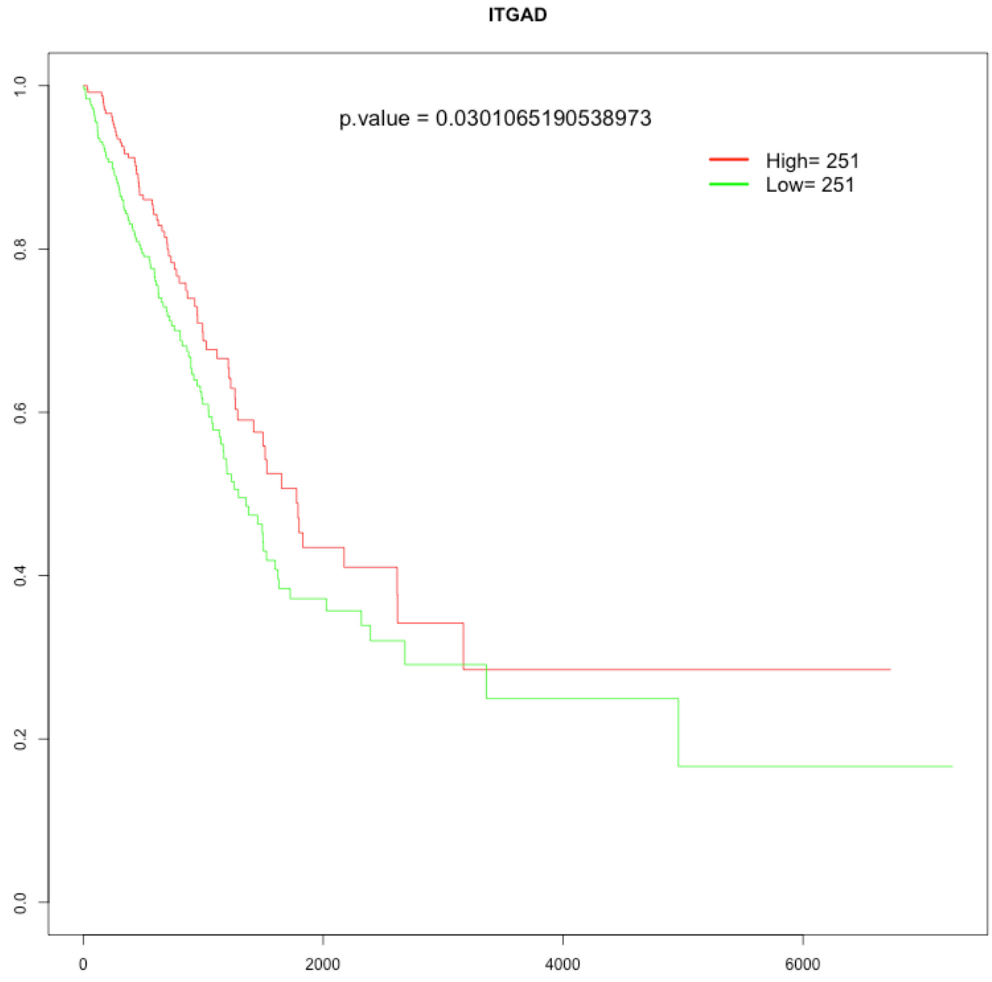

ITGAD
p.value = 0.0301065190538973
High= 251
Low= 251
1.0
0.8
0.6
0.4
0.2
0.0
0
2000
4000
6000

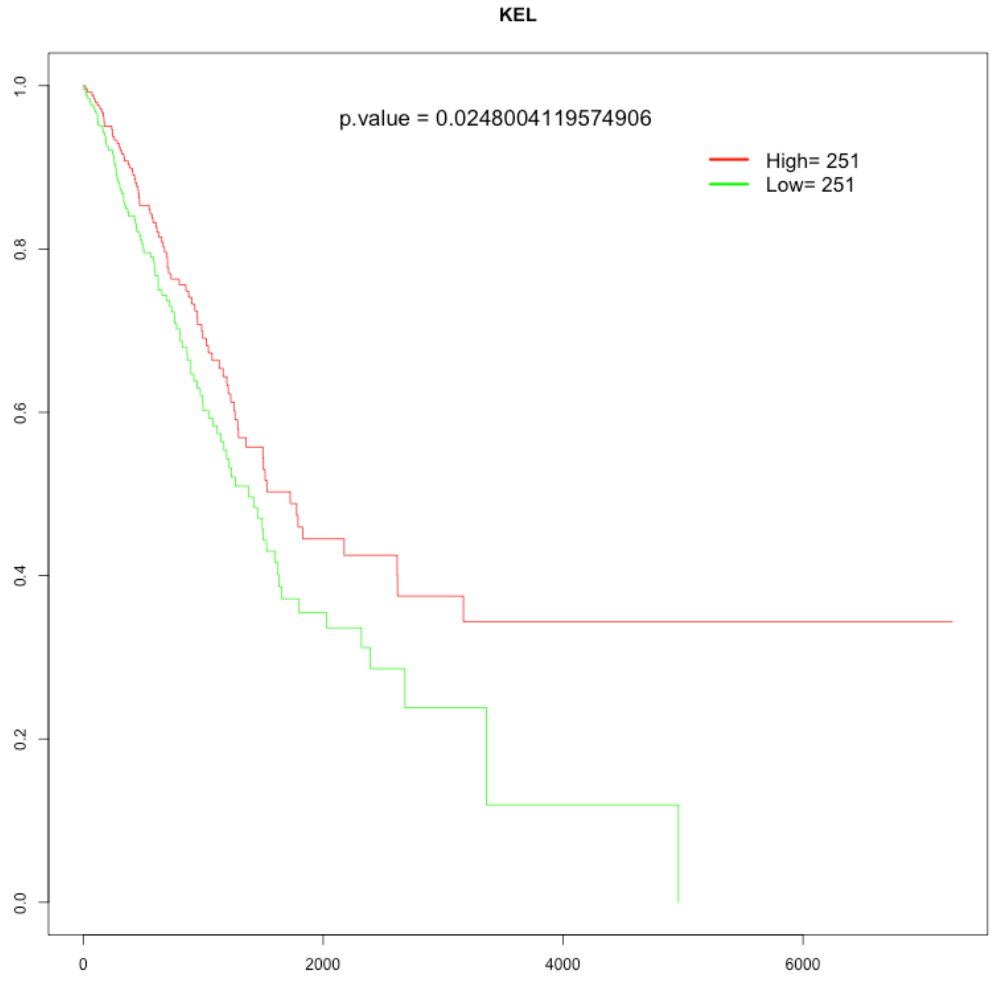

KEL
p.value = 0.0248004119574906
High= 251
Low= 251
1.0
0.8
0.6
0.4
0.2
0.0
0
2000
4000
6000

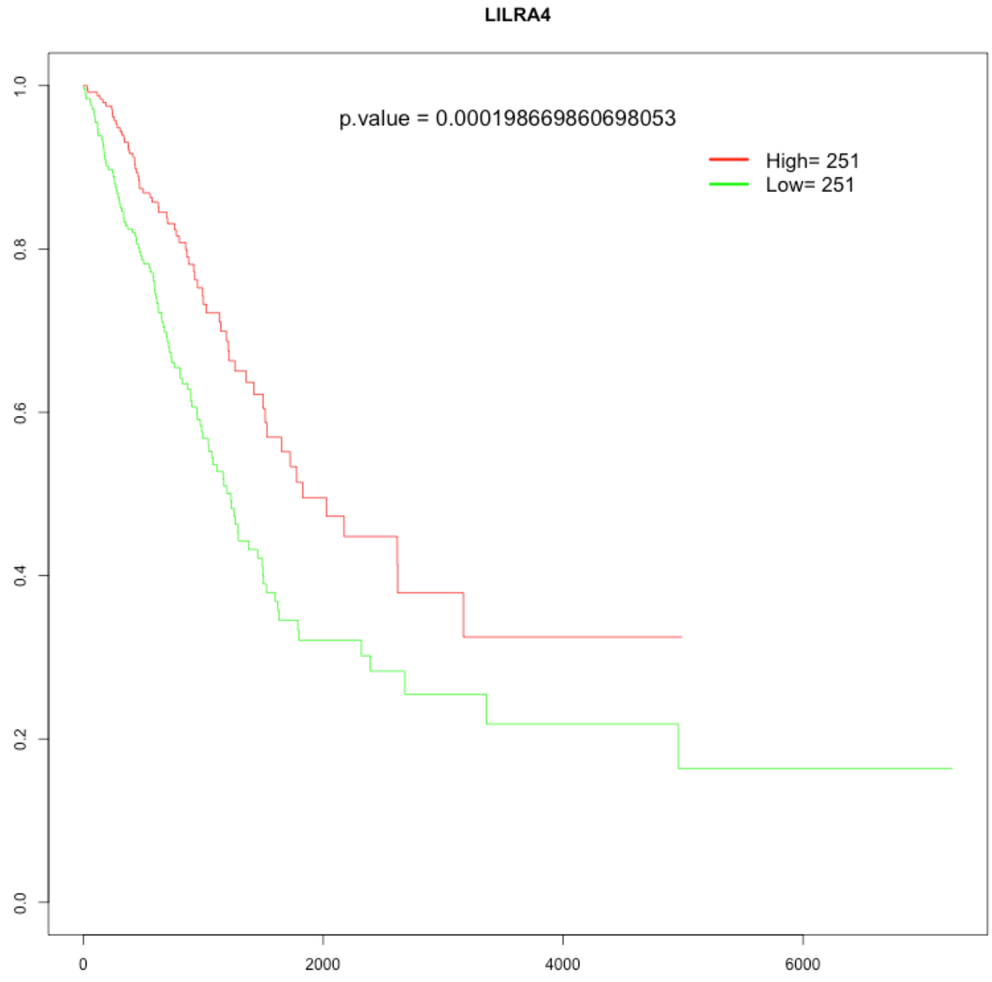
LILRA4
p.value = 0.000198669860698053
High= 251
Low= 251
1.0
0.8
0.6
0.4
0.2
0.0
0
2000
4000
6000

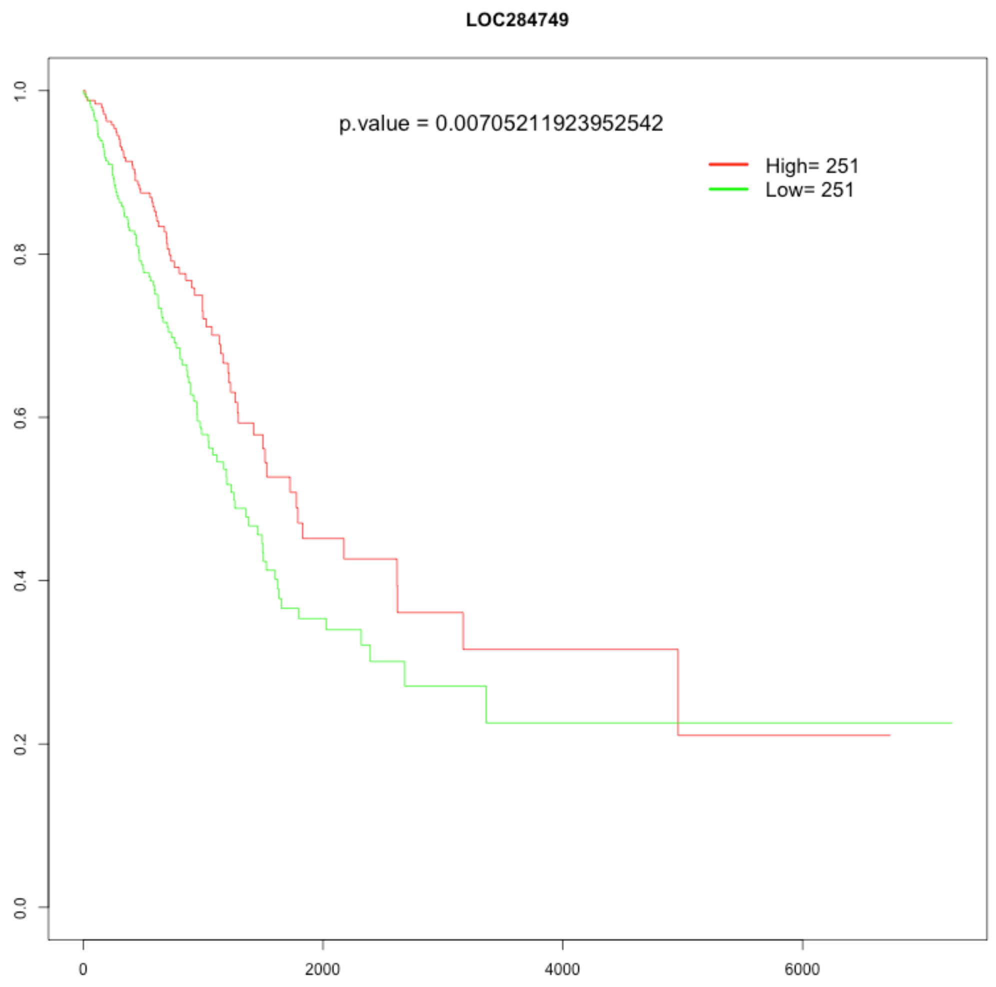

LOC284749
p.value = 0.00705211923952542
High= 251
Low= 251
1.0
0.8
0.6
0.4
0.2
0.0
0
2000
4000
6000

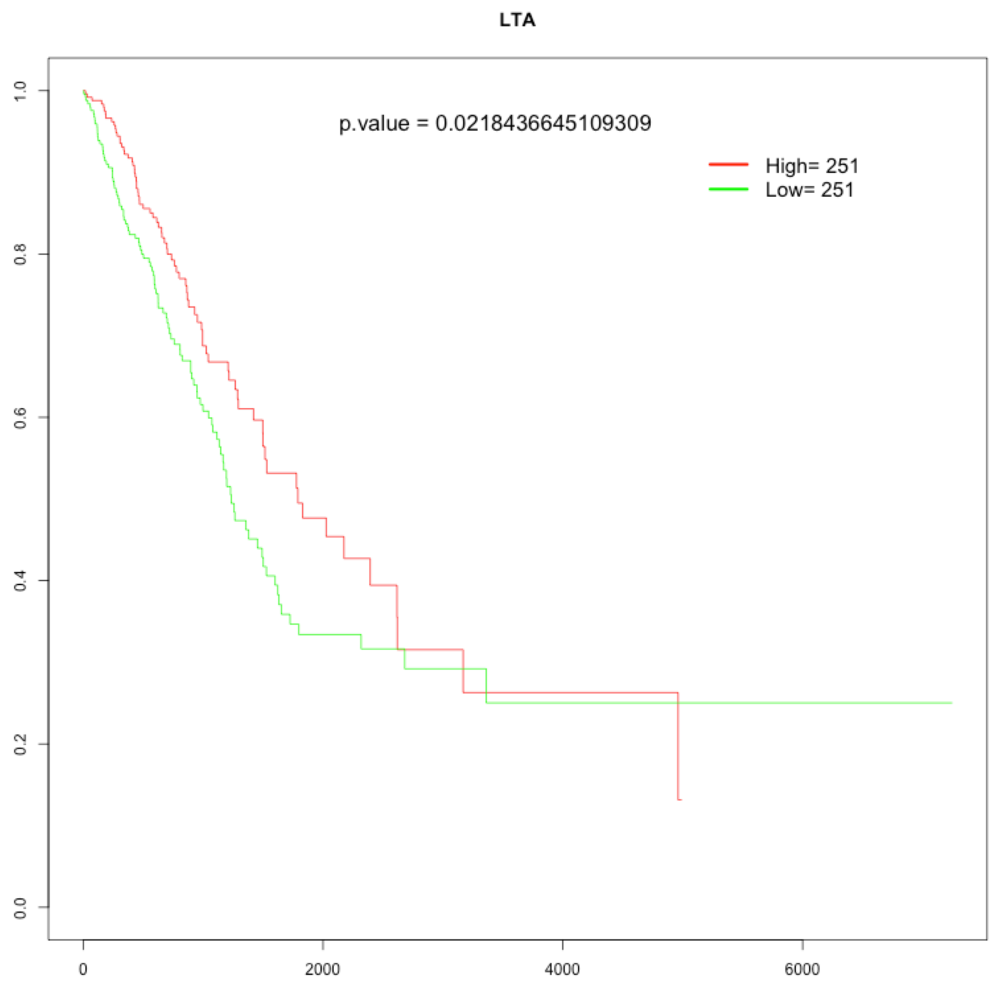
LTA
p.value = 0.0218436645109309
High= 251
Low= 251
1.0
0.8
0.6
0.4
0.2
0.0
0
2000
4000
6000

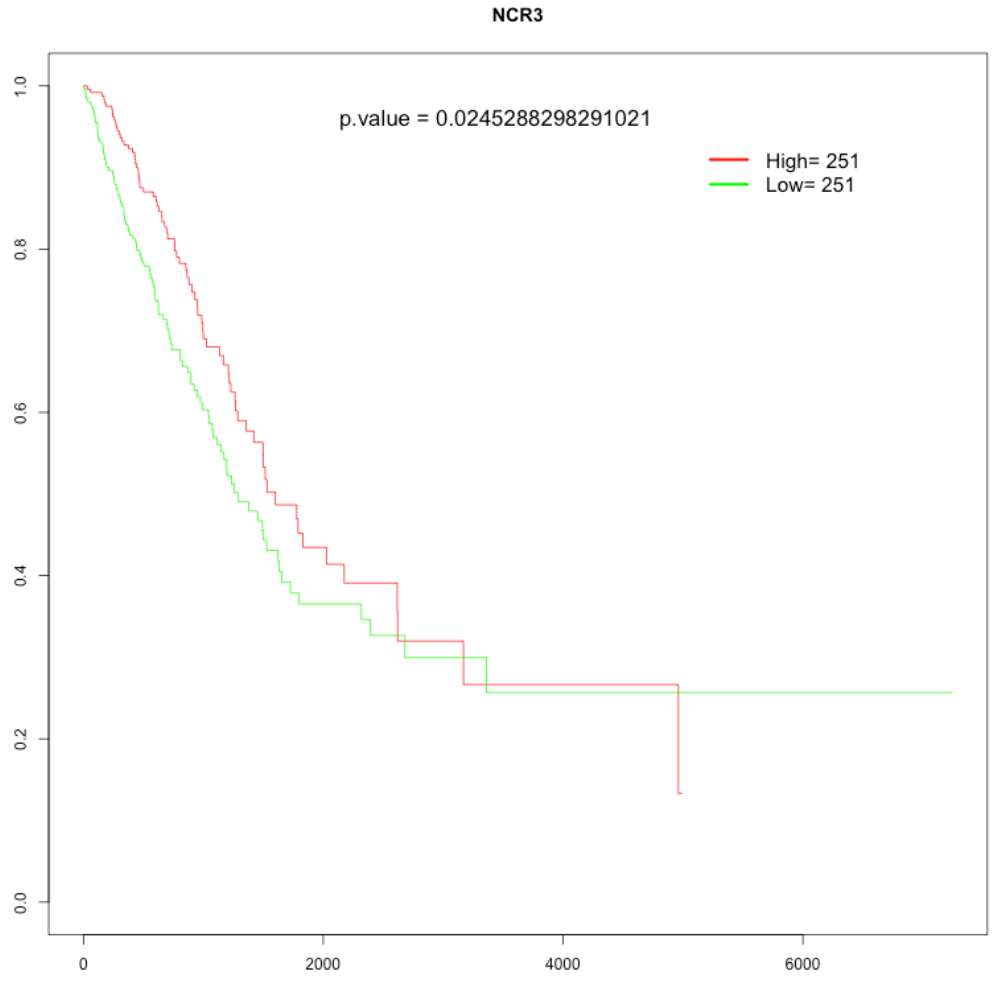
NCR3
p.value = 0.0245288298291021
High= 251
Low= 251
1.0
0.8
0.6
0.4
0.2
0.0
0
2000
4000
6000

P2RY12

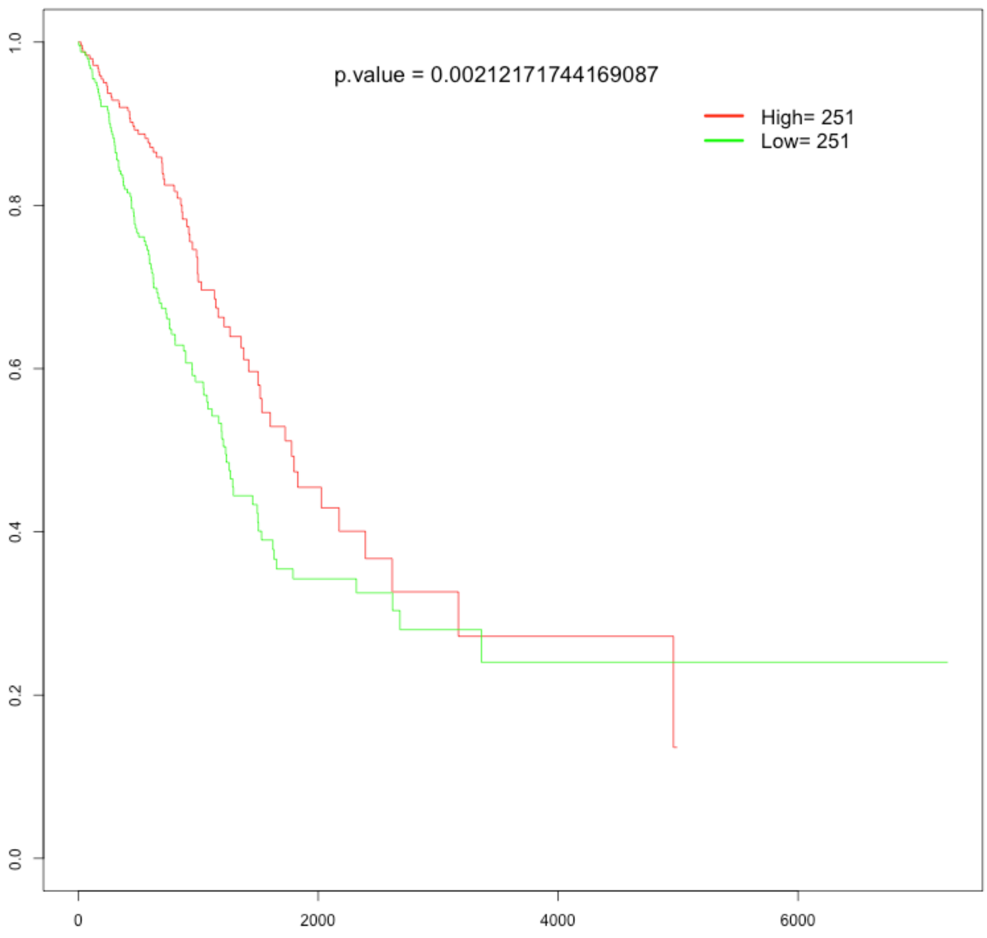

p.value = 0.00212171744169087
High= 251
Low= 251
1.0
0.8
0.6
0.4
0.2
0.0
0
2000
4000
6000

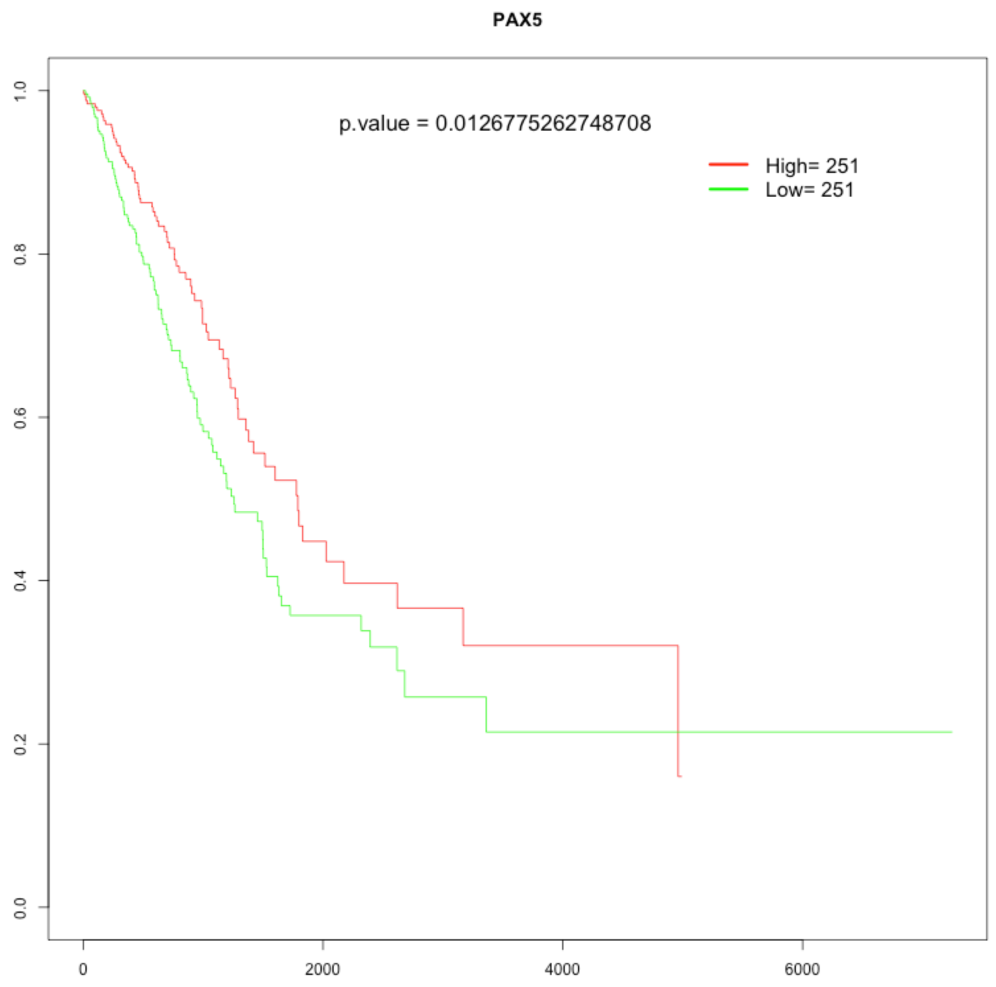

PAX5
p.value = 0.0126775262748708
High= 251
Low= 251
1.0
0.8
0.6
0.4
0.2
0.0
0
2000
4000
6000

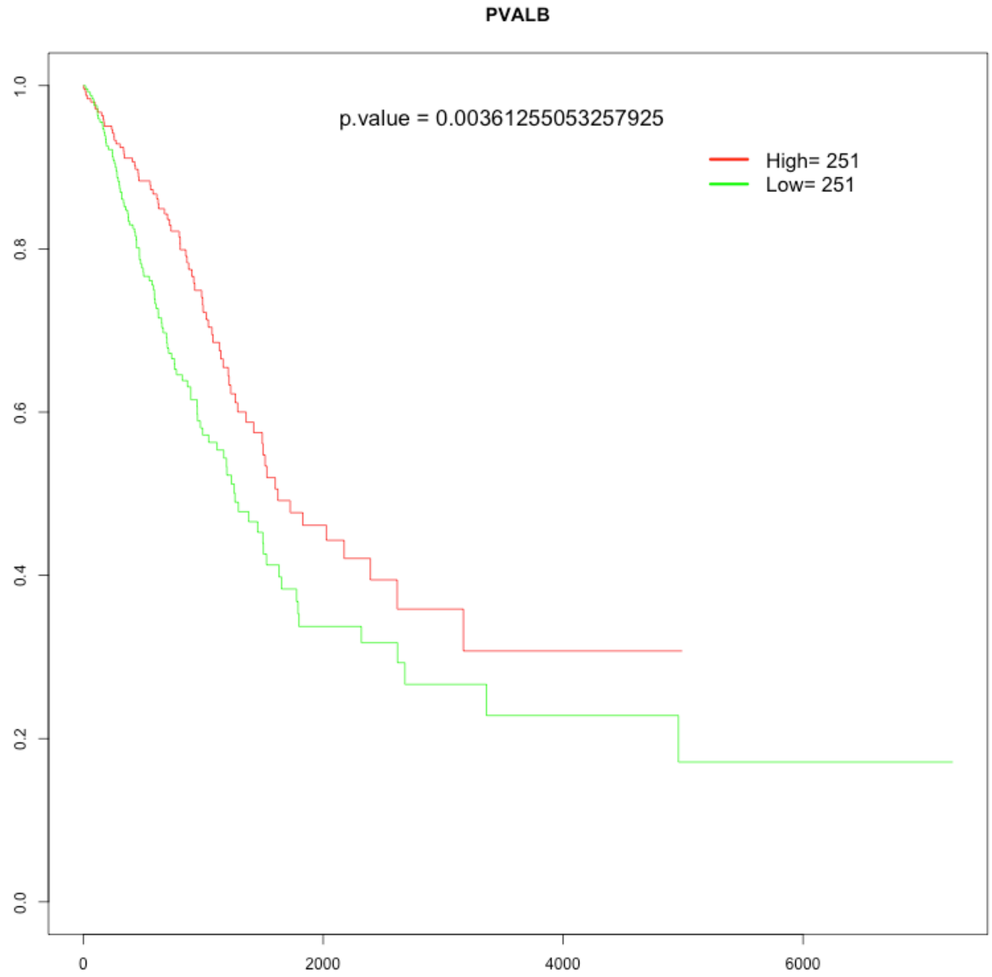
PVALB
p.value = 0.00361255053257925
High= 251
Low= 251
0.0
0.2
0.4
0.6
0.8
1.0
0
2000
4000
6000

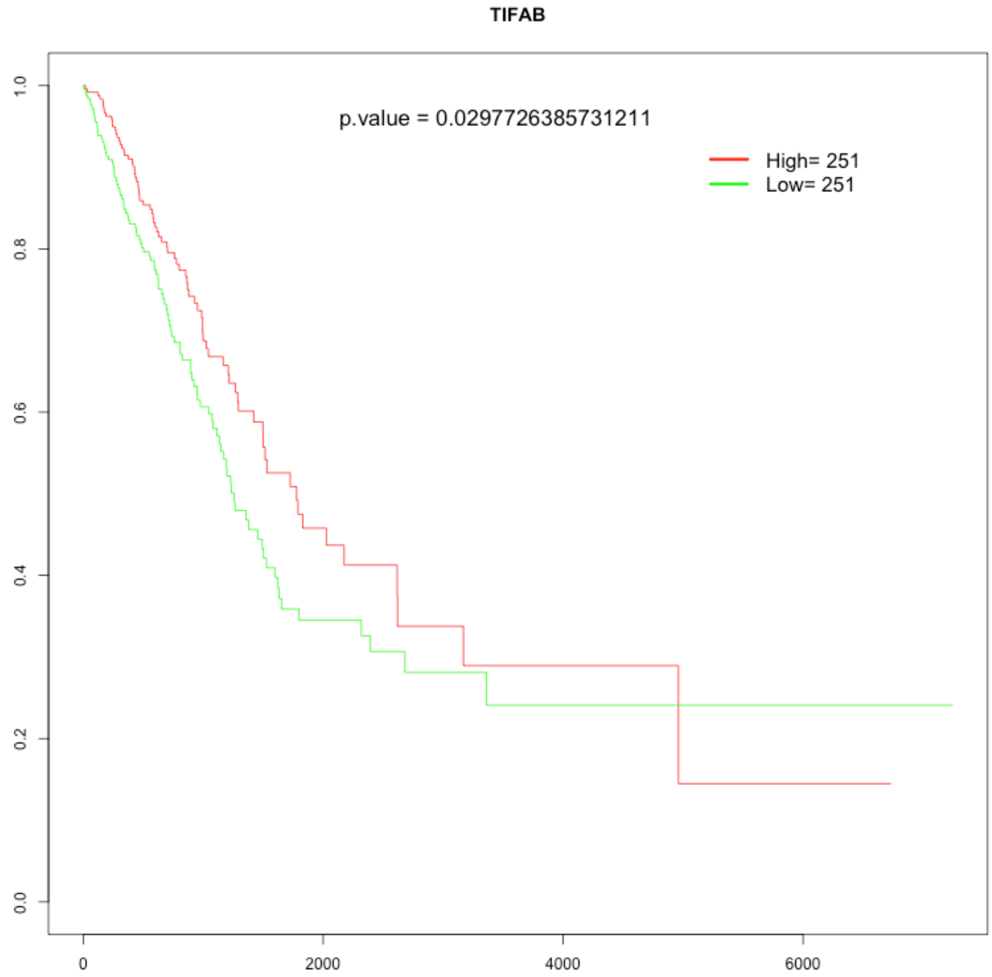
TIFAB
p.value = 0.0297726385731211
High= 251
Low= 251
0.0
0.2
0.4
0.6
0.8
1.0
0
2000
4000
6000

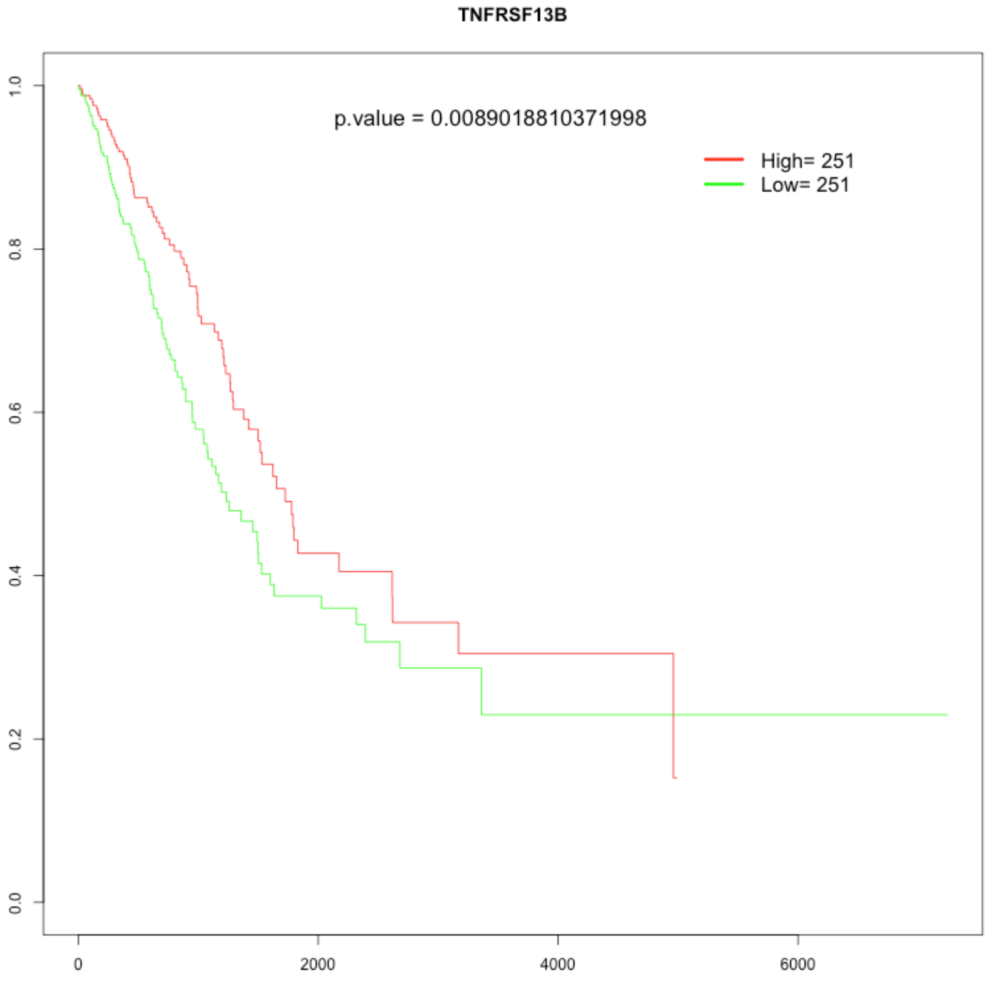

TNFRSF13B
p.value = 0.0089018810371998
High= 251
Low= 251
0.0
0.2
0.4
0.6
0.8
1.0
0
2000
4000
6000

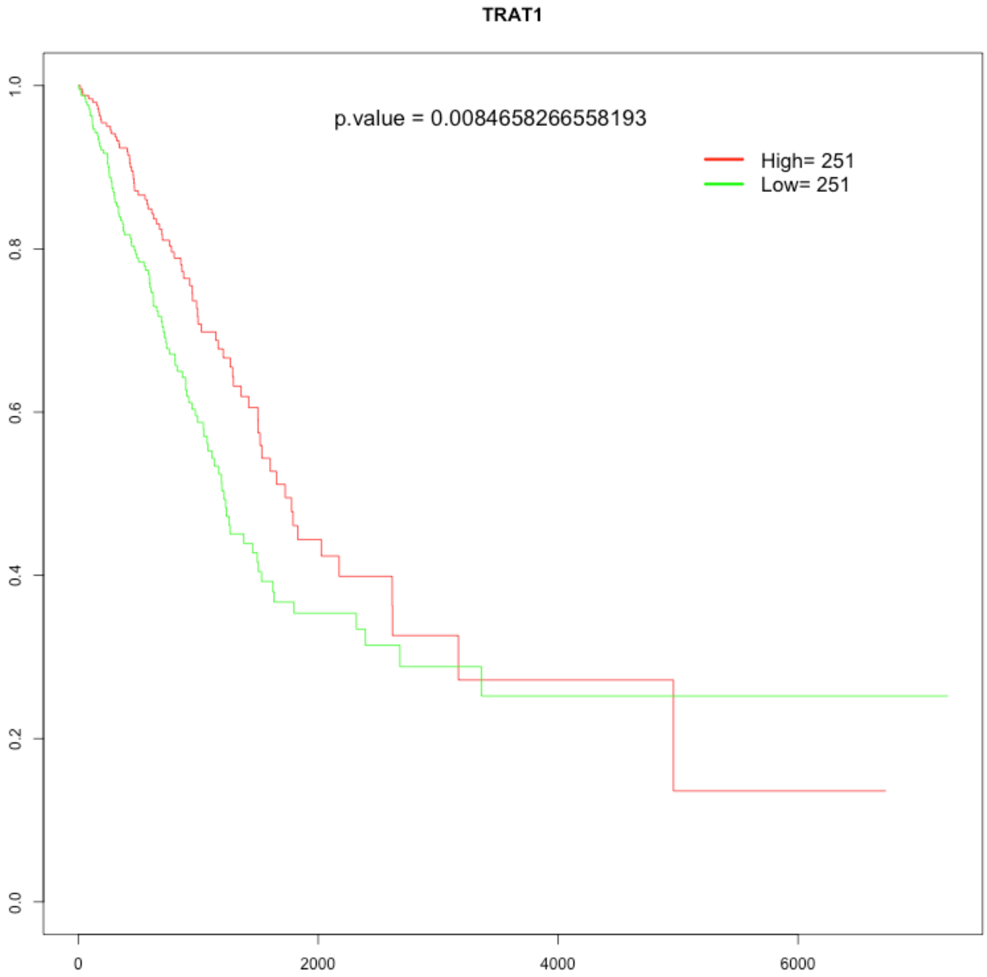

TRAT1
p.value = 0.0084658266558193
High= 251
Low= 251
1.0
0.8
0.6
0.4
0.2
0.0
0
2000
4000
6000

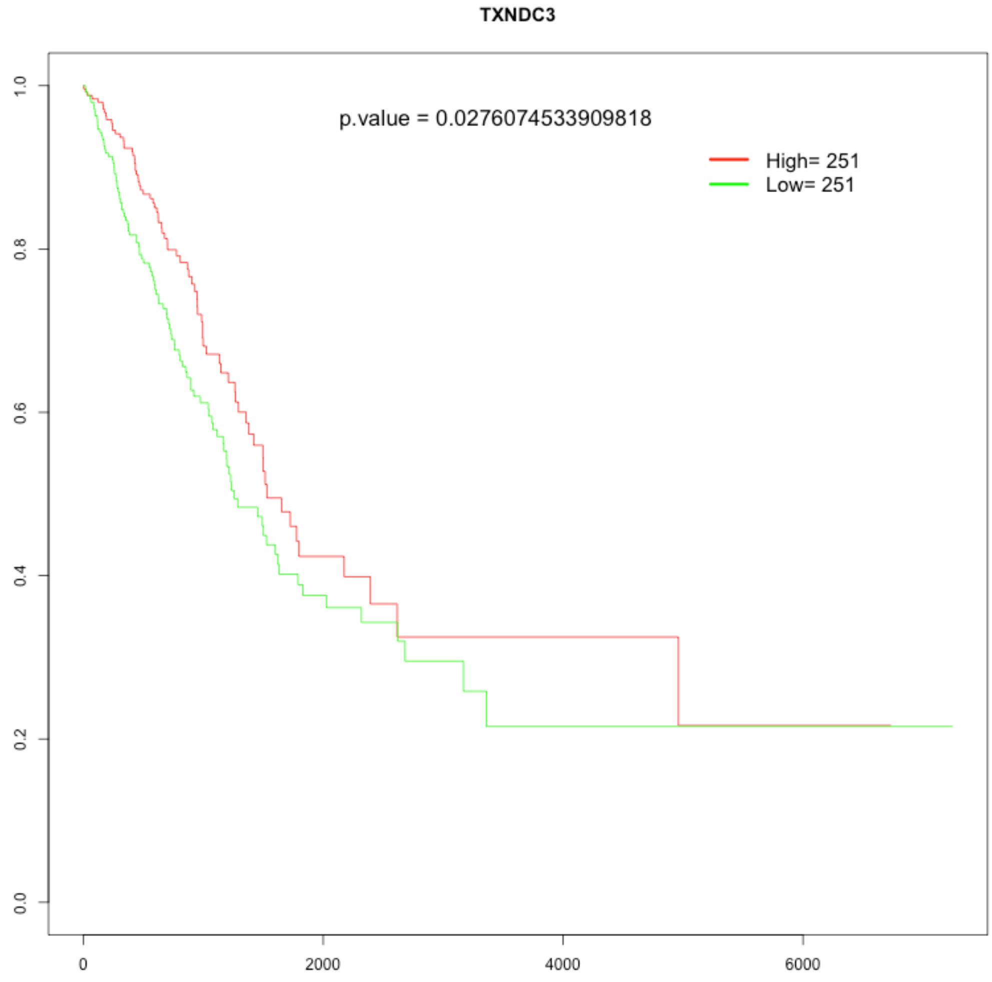
TXNDC3
p.value = 0.0276074533909818
High= 251
Low= 251
1.0
0.8
0.6
0.4
0.2
0.0
0
2000
4000
6000

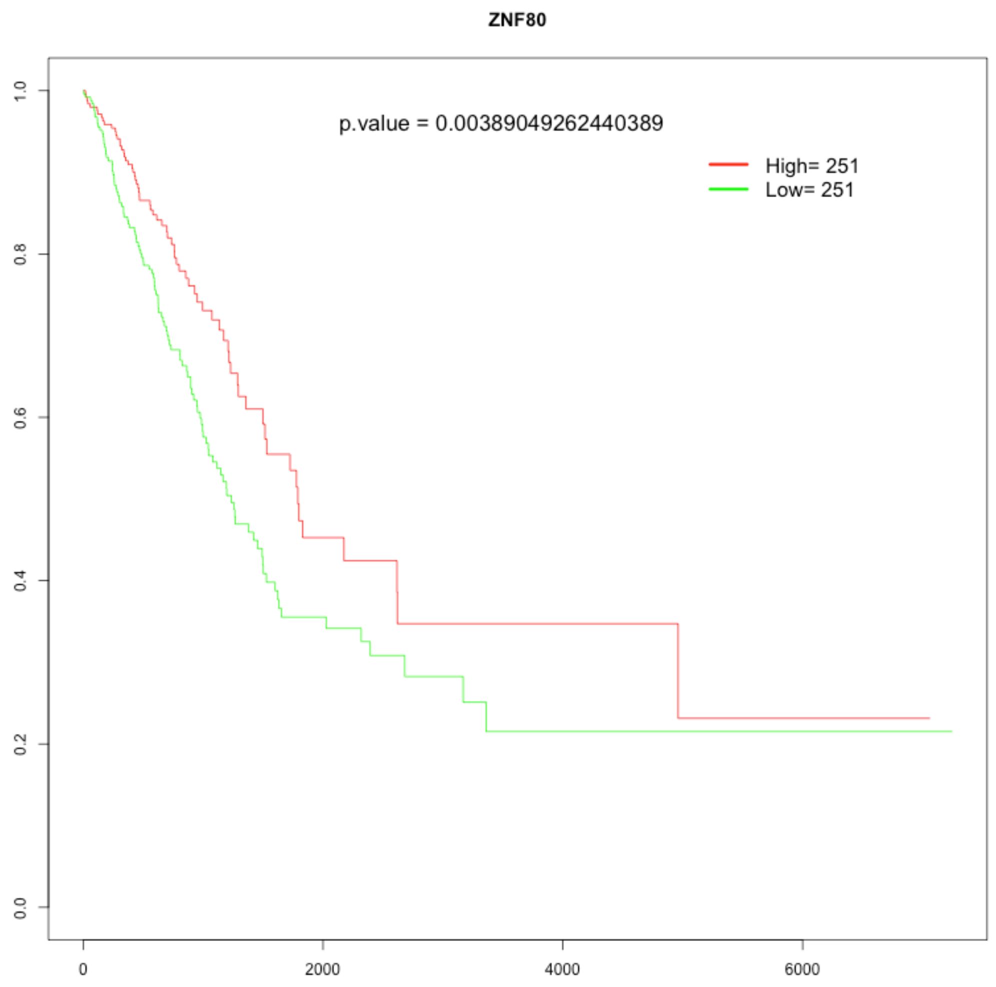

ZNF80
p.value = 0.00389049262440389
High= 251
Low= 251
1.0
0.8
0.6
0.4
0.2
0.0
0
2000
4000
6000

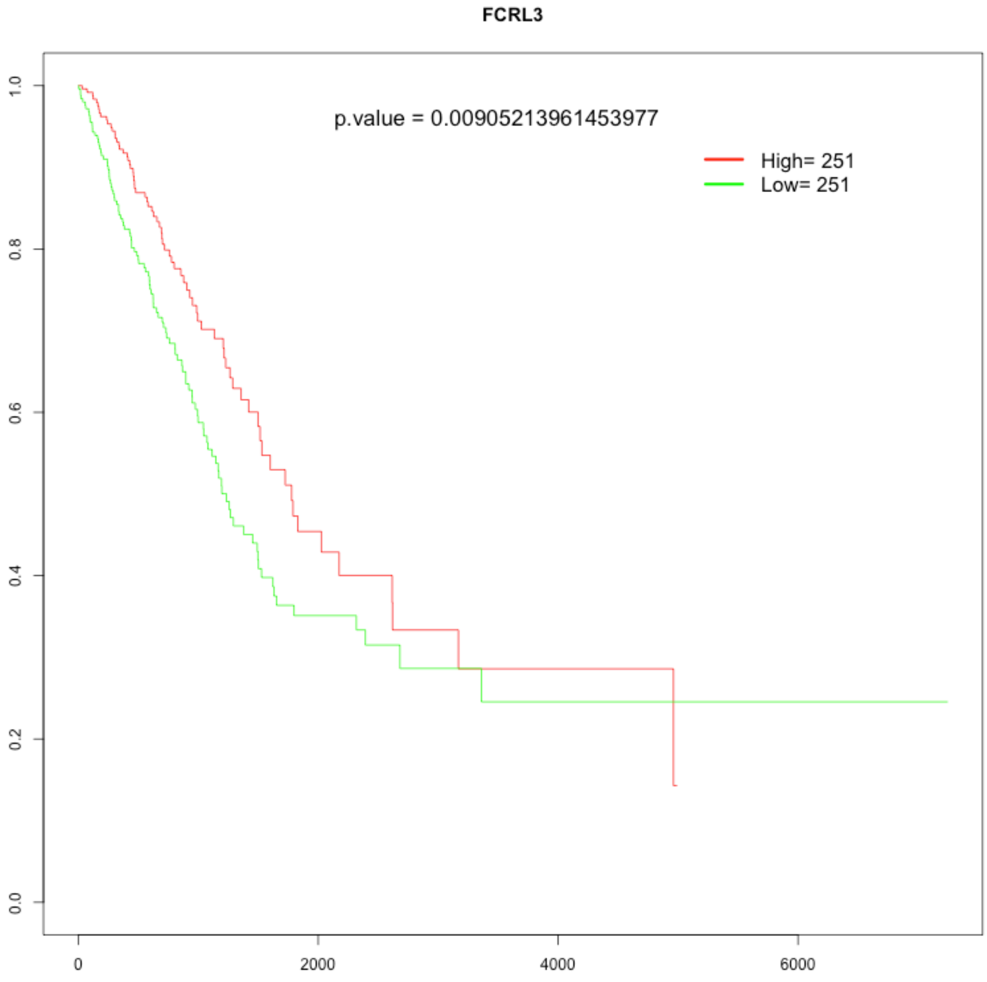

FCRL3
p.value = 0.00905213961453977
High= 251
Low= 251
1.0
0.8
0.6
0.4
0.2
0.0
0
2000
4000
6000

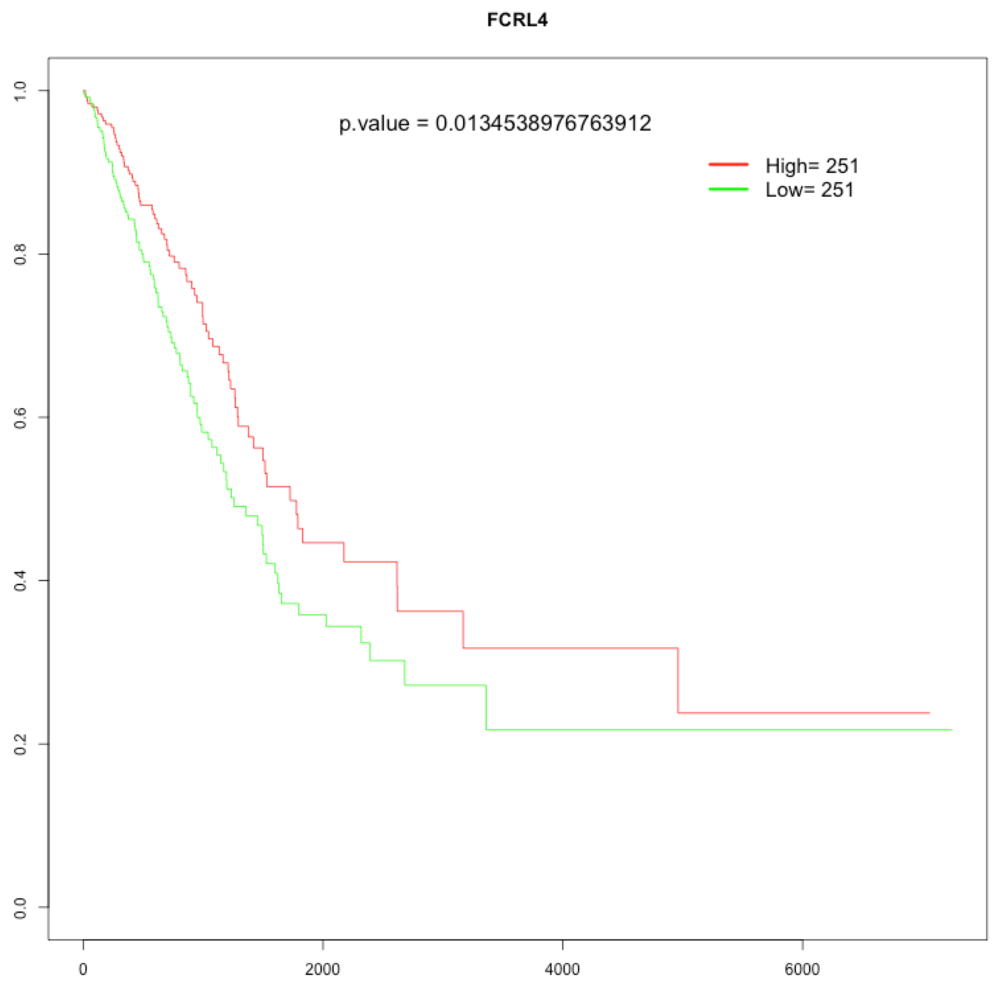

FCRL4
p.value = 0.0134538976763912
High= 251
Low= 251
1.0
0.8
0.6
0.4
0.2
0.0
0
2000
4000
6000

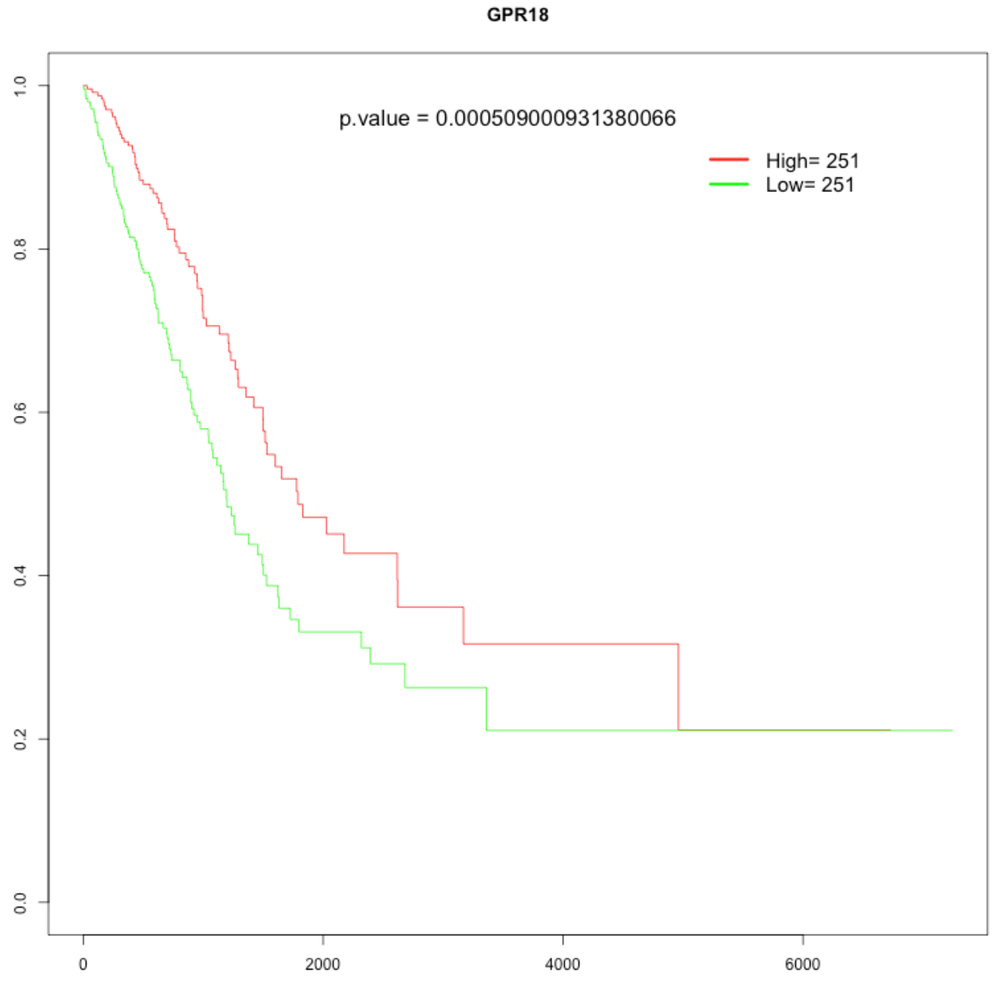

GPR18
p.value = 0.000509000931380066
High= 251
Low= 251
1.0
0.8
0.6
0.4
0.2
0.0
0
2000
4000
6000

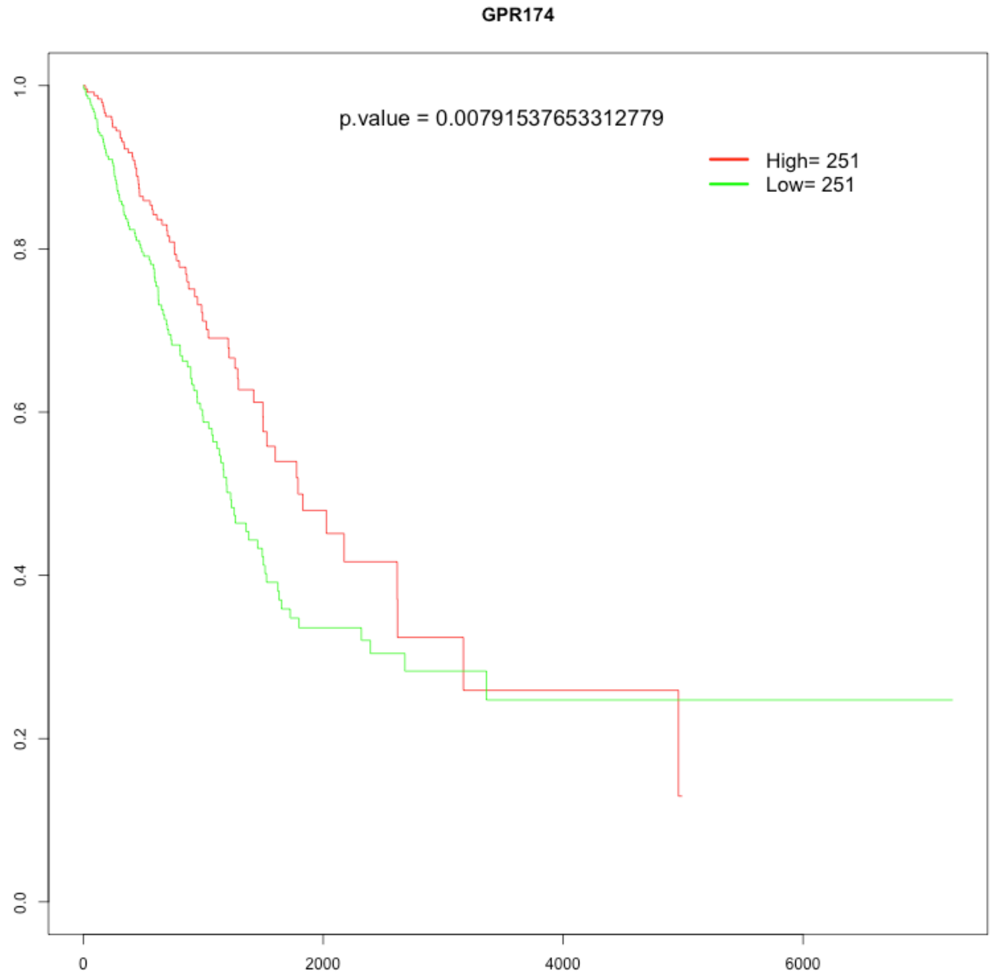
GPR174
p.value = 0.00791537653312779
High= 251
Low= 251
1.0
0.8
0.6
0.4
0.2
0.0
0
2000
4000
6000

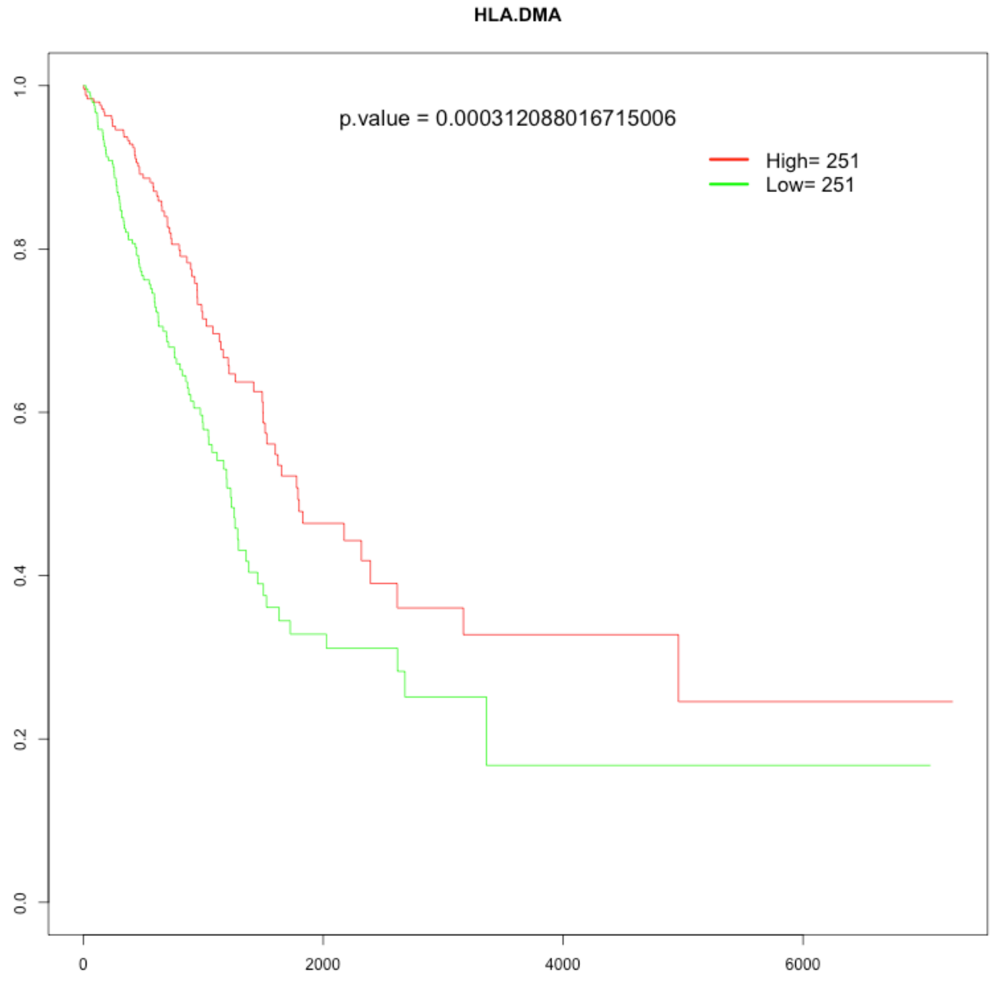
HLA.DMA
p.value = 0.000312088016715006
High= 251
Low= 251
0.0
0.2
0.4
0.6
0.8
1.0
0
2000
4000
6000

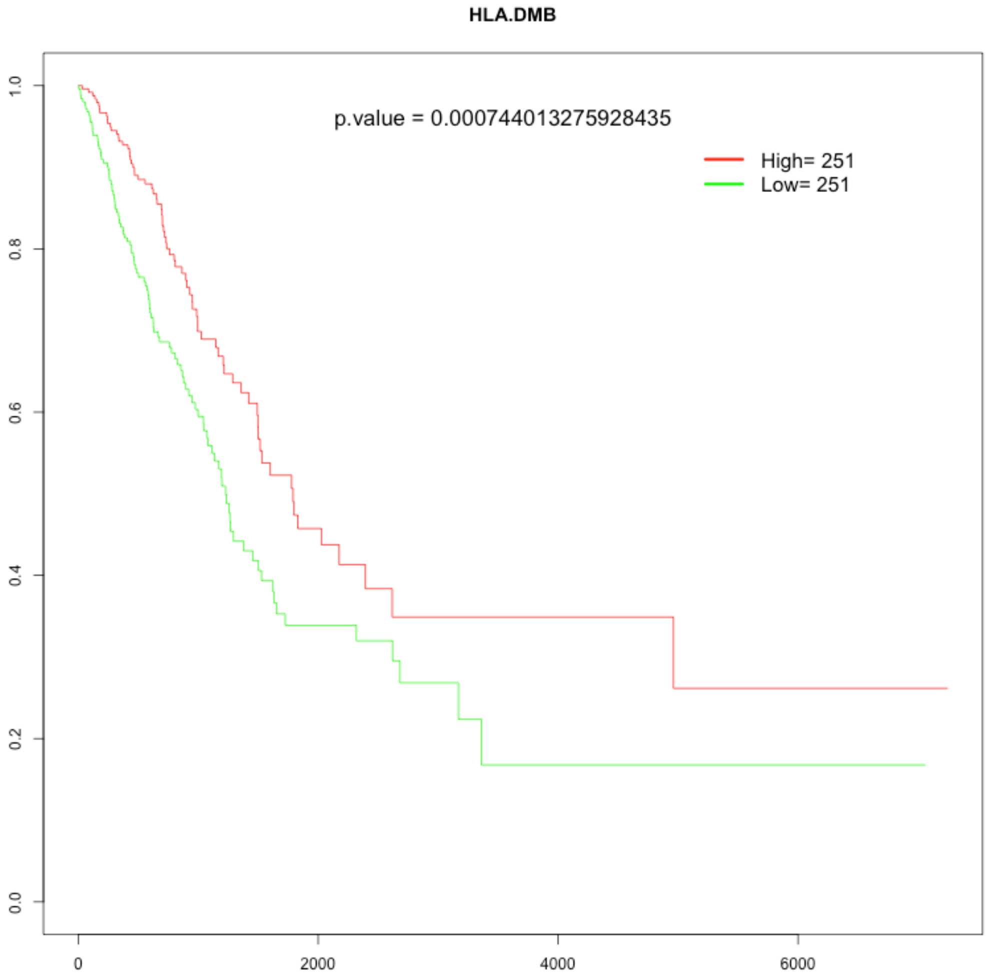

HLA.DMB
p.value = 0.000744013275928435
High= 251
Low= 251
1.0
0.8
0.6
0.4
0.2
0.0
0
2000
4000
6000

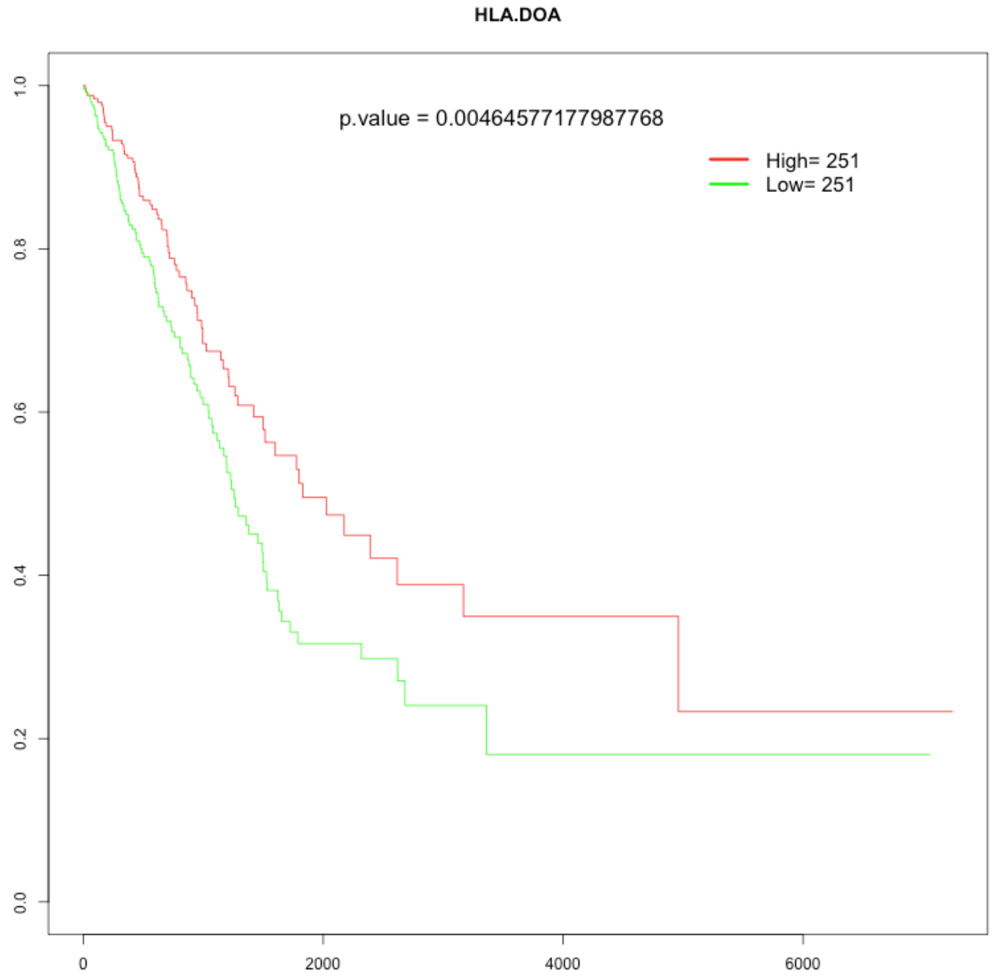

HLA.DOA
p.value = 0.00464577177987768
High= 251
Low= 251
1.0
0.8
0.6
0.4
0.2
0.0
0
2000
4000
6000

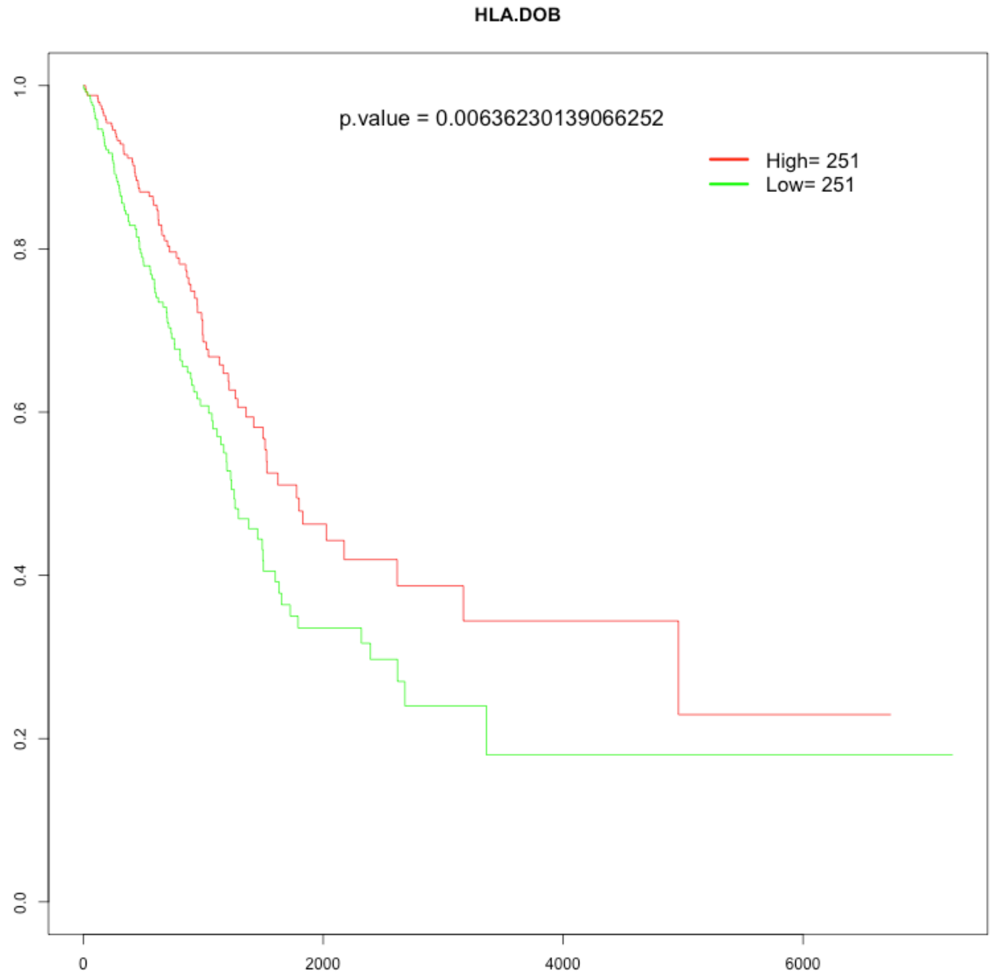
HLA.DOB
p.value = 0.00636230139066252
High= 251
Low= 251
1.0
0.8
0.6
0.4
0.2
0.0
0
2000
4000
6000

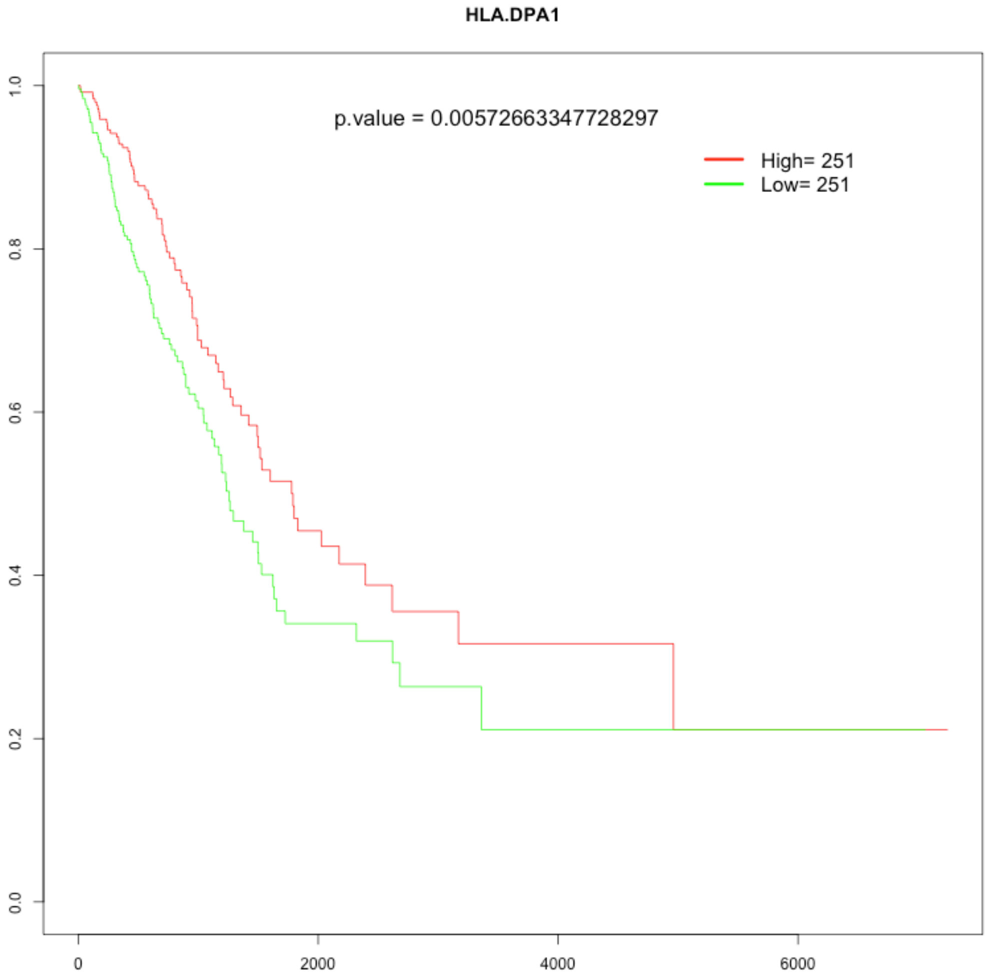
HLA.DPA1
p.value = 0.00572663347728297
High= 251
Low= 251
1.0
0.8
0.6
0.4
0.2
0.0
0
2000
4000
6000

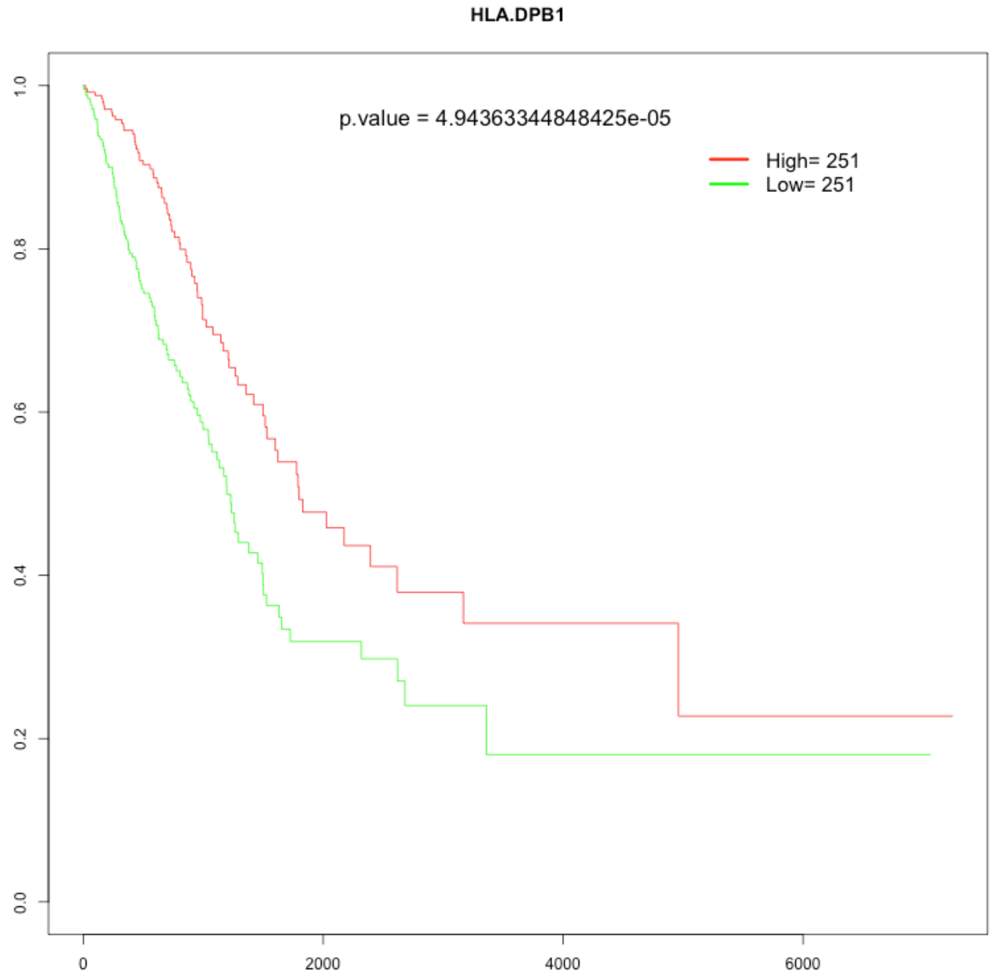
HLA.DPB1
p.value = 4.94363344848425e-05
High= 251
Low= 251
1.0
0.8
0.6
0.4
0.2
0.0
0
2000
4000
6000

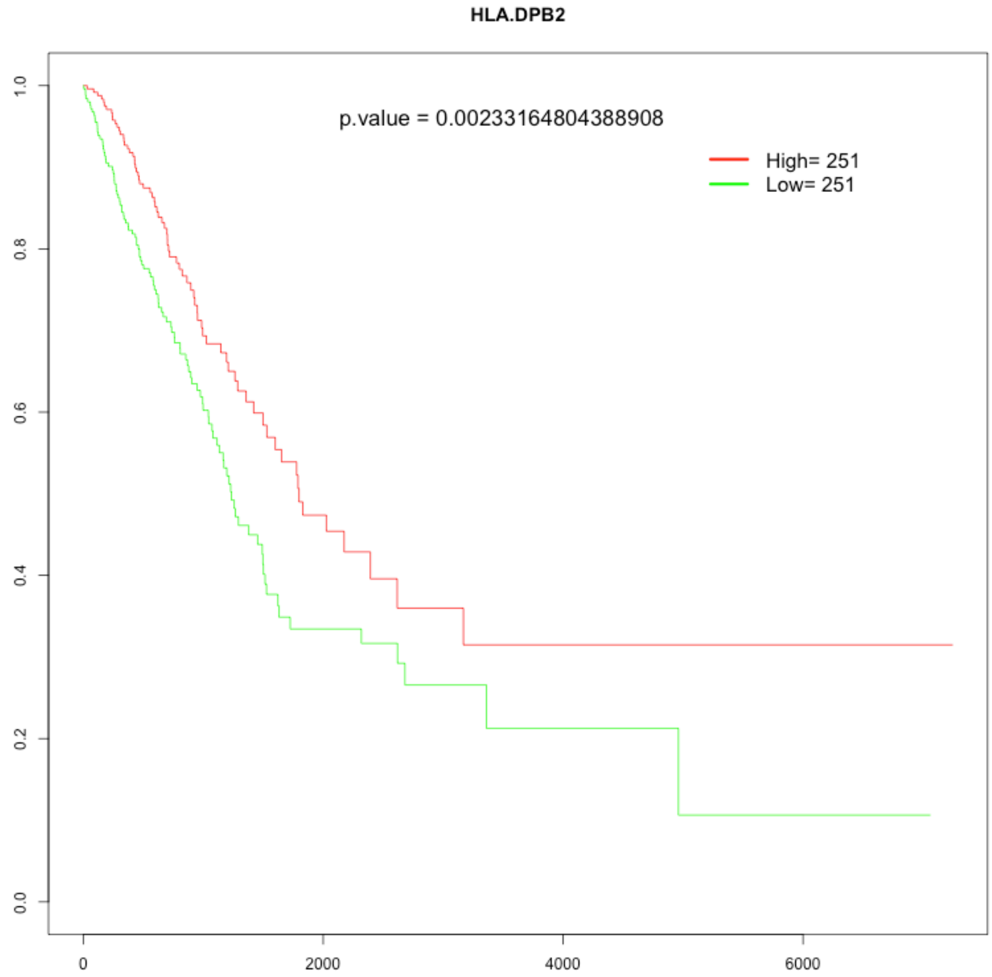
HLA.DPB2
p.value = 0.00233164804388908
High= 251
Low= 251
1.0
0.8
0.6
0.4
0.2
0.0
0
2000
4000
6000

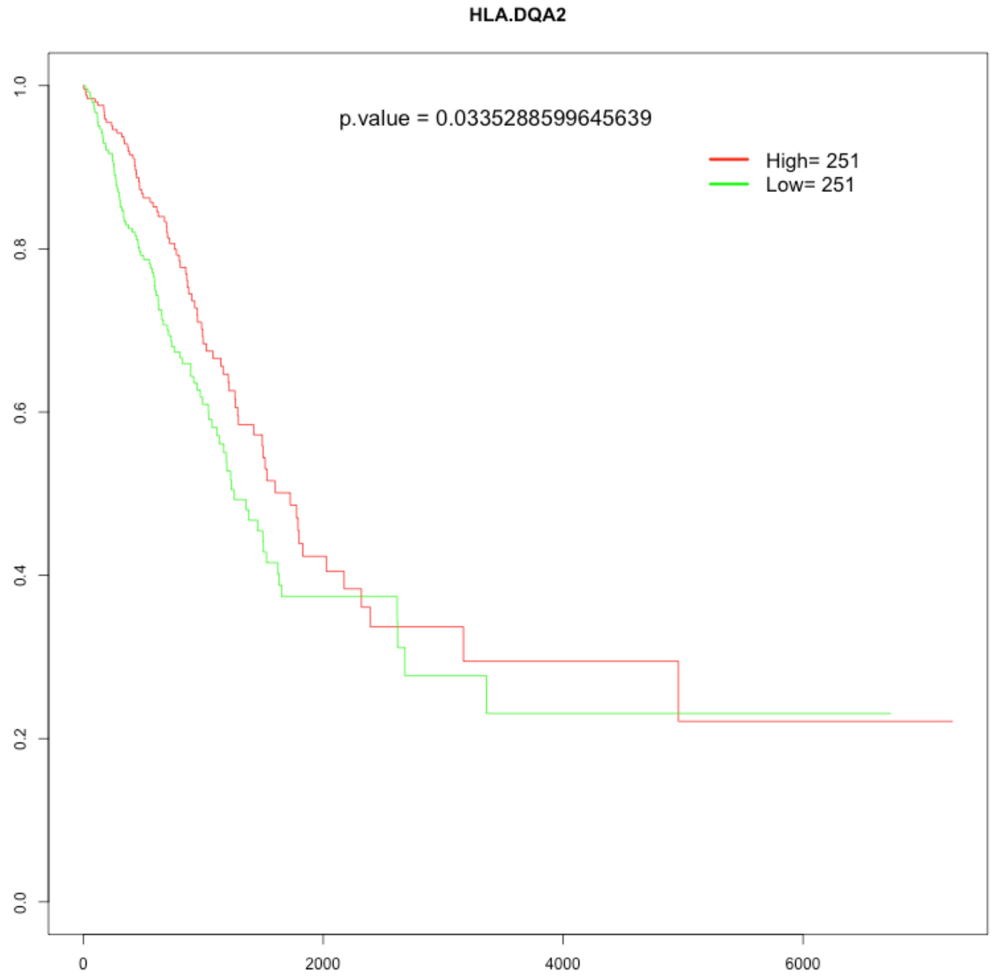

HLA.DQA2
p.value = 0.0335288599645639
High= 251
Low= 251
1.0
0.8
0.6
0.4
0.2
0.0
0
2000
4000
6000

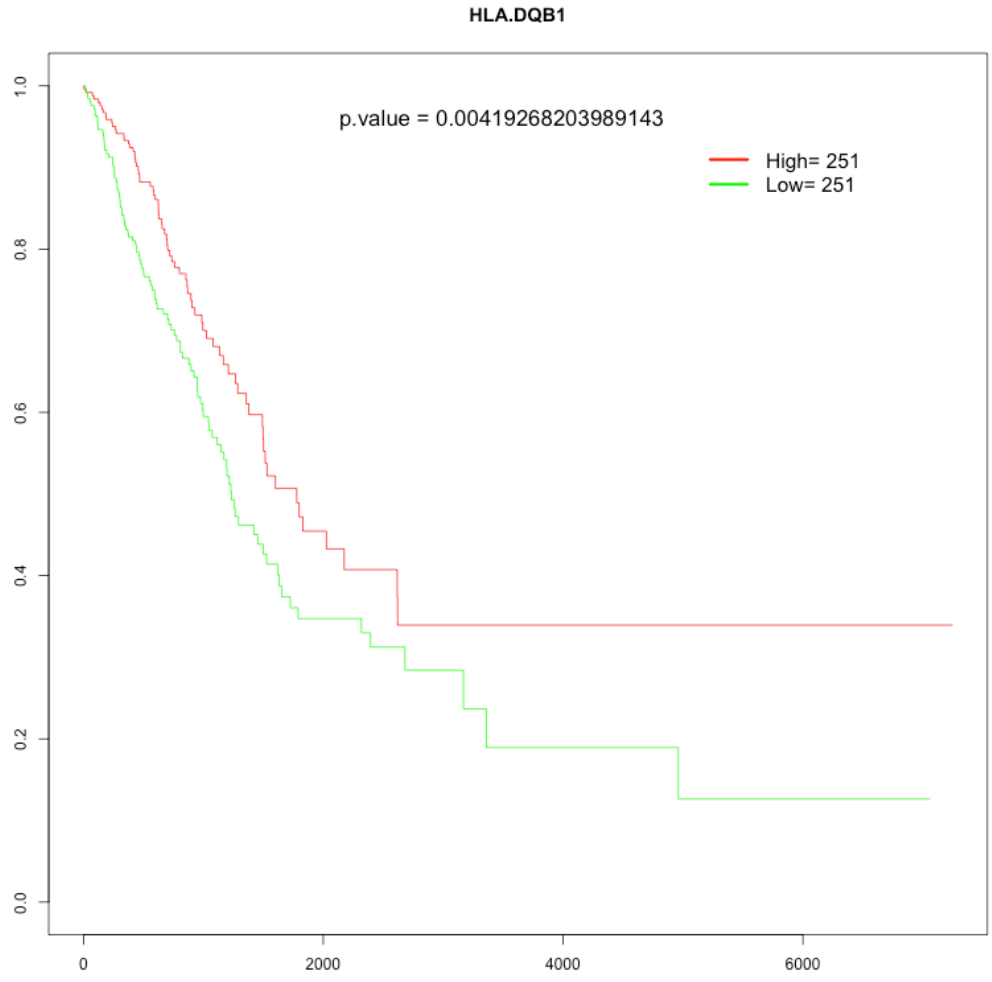
HLA.DQB1
p.value = 0.00419268203989143
High= 251
Low= 251
0.0
0.2
0.4
0.6
0.8
1.0
0
2000
4000
6000

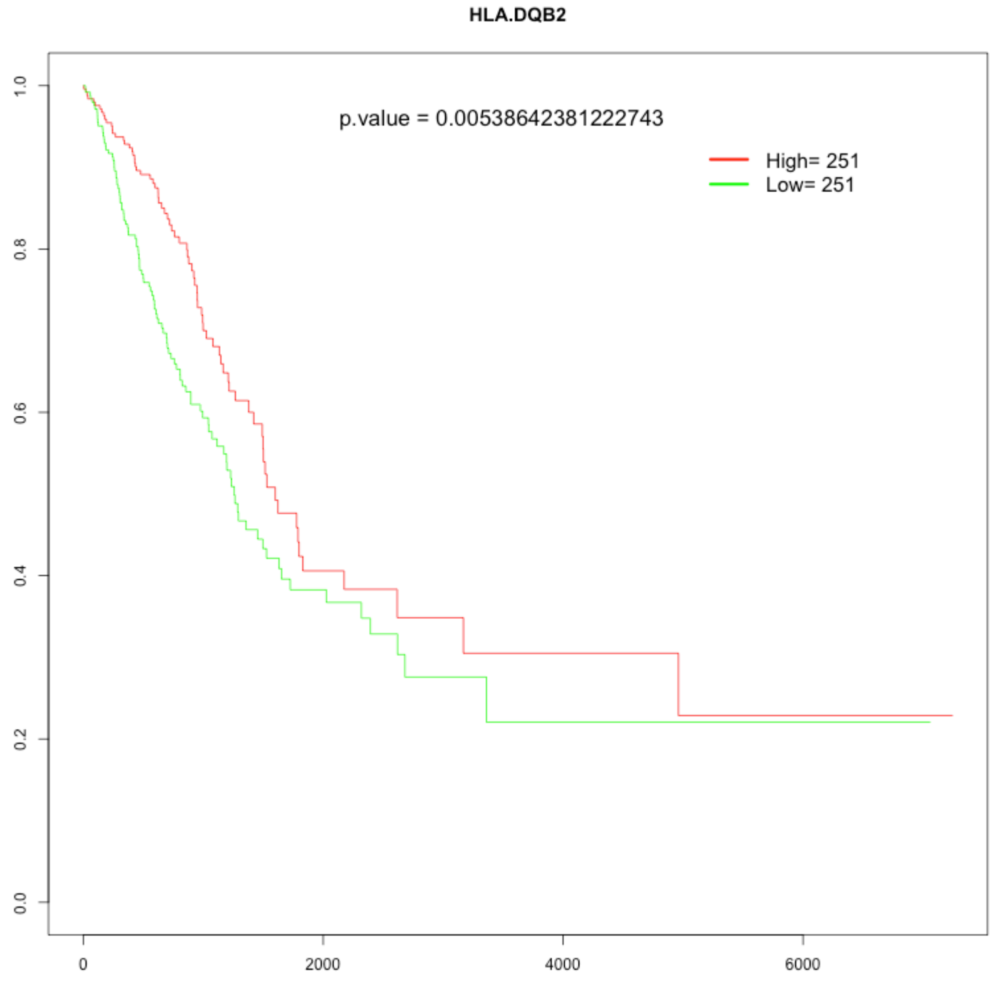

HLA.DQB2
p.value = 0.00538642381222743
High= 251
Low= 251
1.0
0.8
0.6
0.4
0.2
0.0
0
2000
4000
6000

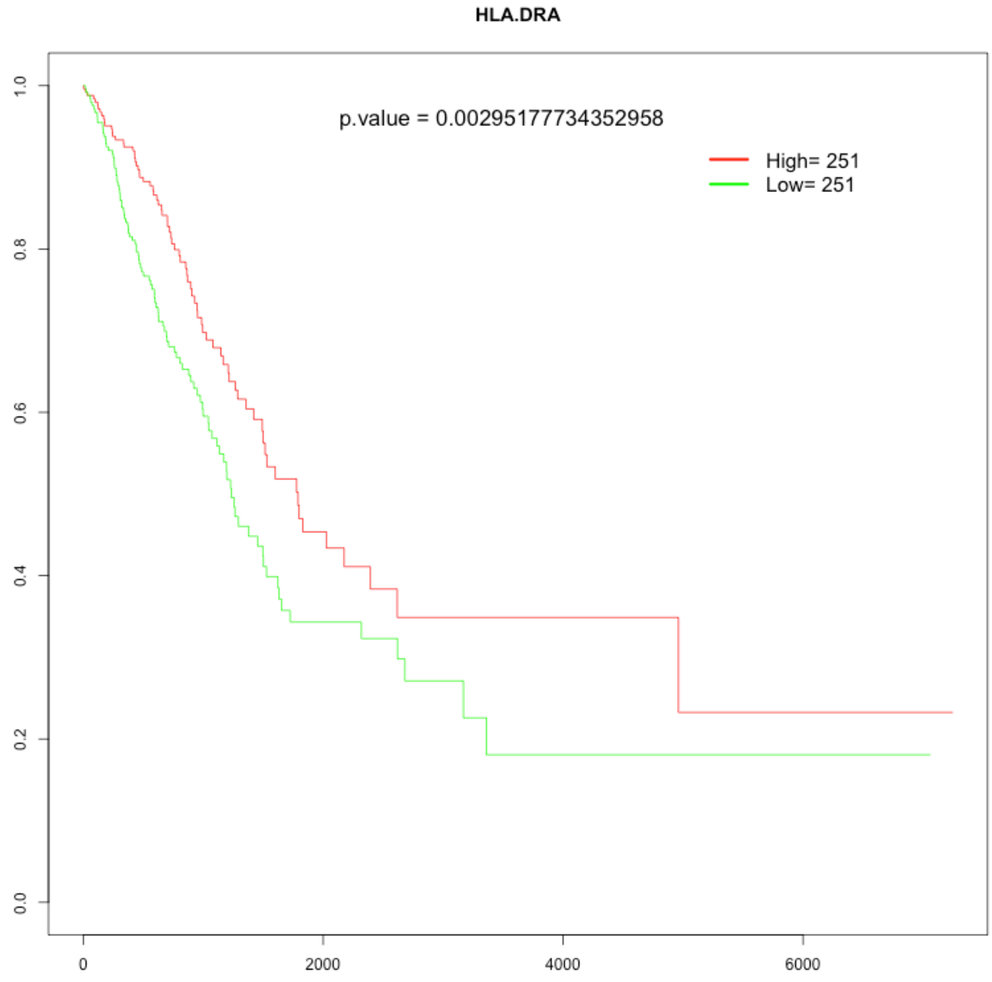

HLA.DRA
p.value = 0.00295177734352958
High= 251
Low= 251
1.0
0.8
0.6
0.4
0.2
0.0
0
2000
4000
6000

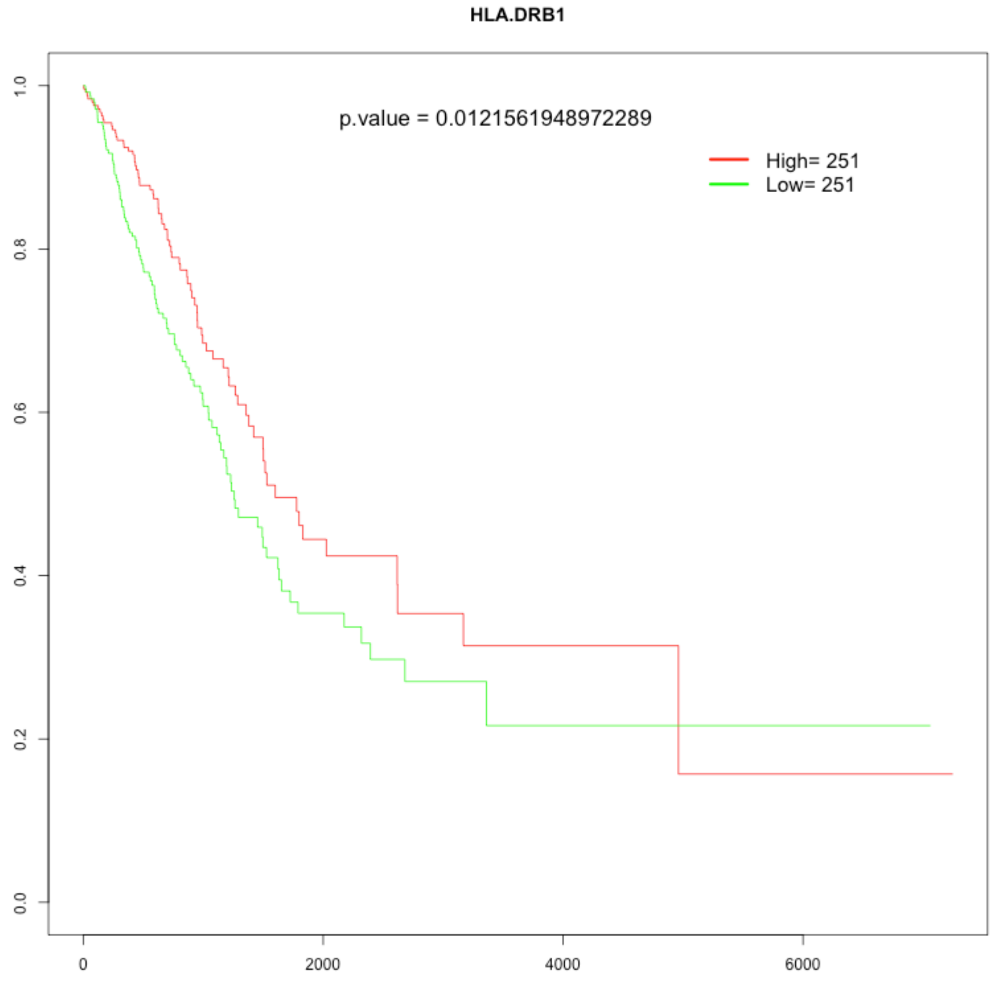
HLA.DRB1
p.value = 0.0121561948972289
High= 251
Low= 251
1.0
0.8
0.6
0.4
0.2
0.0
0
2000
4000
6000

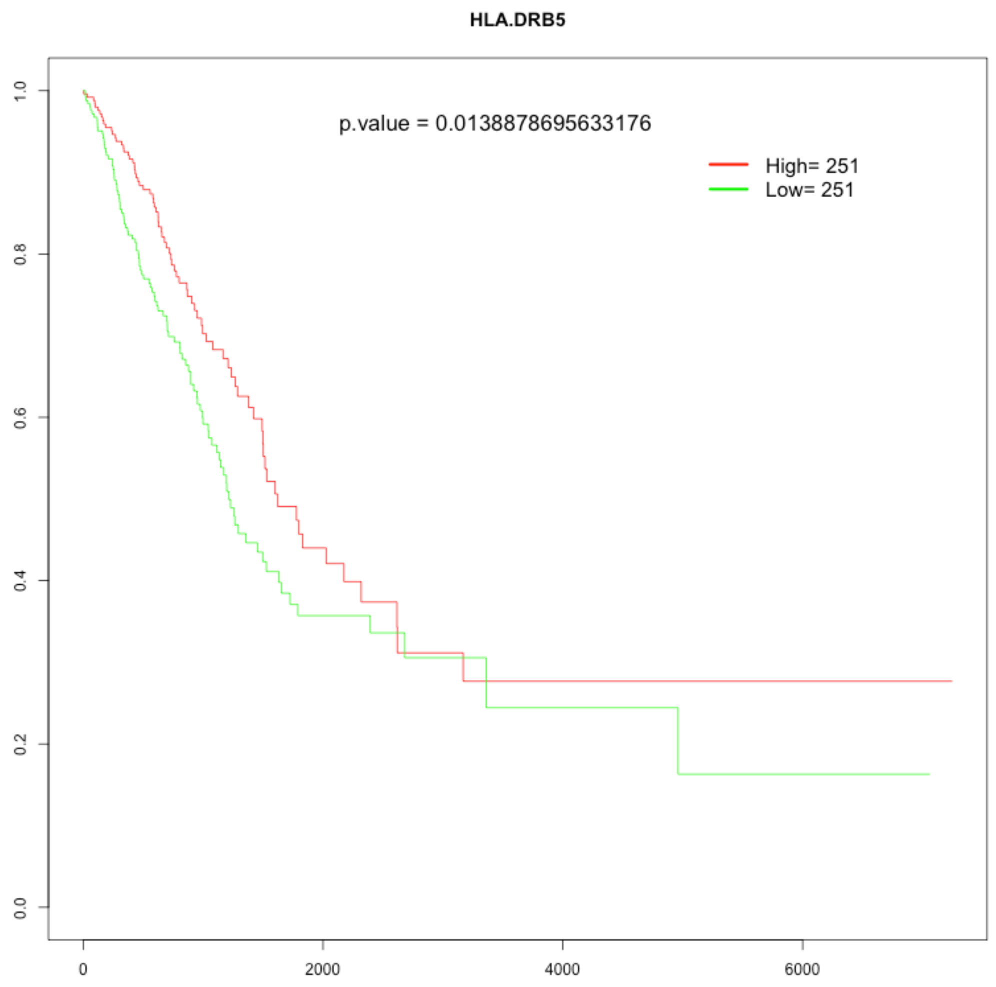

HLA.DRB5
p.value = 0.0138878695633176
High= 251
Low= 251
0.0
0.2
0.4
0.6
0.8
1.0
0
2000
4000
6000

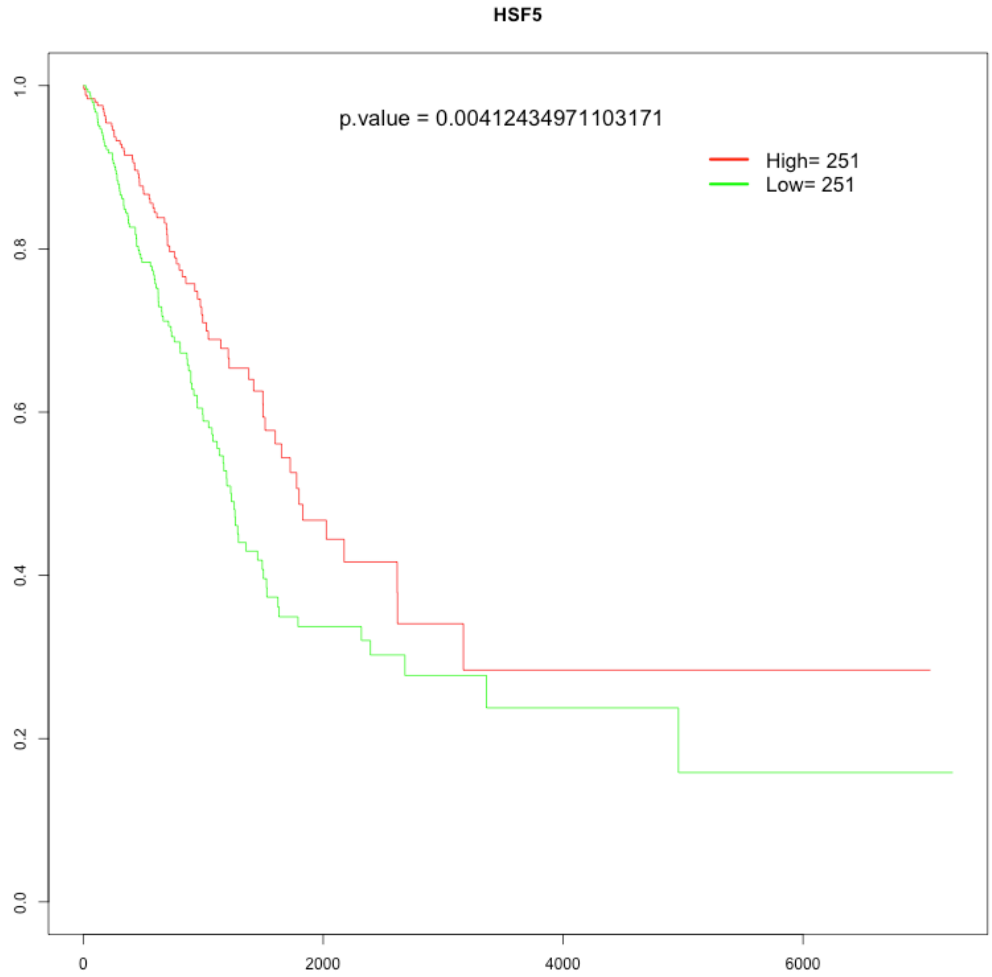

HSF5
p.value = 0.00412434971103171
High= 251
Low= 251
1.0
0.8
0.6
0.4
0.2
0.0
0
2000
4000
6000

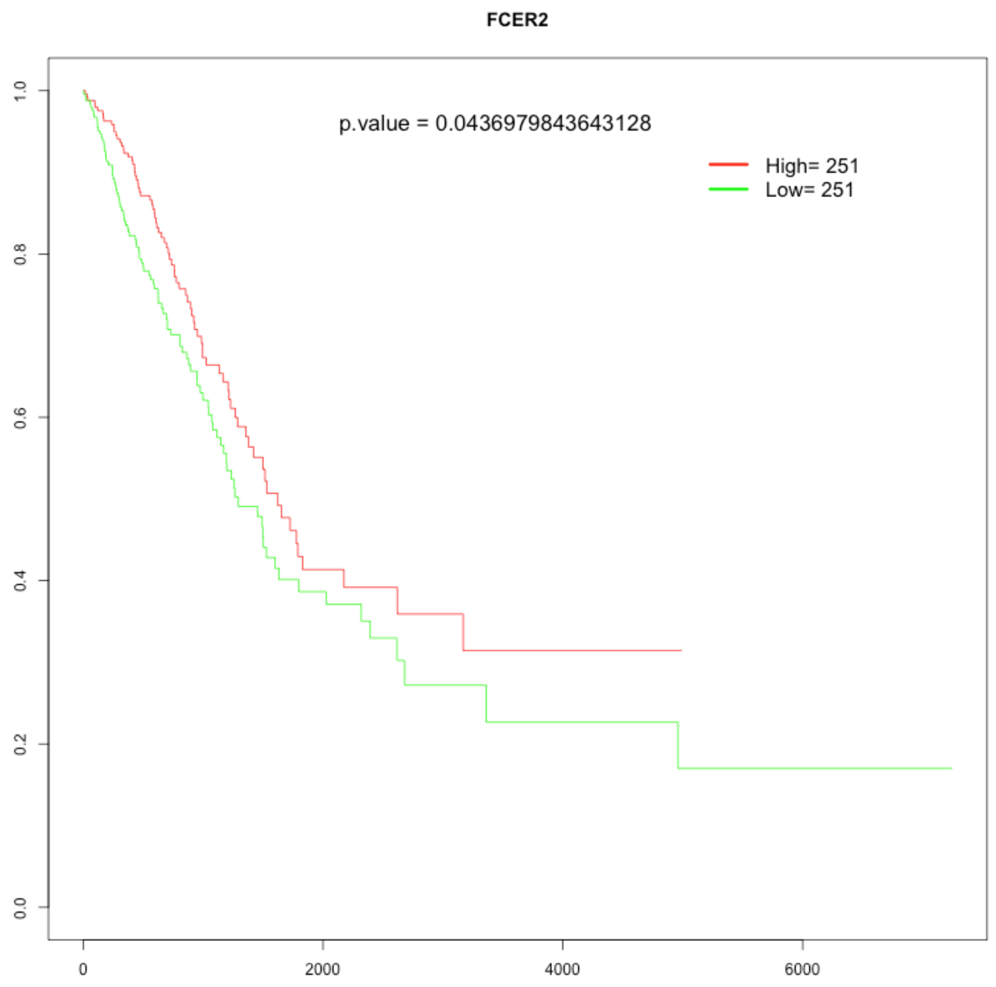

FCER2
p.value = 0.0436979843643128
High= 251
Low= 251
1.0
0.8
0.6
0.4
0.2
0.0
0
2000
4000
6000

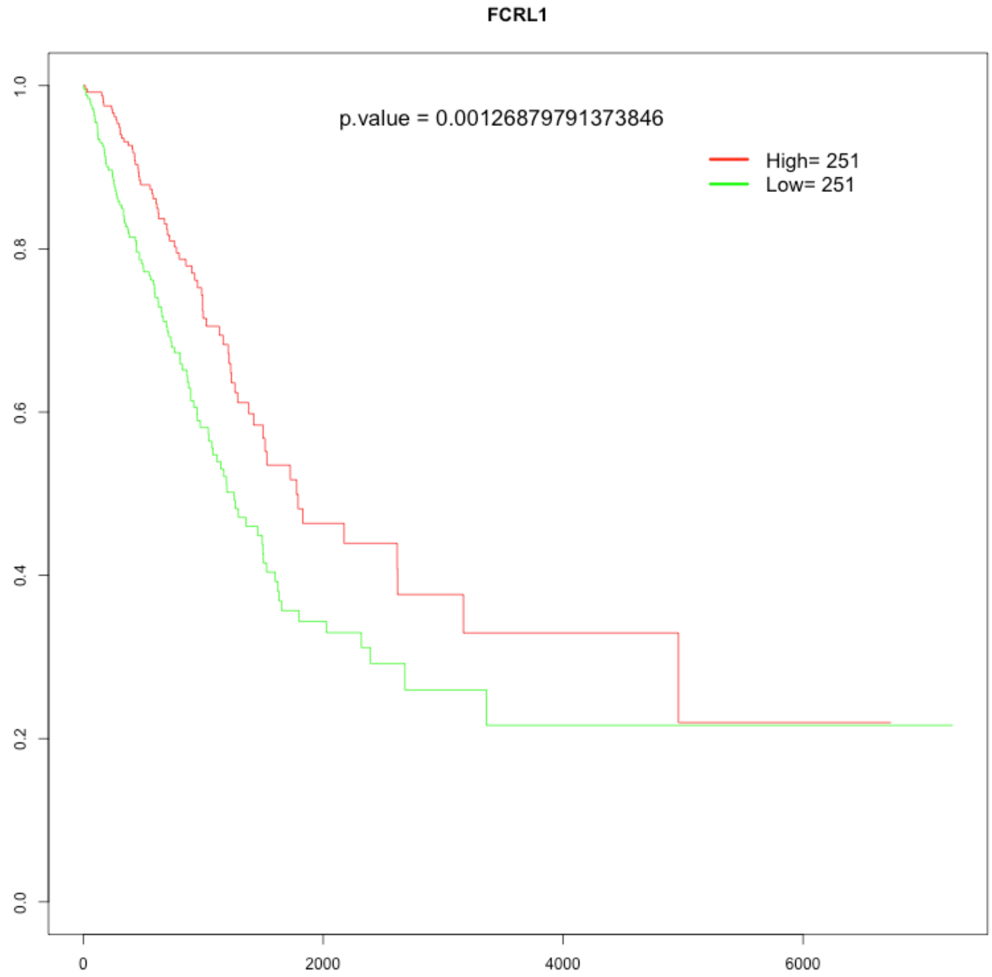

FCRL1
p.value = 0.00126879791373846
High= 251
Low= 251
1.0
0.8
0.6
0.4
0.2
0.0
0
2000
4000
6000

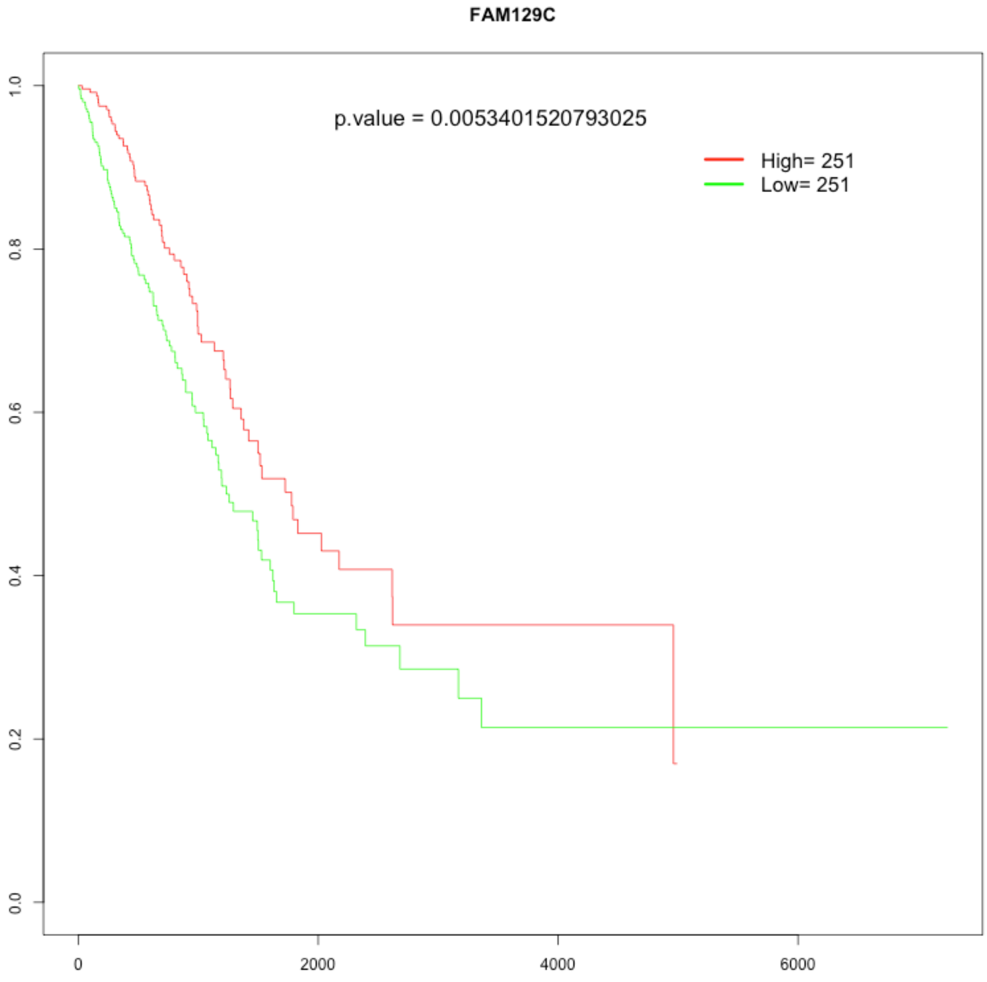

FAM129C
p.value = 0.0053401520793025
High= 251
Low= 251
1.0
0.8
0.6
0.4
0.2
0.0
0
2000
4000
6000

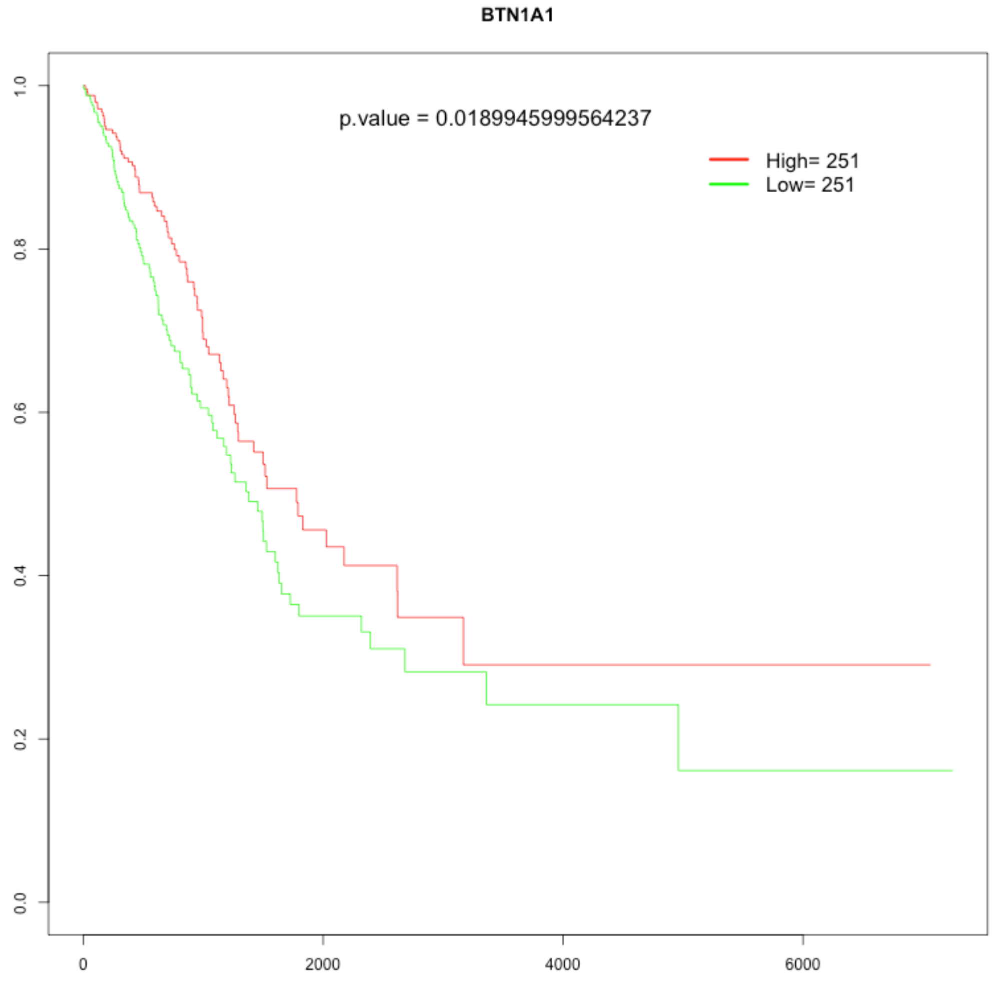
BTN1A1
p.value = 0.0189945999564237
High= 251
Low= 251
1.0
0.8
0.6
0.4
0.2
0.0
0
2000
4000
6000

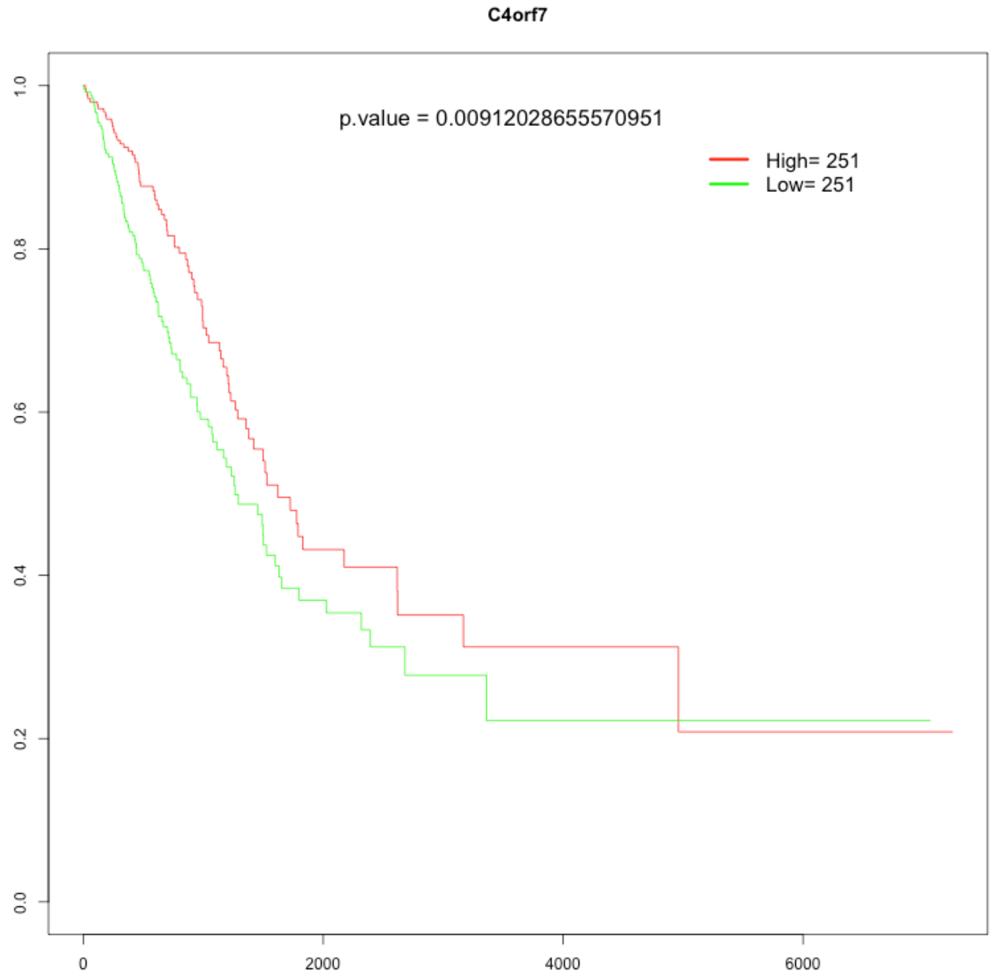
C4orf7
p.value = 0.00912028655570951
High= 251
Low= 251
1.0
0.8
0.6
0.4
0.2
0.0
0
2000
4000
6000

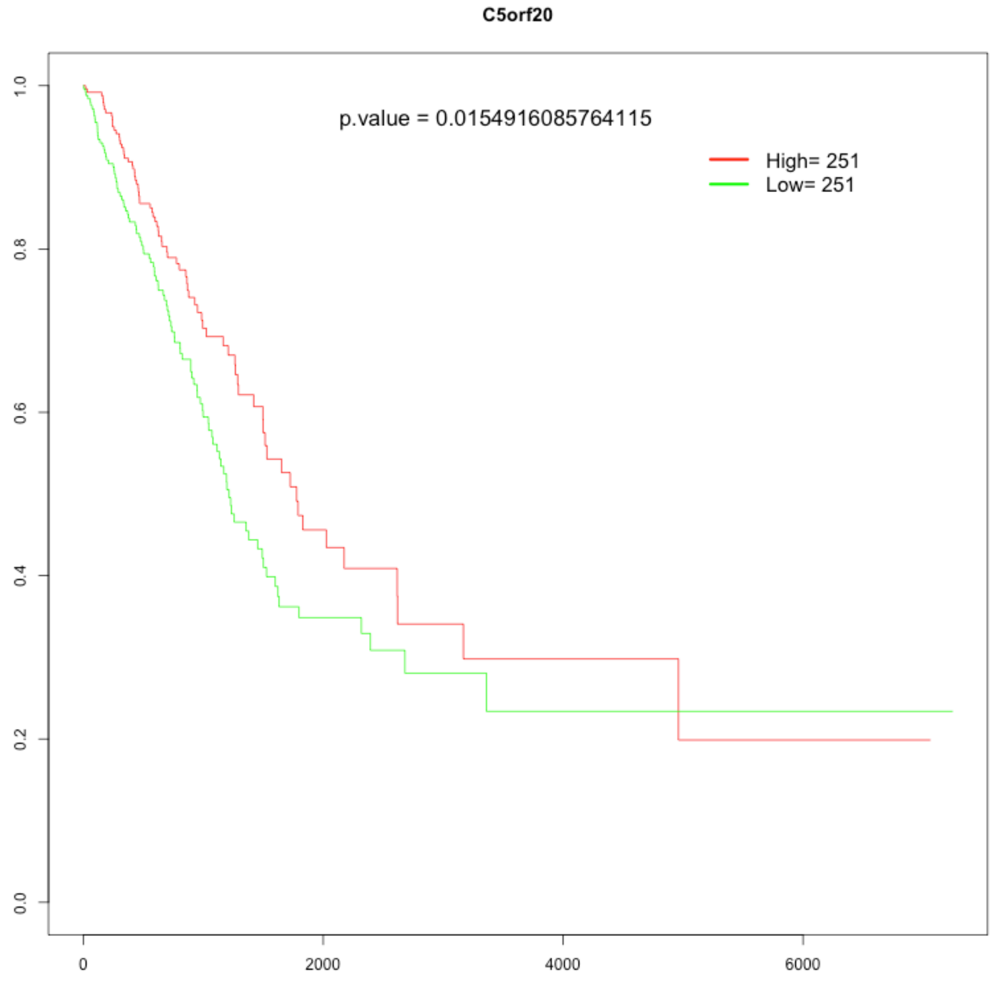

C5orf20
p.value = 0.0154916085764115
High= 251
Low= 251
0.0
0.2
0.4
0.6
0.8
1.0
0
2000
4000
6000

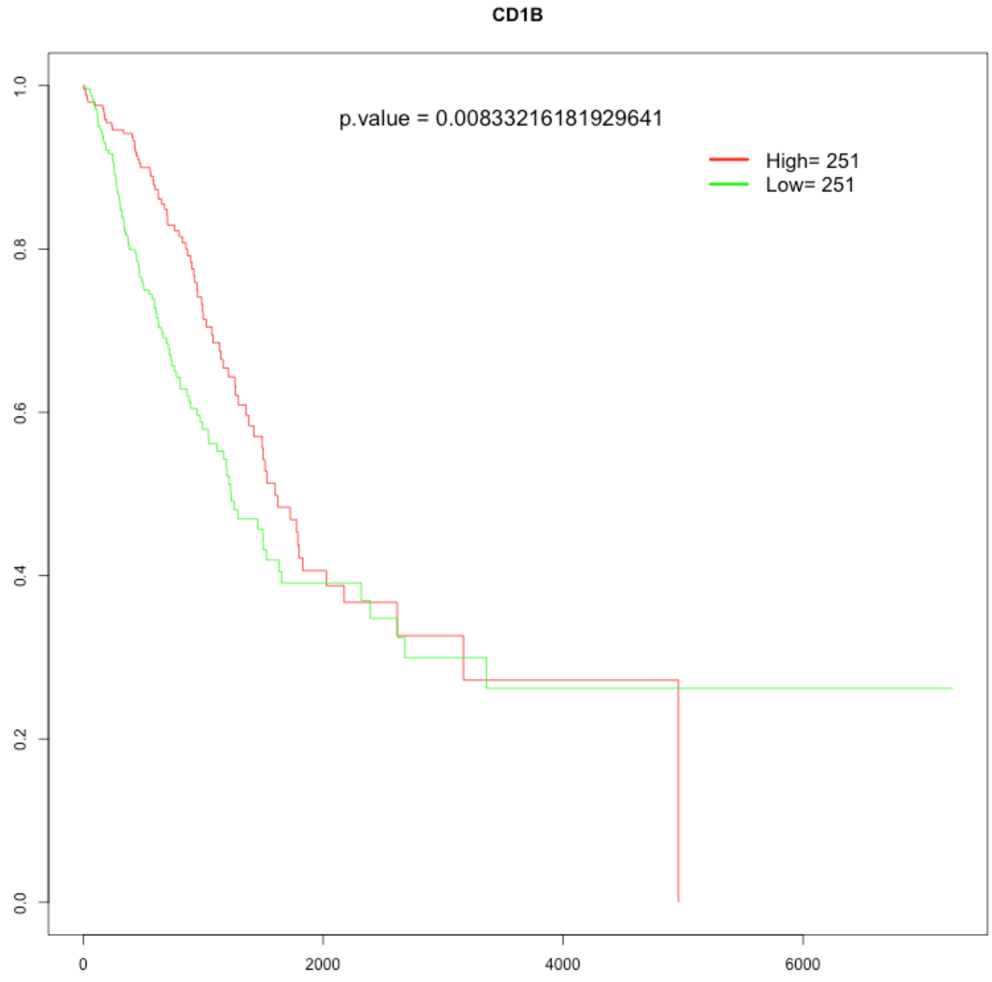
CD1B
p.value = 0.00833216181929641
High= 251
Low= 251
1.0
0.8
0.6
0.4
0.2
0.0
0
2000
4000
6000

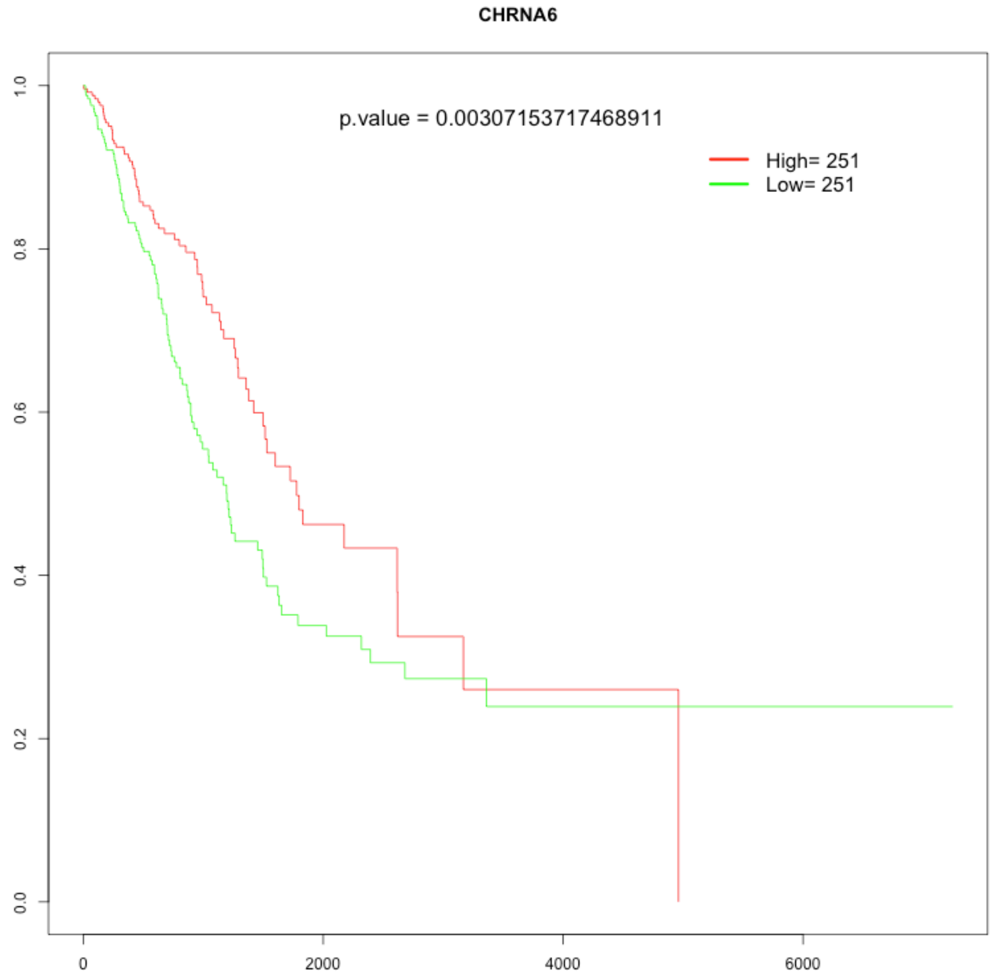

CHRNA6
p.value = 0.00307153717468911
High= 251
Low= 251
1.0
0.8
0.6
0.4
0.2
0.0
0
2000
4000
6000

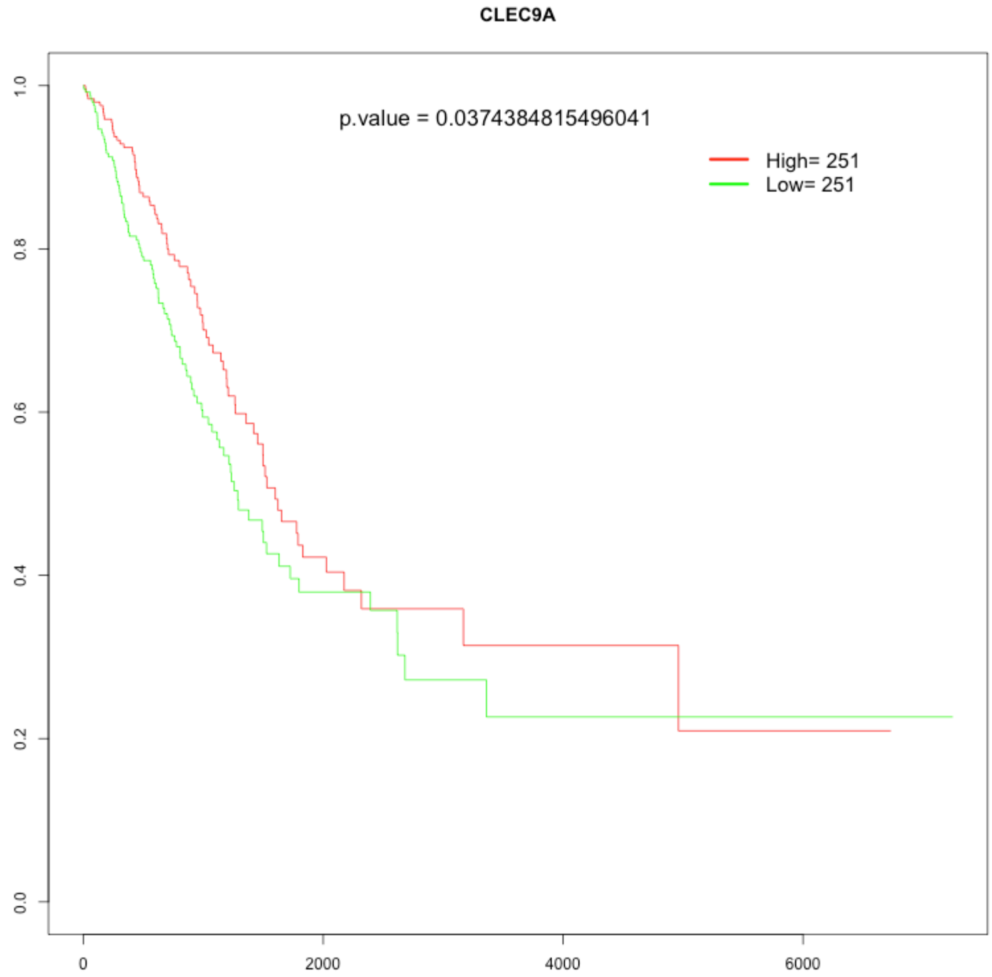
CLEC9A
p.value = 0.0374384815496041
High= 251
Low= 251
1.0
0.8
0.6
0.4
0.2
0.0
0
2000
4000
6000

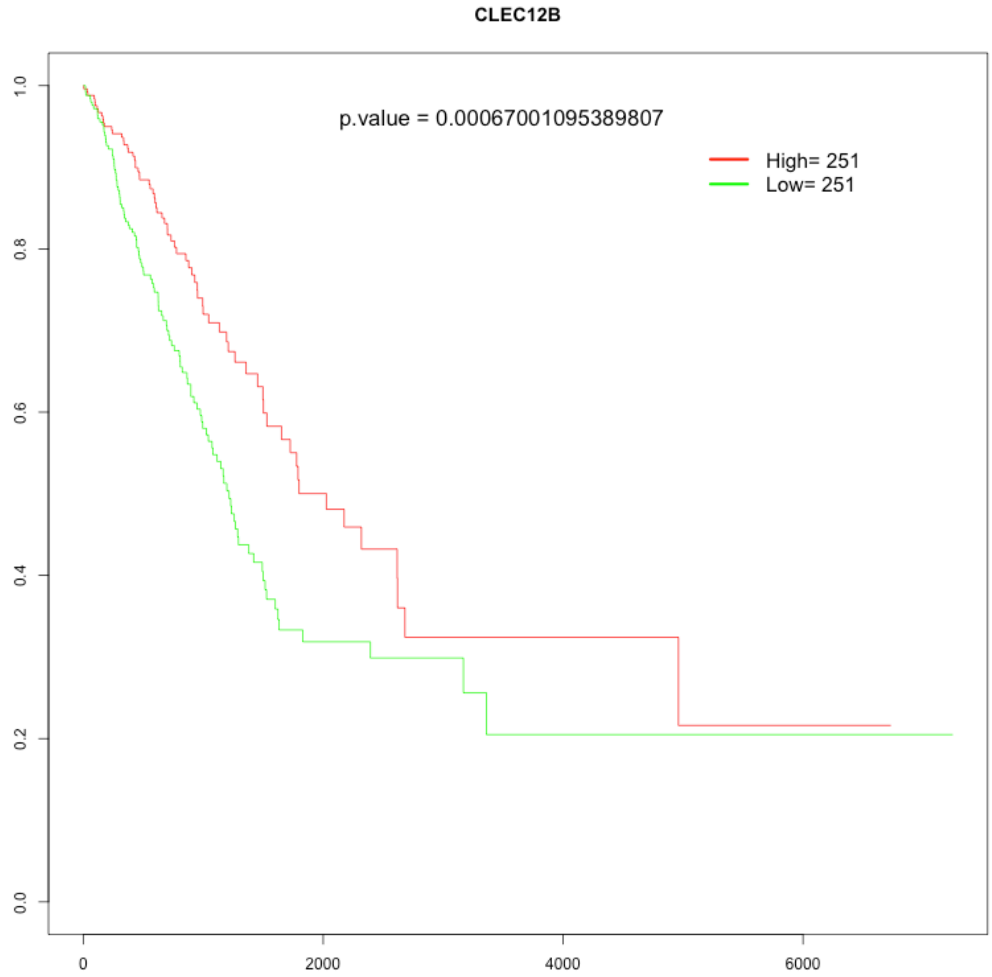
CLEC12B
p.value = 0.00067001095389807
High= 251
Low= 251
0.0
0.2
0.4
0.6
0.8
1.0
0
2000
4000
6000

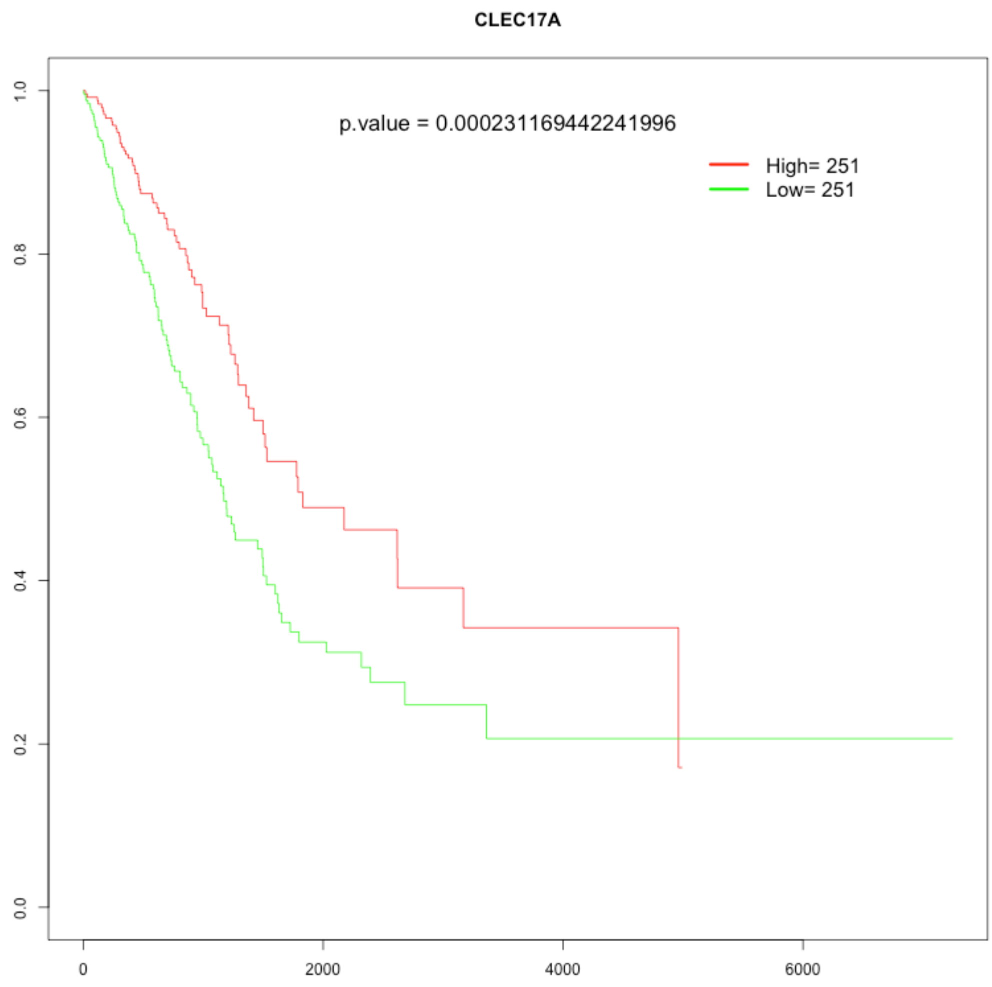
CLEC17A
p.value = 0.000231169442241996
High= 251
Low= 251
1.0
0.8
0.6
0.4
0.2
0.0
0
2000
4000
6000

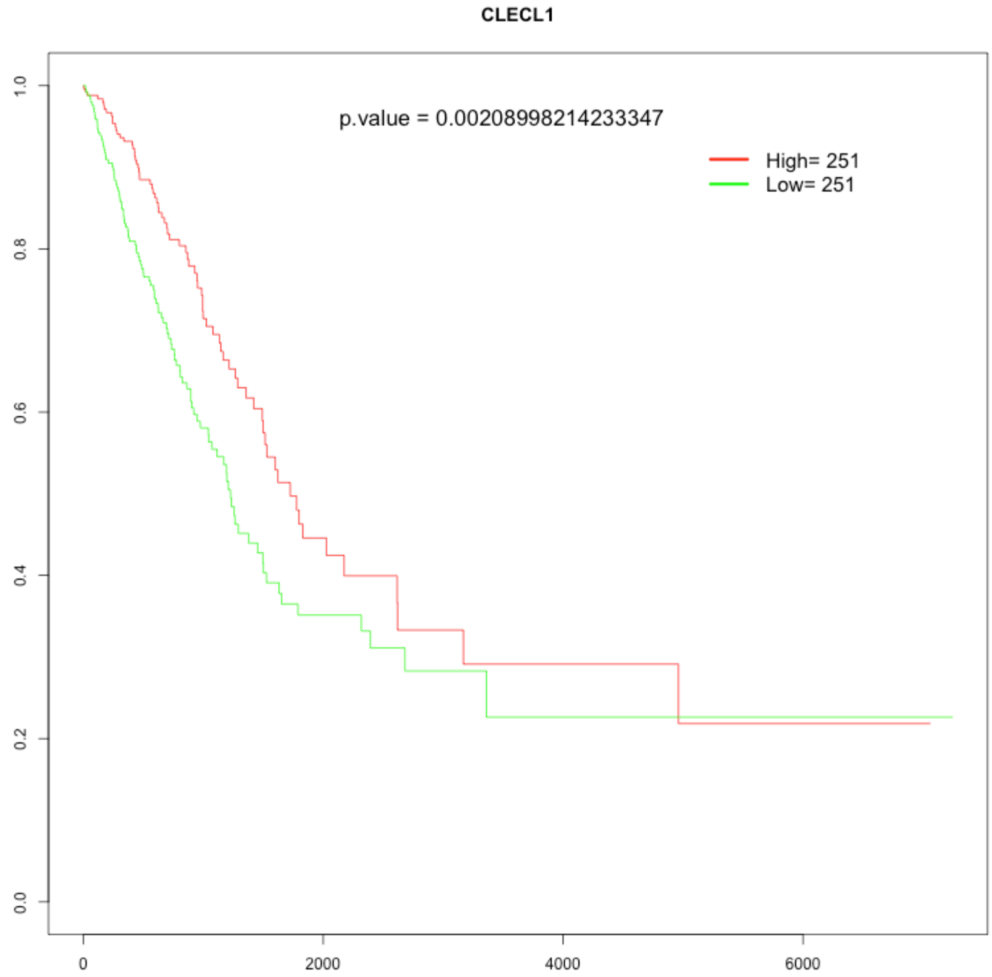

CLECL1
p.value = 0.00208998214233347
High= 251
Low= 251
1.0
0.8
0.6
0.4
0.2
0.0
0
2000
4000
6000

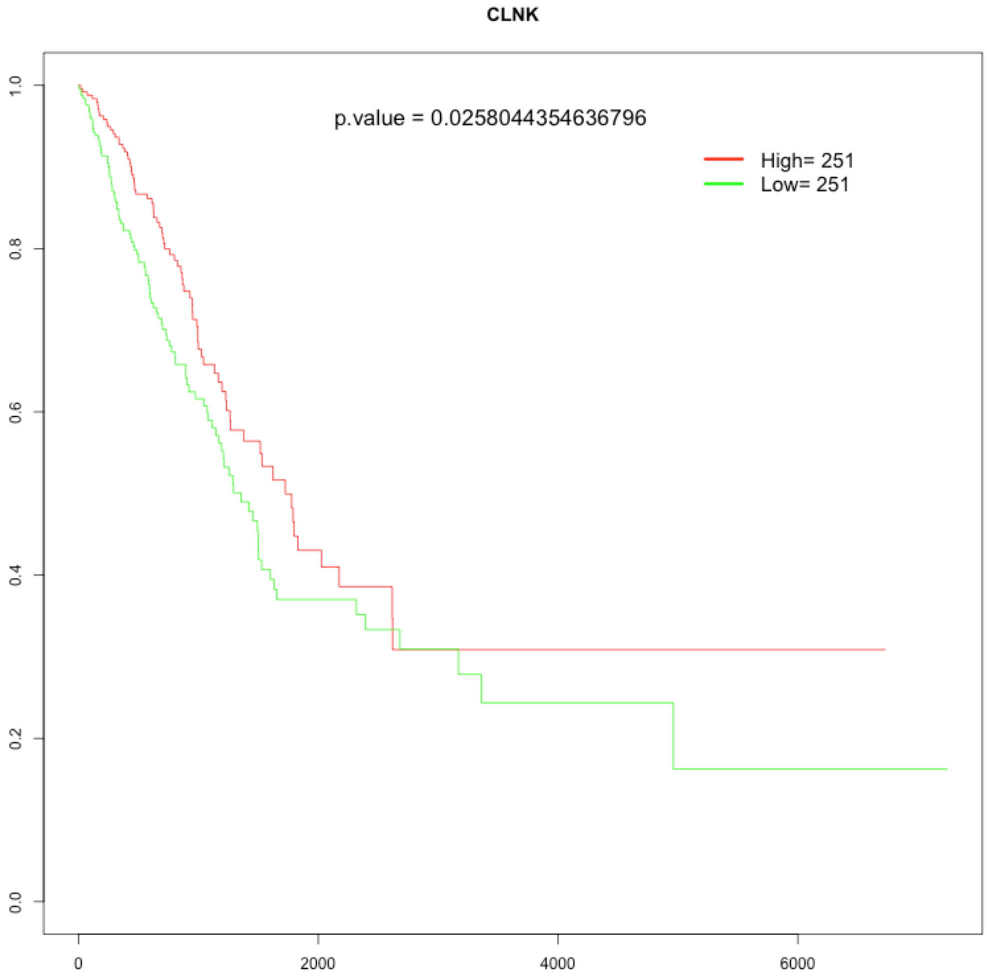
CLNK
p.value = 0.0258044354636796
High= 251
Low= 251
0.0
0.2
0.4
0.6
0.8
1.0
0
2000
4000
6000

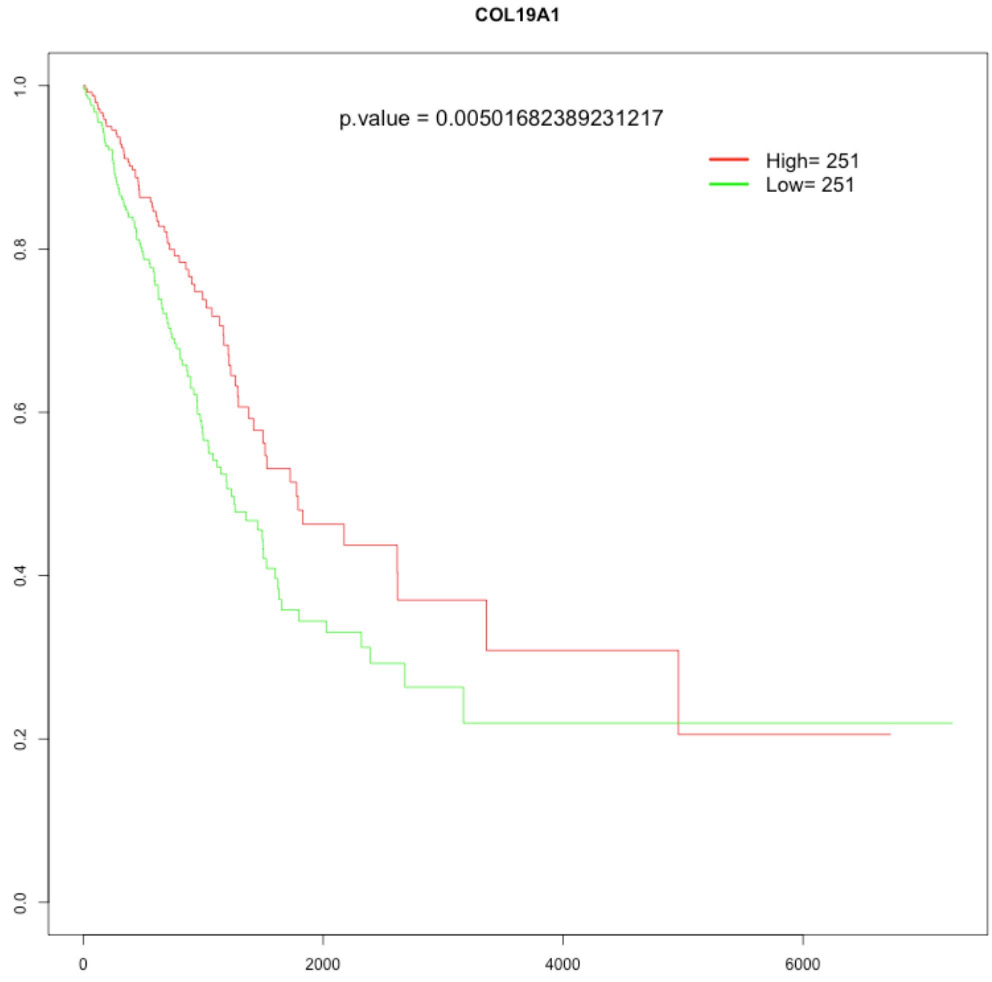
COL19A1
p.value = 0.00501682389231217
High= 251
Low= 251
0.0
0.2
0.4
0.6
0.8
1.0
0
2000
4000
6000

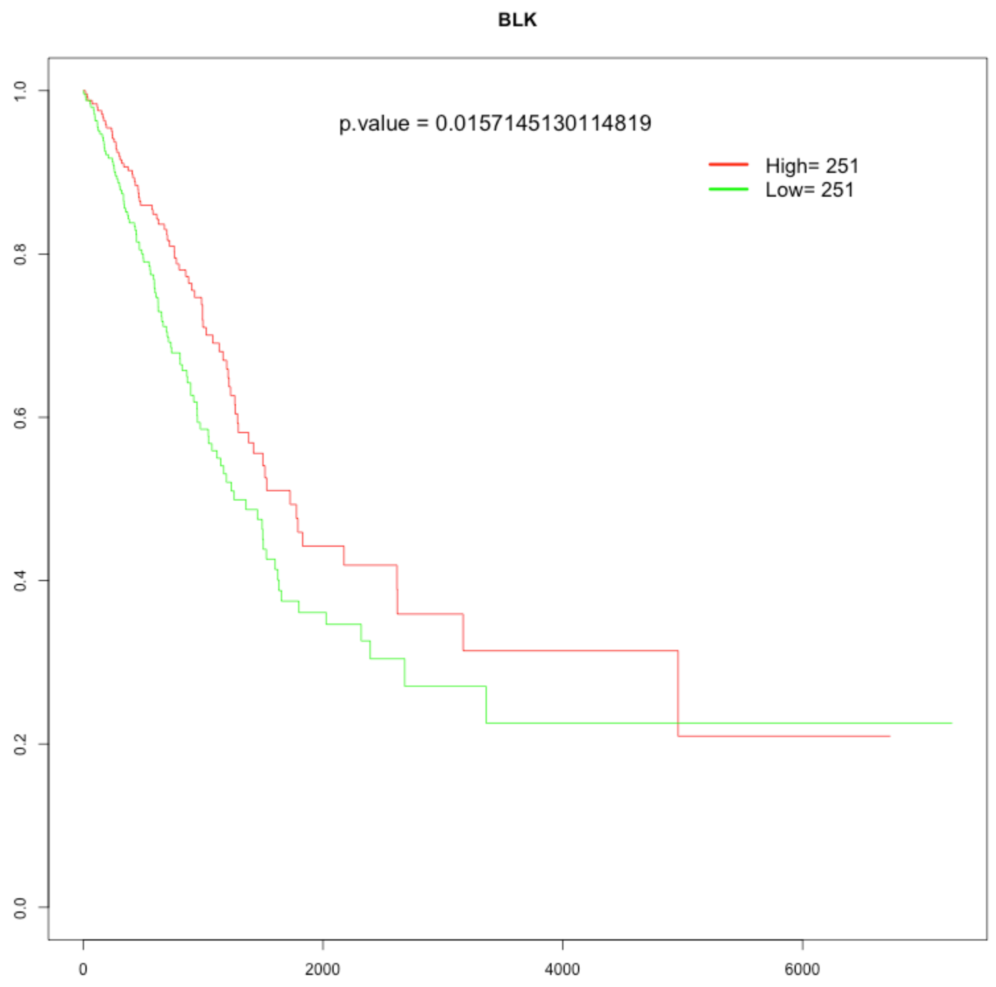

BLK
p.value = 0.0157145130114819
High= 251
Low= 251
0.0
0.2
0.4
0.6
0.8
1.0
0
2000
4000
6000
