# Supplementary material for: Identification of Immune-Related Gene Signatures in Lung Adenocarcinoma and Lung Squamous Cell Carcinoma
Source: Front Immunol. 2021 Nov 23;12:752643. doi: 10.3389/fimmu.2021.752643 (PMC8649721; doi:10.3389/fimmu.2021.752643)
Supplement: Supplementary file 3 [file DataSheet_3.zip › Supplementary figure 6_v1.pdf]

Supplementary figure 6

Kaplan–Meier survival analysis for differentially expressed genes in LUSC.

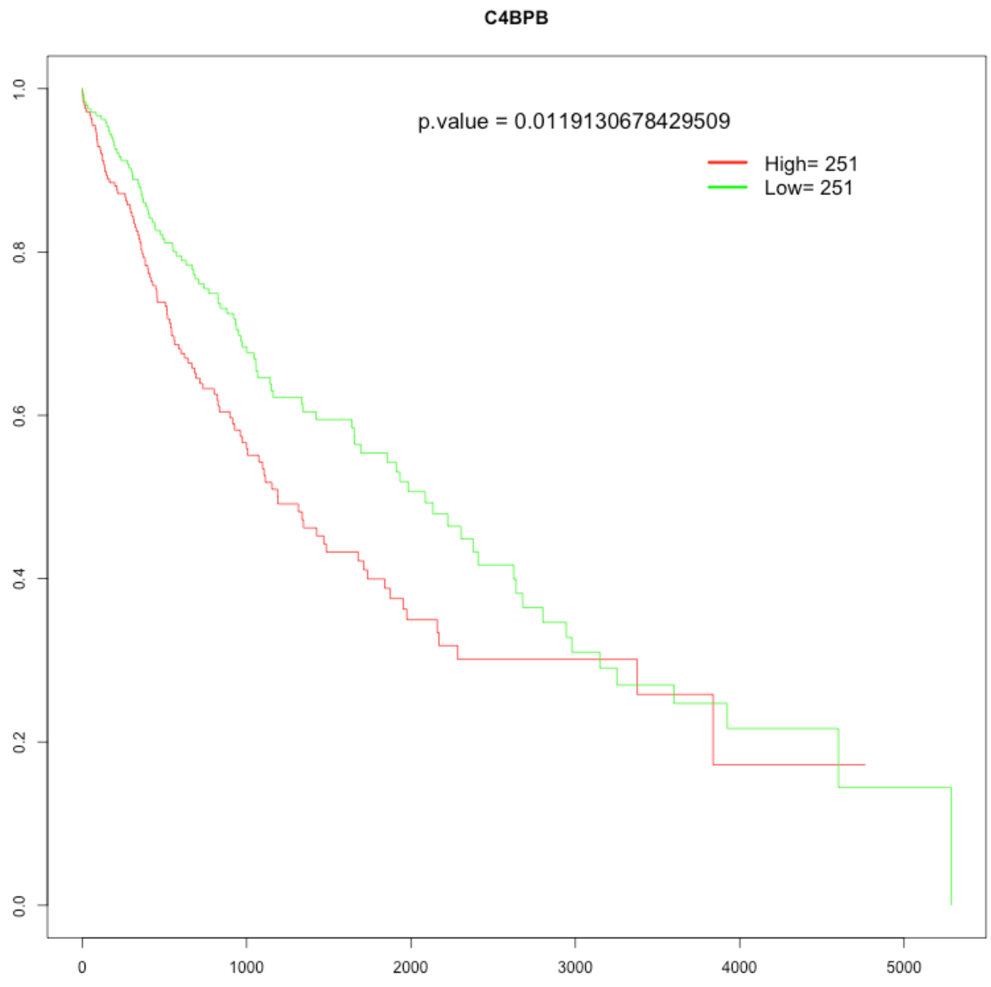

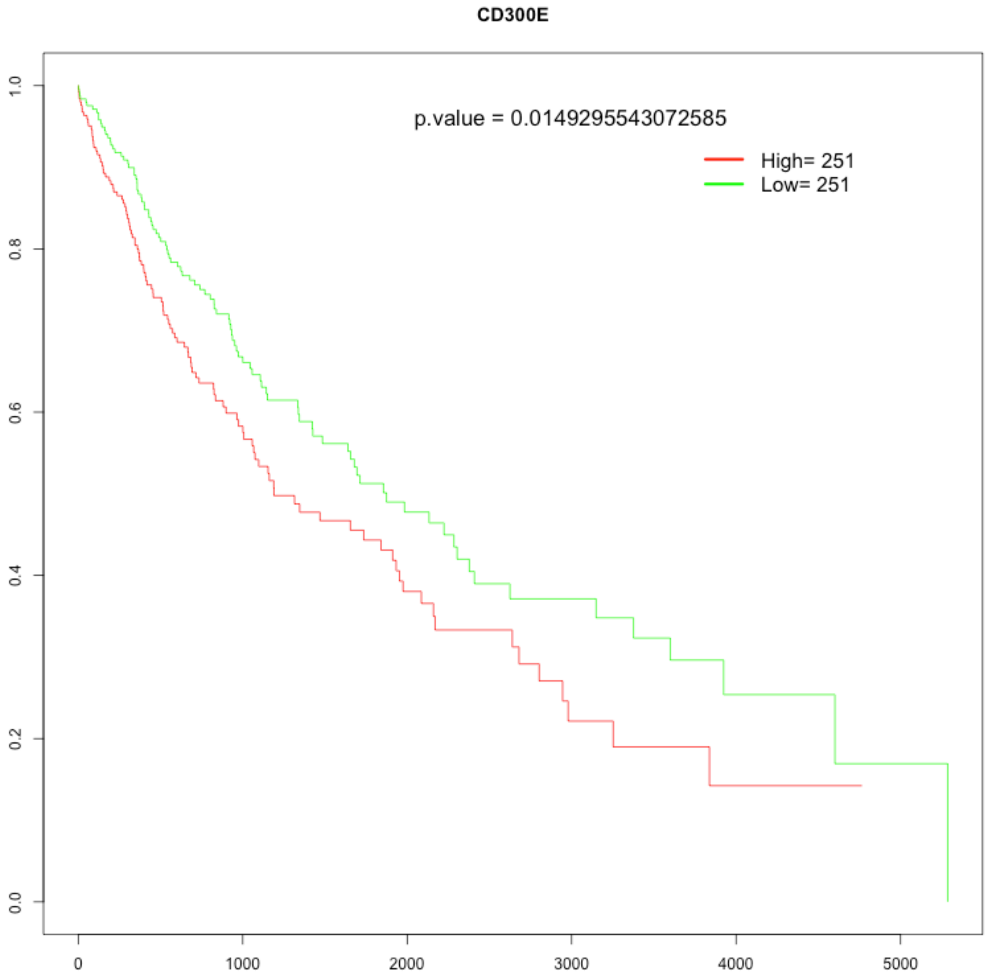

CD300E
p.value = 0.0149295543072585
High= 251
Low= 251
1.0
0.8
0.6
0.4
0.2
0.0
0
1000
2000
3000
4000
5000

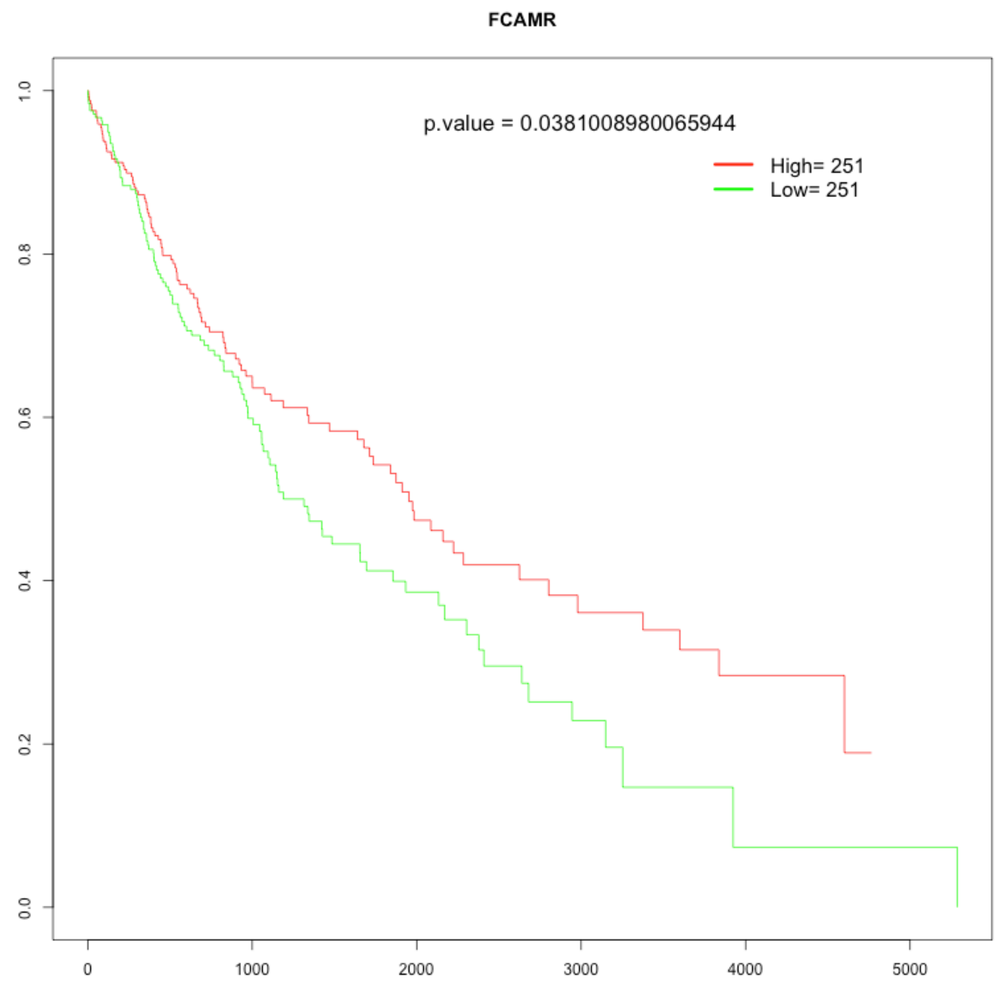
FCAMR
p.value = 0.0381008980065944
High= 251
Low= 251
0.0
0.2
0.4
0.6
0.8
1.0
0
1000
2000
3000
4000
5000

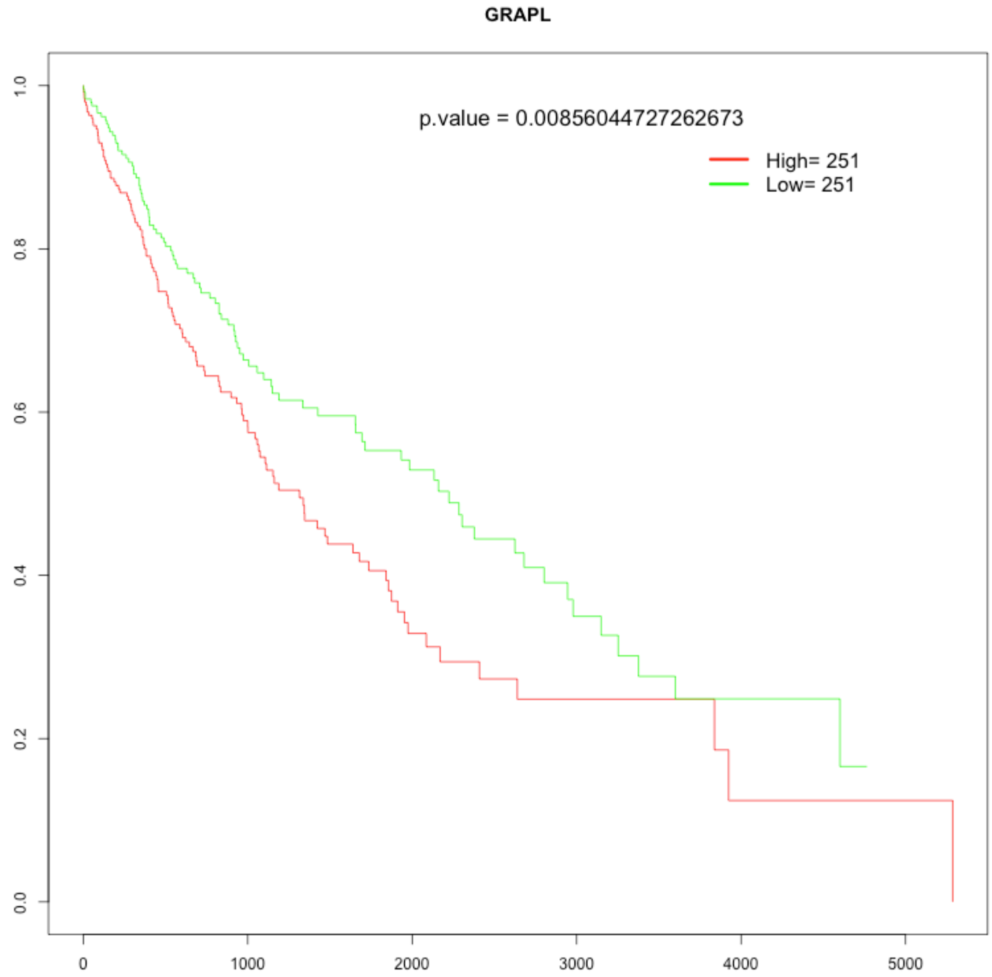
GRAPL
p.value = 0.00856044727262673
High= 251
Low= 251
0.0
0.2
0.4
0.6
0.8
1.0
0
1000
2000
3000
4000
5000

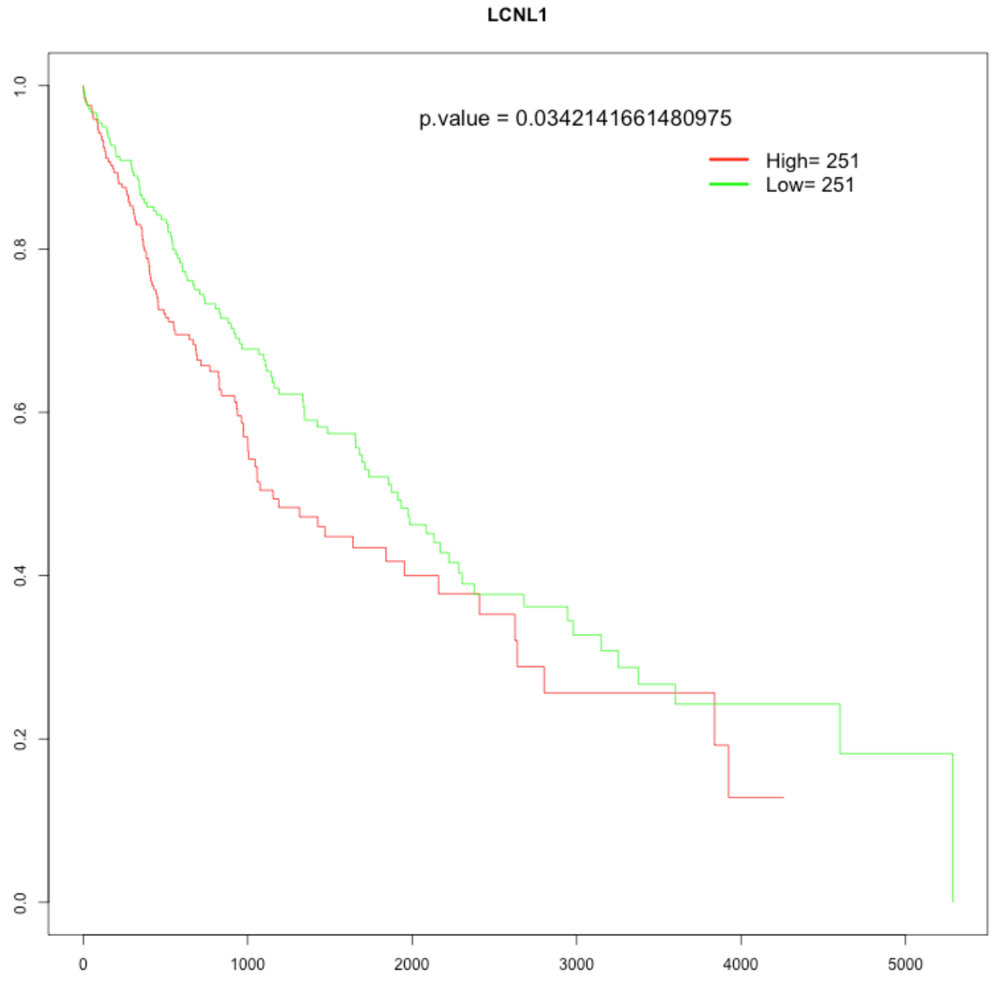
LCNL1
p.value = 0.0342141661480975
High= 251
Low= 251
1.0
0.8
0.6
0.4
0.2
0.0
0
1000
2000
3000
4000
5000

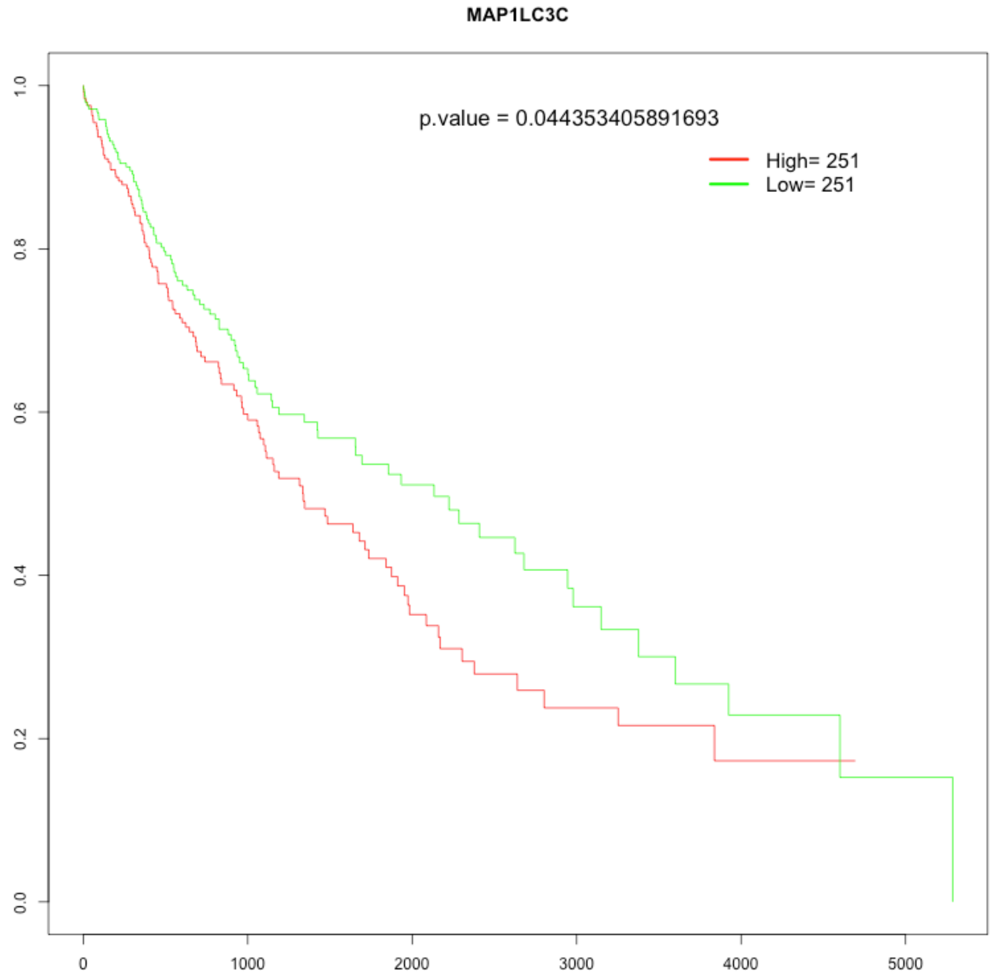
MAP1LC3C
p.value = 0.044353405891693
High= 251
Low= 251
1.0
0.8
0.6
0.4
0.2
0.0
0
1000
2000
3000
4000
5000

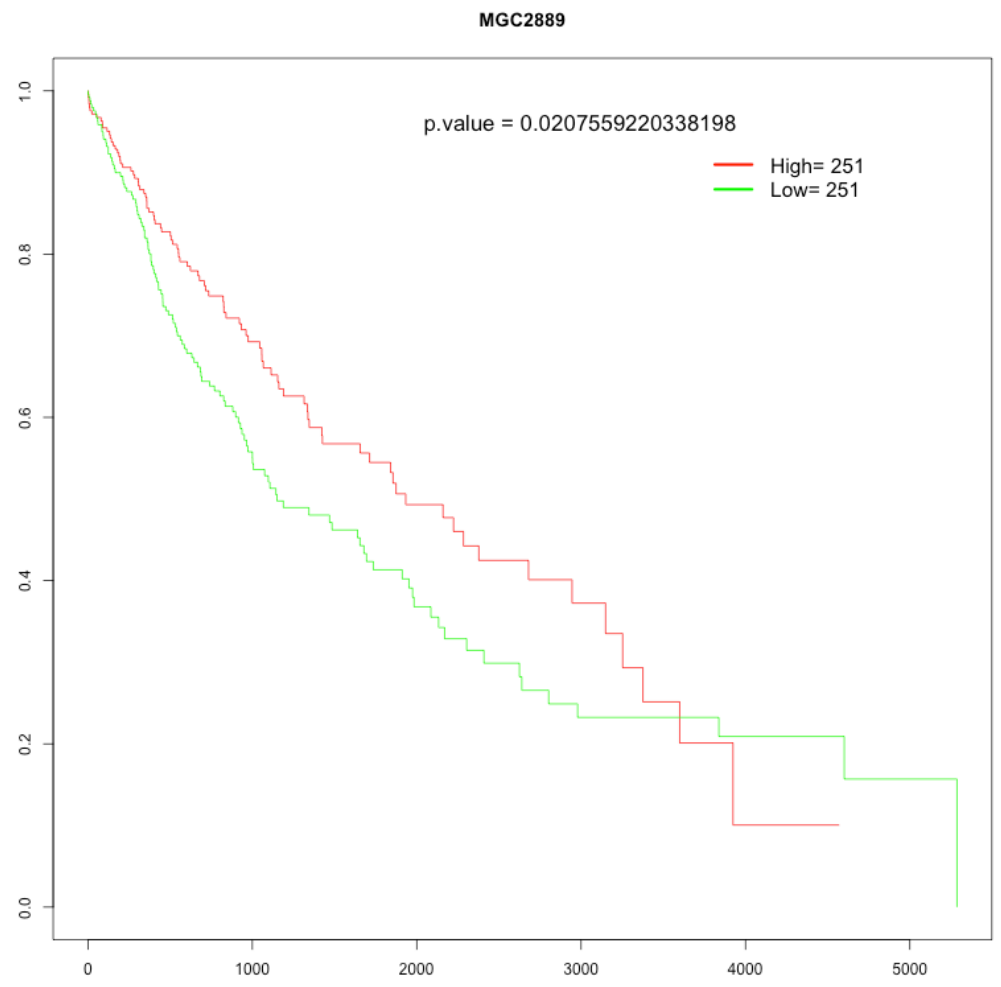
MGC2889
p.value = 0.0207559220338198
High= 251
Low= 251
1.0
0.8
0.6
0.4
0.2
0.0
0
1000
2000
3000
4000
5000

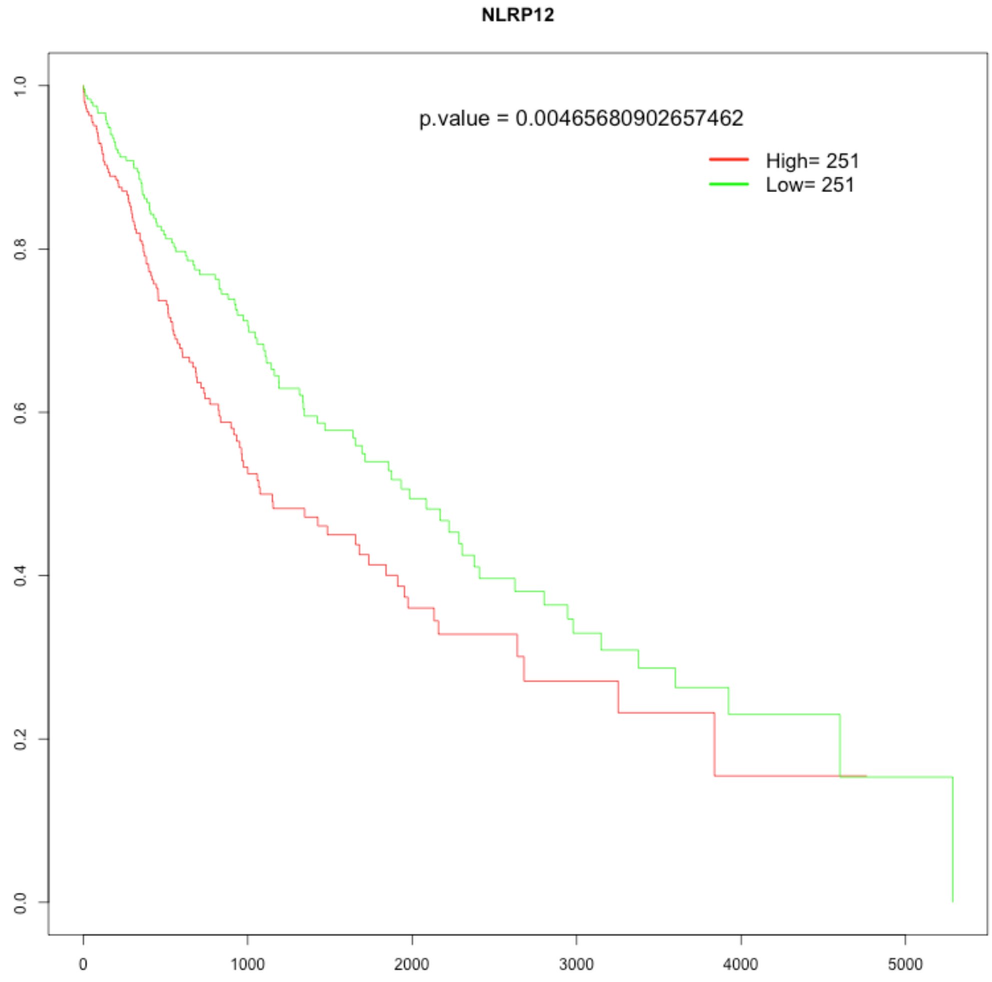
NLRP12
p.value = 0.00465680902657462
High= 251
Low= 251
1.0
0.8
0.6
0.4
0.2
0.0
0
1000
2000
3000
4000
5000

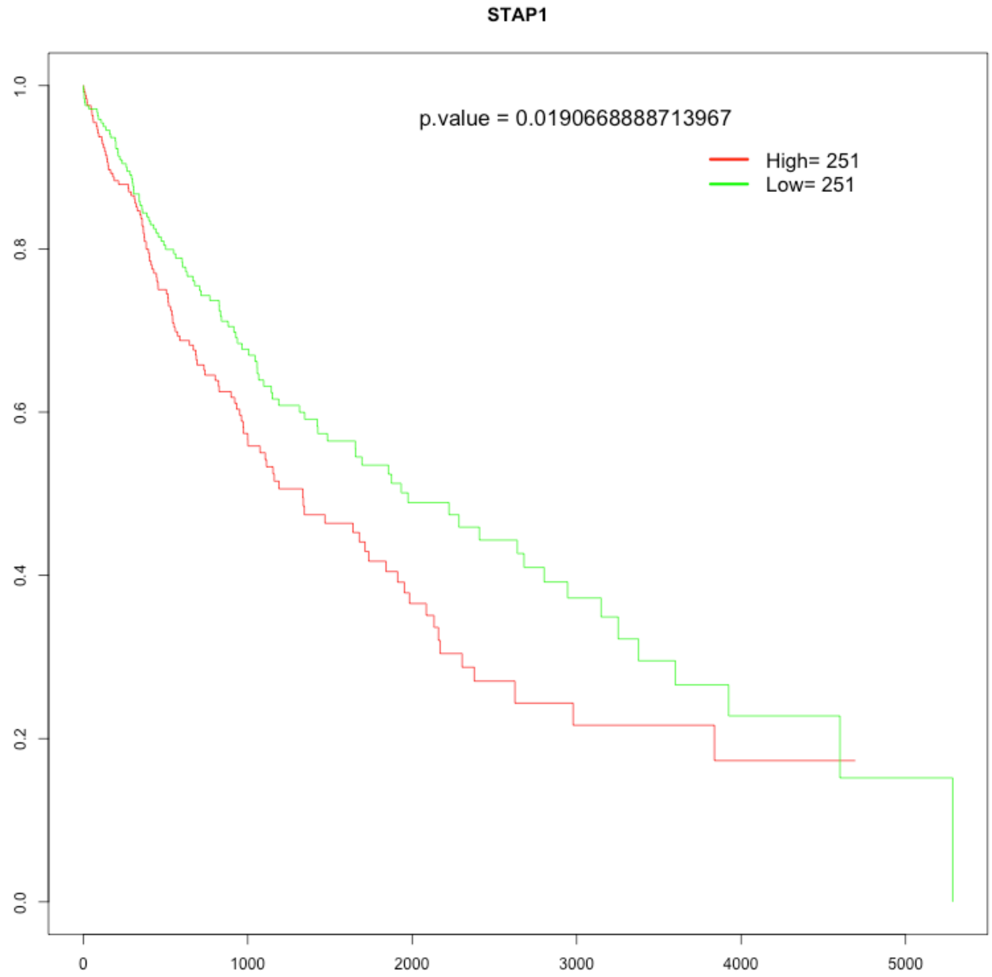
STAP1
p.value = 0.0190668888713967
High= 251
Low= 251
0.0
0.2
0.4
0.6
0.8
1.0
0
1000
2000
3000
4000
5000

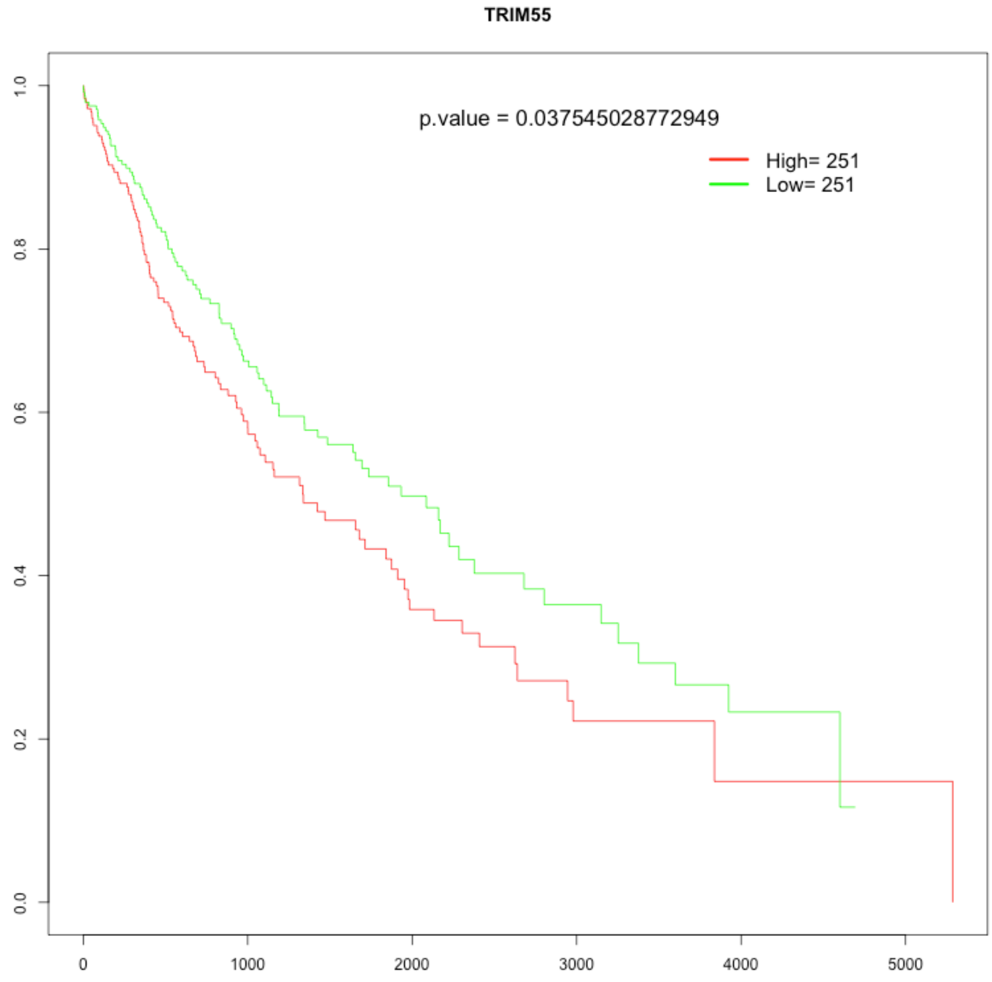
TRIM55
p.value = 0.037545028772949
High= 251
Low= 251
0.0
0.2
0.4
0.6
0.8
1.0
0
1000
2000
3000
4000
5000

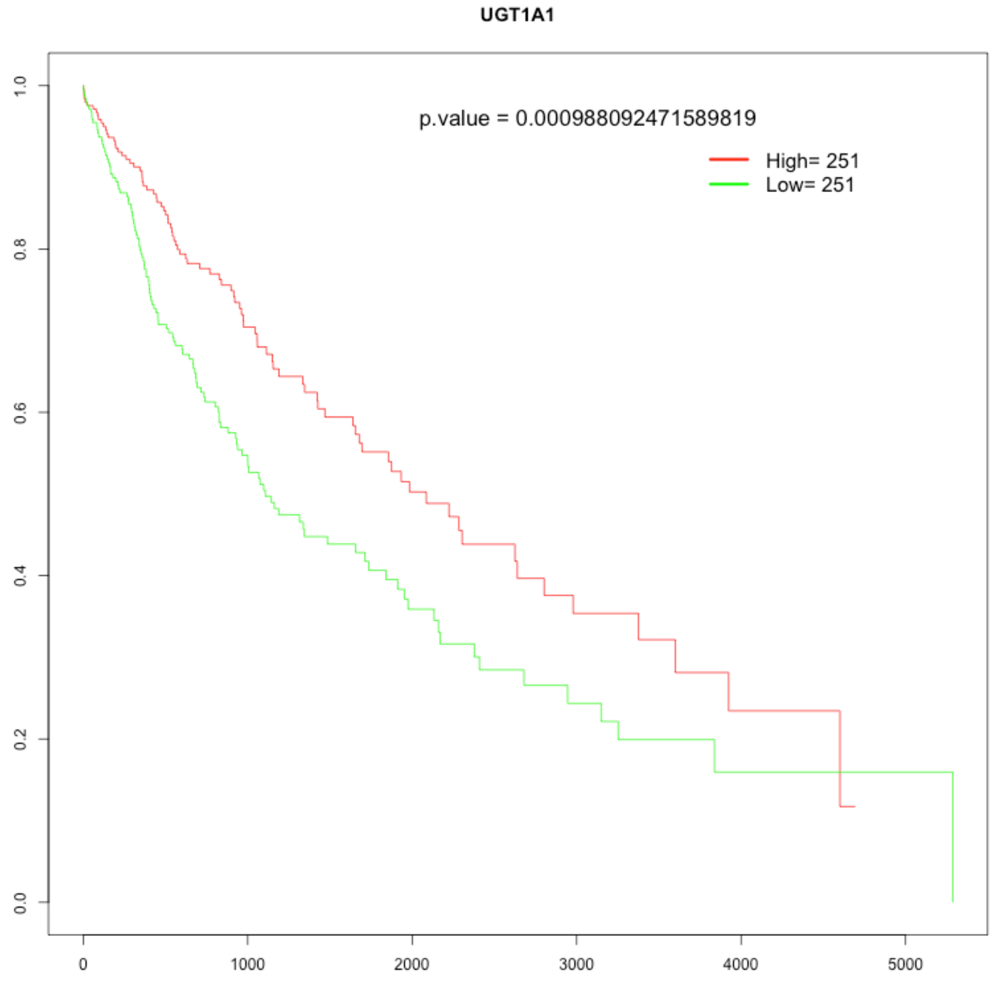
UGT1A1
p.value = 0.000988092471589819
High= 251
Low= 251
0.0
0.2
0.4
0.6
0.8
1.0
0
1000
2000
3000
4000
5000

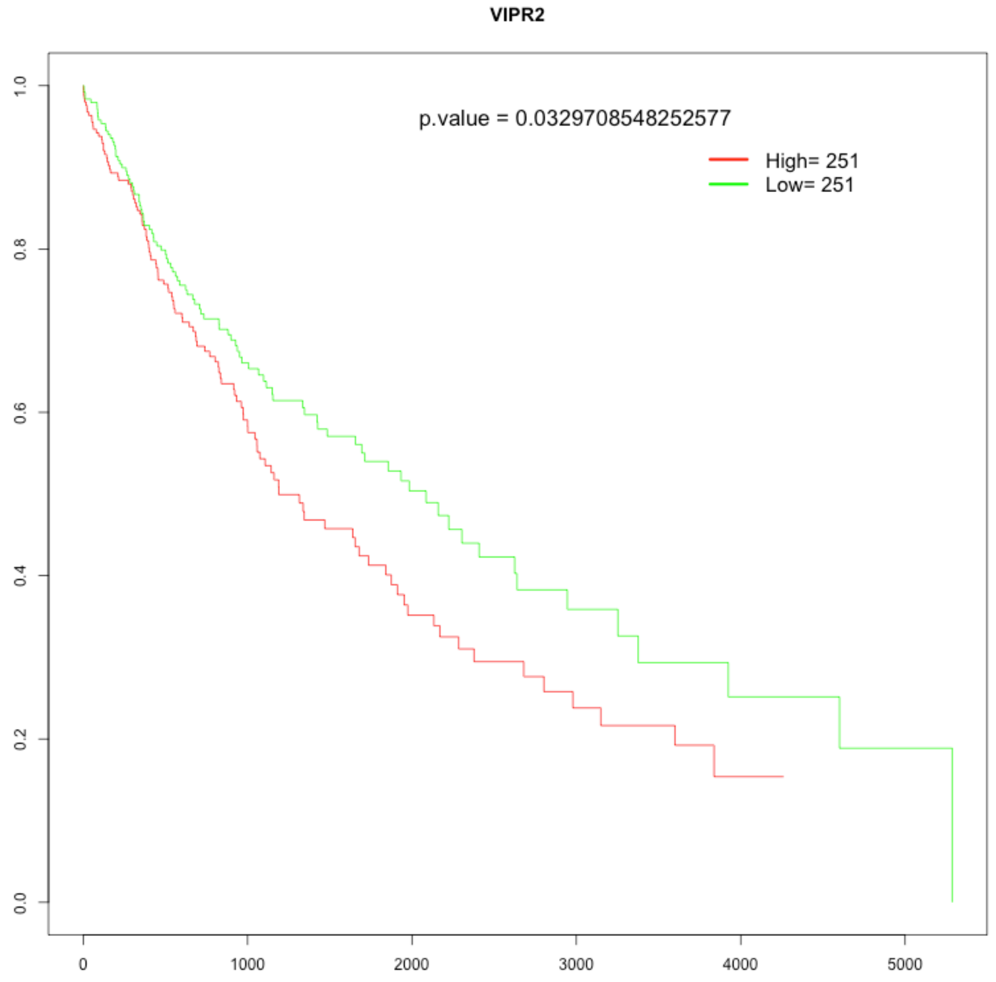

VIPR2
p.value = 0.0329708548252577
High= 251
Low= 251
1.0
0.8
0.6
0.4
0.2
0.0
0
1000
2000
3000
4000
5000
