# Supplementary material for: Identification of Immune-Related Gene Signatures in Lung Adenocarcinoma and Lung Squamous Cell Carcinoma
Source: Front Immunol. 2021 Nov 23;12:752643. doi: 10.3389/fimmu.2021.752643 (PMC8649721; doi:10.3389/fimmu.2021.752643)
Supplement: Supplementary file 3 [file DataSheet_3.zip › Supplementary figure 7_v1.pdf]

Supplementary figure 7

Lasso regression identified the prognostic model in LUAD and LUSC. A-B. Lasso regression complexity was controlled by lambda using the glmnet R package in LUAD. **C-D.** Lasso regression complexity was controlled by lambda using the glmnet R package in LUSC.

A.

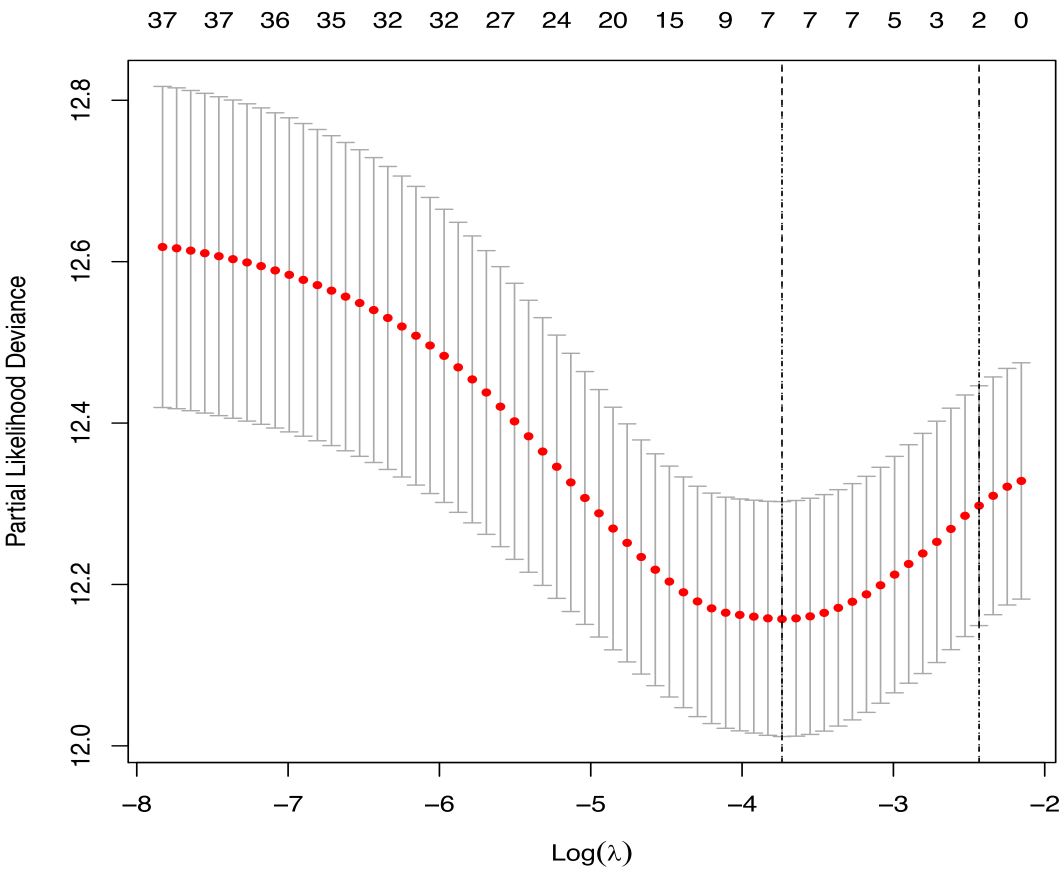

B.

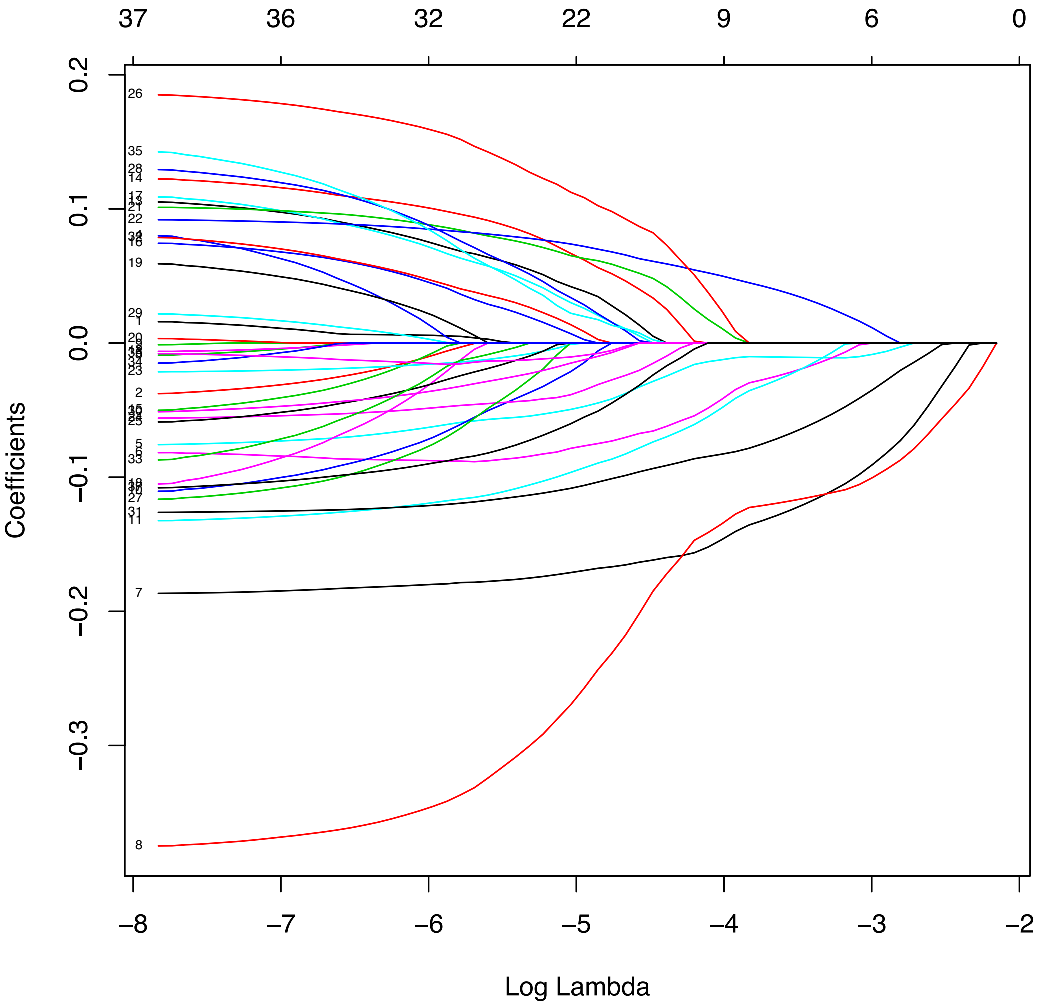
37 36 32 22 9 6 0
0.2
0.1
0.0
−0.1
−0.2
−0.3
Coefficients
−8 −7 −6 −5 −4 −3 −2
Log Lambda

C.

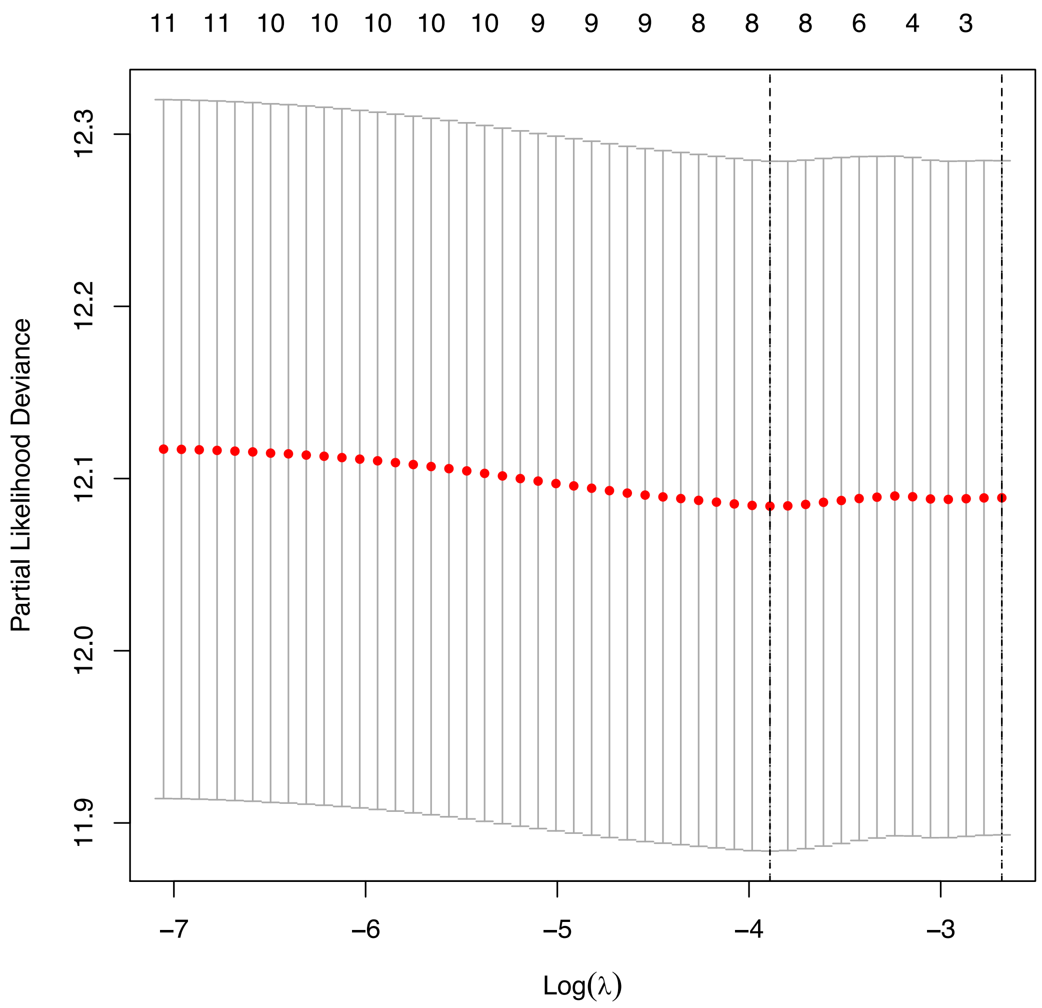
11 11 10 10 10 10 10 9 9 9 8 8 8 6 4 3
Partial Likelihood Deviance
12.3
12.2
12.1
12.0
11.9
−7
−6
−5
−4
−3
Log(λ)

D.

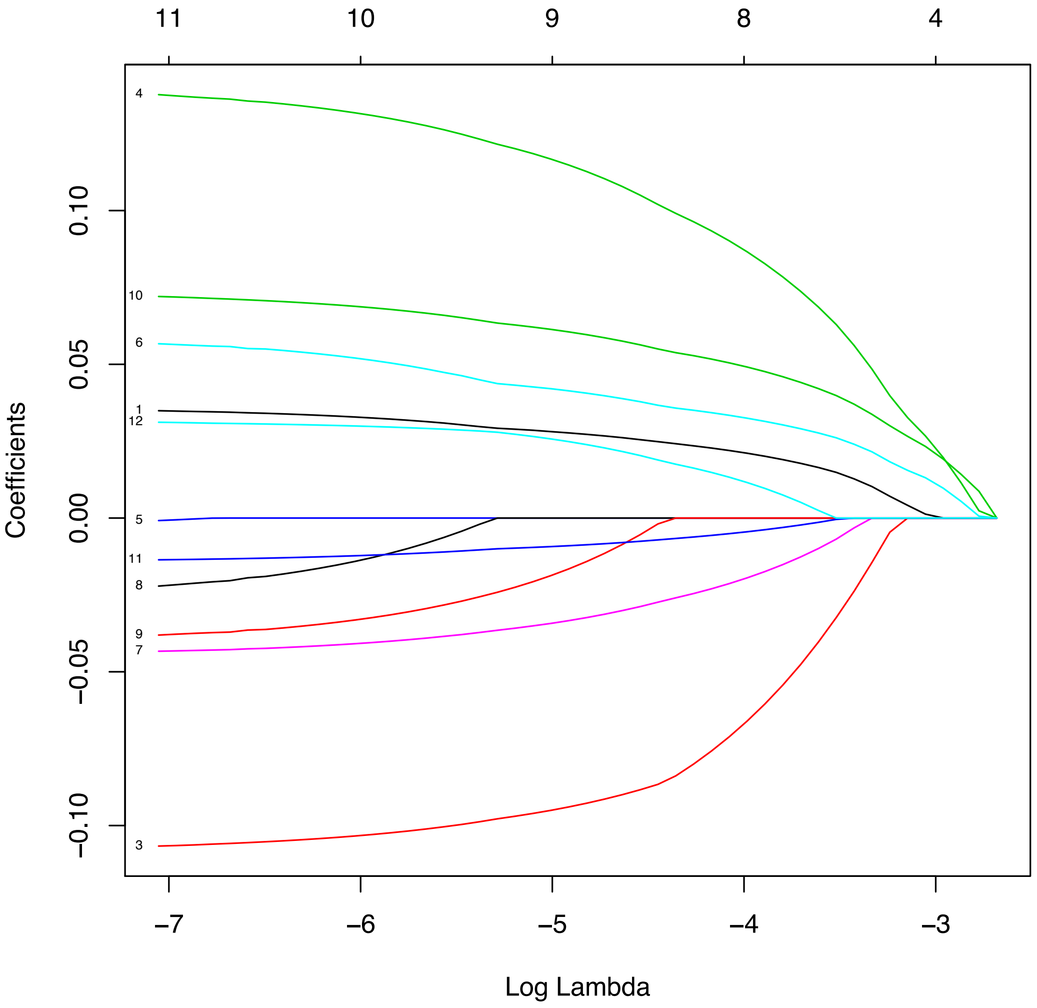
11
10
9
8
4
4
10
6
1
12
5
11
8
9
7
3
0.10
0.05
0.00
−0.05
−0.10
Coefficients
−7
−6
−5
−4
−3
Log Lambda
