# Supplementary material for: Identification of Immune-Related Gene Signatures in Lung Adenocarcinoma and Lung Squamous Cell Carcinoma
Source: Front Immunol. 2021 Nov 23;12:752643. doi: 10.3389/fimmu.2021.752643 (PMC8649721; doi:10.3389/fimmu.2021.752643)

Supplementary figure 1

The profile and correlation of immune cells in LUAD and LUSC. A. Barplot shows the proportion of 22 immune cell subsets in LUAD tissue samples. Column names of plot were sample ID. B. Barplot shows the proportion of 22 immune cell subsets in LUSC tissue samples. Column names of plot were sample ID. C. Heatmap shows the correlations among 22 immune cells in LUAD, and number in each tiny box indicates the correlation coefficient between two kinds of cells. D. Heatmap shows the correlation among 22 immune cells in LUSC, and number in each tiny box indicates the correlation coefficient between two kinds of cells.

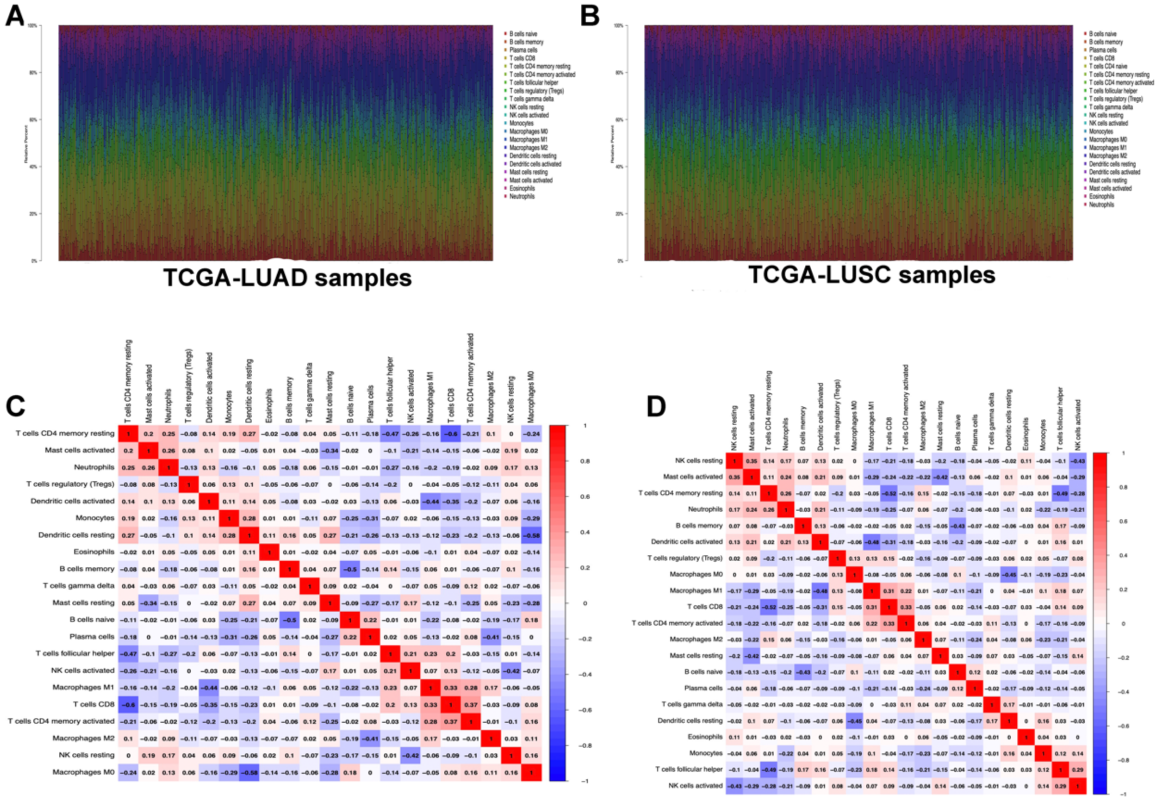

Supplement: Supplementary file 3 [file DataSheet_3.zip › Supplementary figure 1_v1.pdf]
